# The Effectiveness of AI Chatbots in Alleviating Mental Distress and Promoting Health Behaviors Among Adolescents and Young Adults: Systematic Review and Meta-Analysis

**DOI:** 10.2196/79850

**Published:** 2025-11-26

**Authors:** Xinyu Feng, Lidan Tian, Grace W K Ho, Janelle Yorke, Vivian Hui

**Affiliations:** 1 School of Nursing, Hong Kong Polytechnic University, 11 Yuk Choi Road, Hung Hom, Kowloon, 999077, China (Hong Kong), 852 2766 4691; 2 Department of Health and Community Systems, School of Nursing, University of Pittsburgh, Pittsburgh, PA, United States

**Keywords:** artificial intelligence, chatbot, adolescents and young adults, mental health, meta-analysis

## Abstract

**Background:**

The prevalence of mental distress and health risk behaviors among adolescents and young adults has emerged as a pressing public health concern. Artificial intelligence (AI) chatbots have been increasingly recognized for their potential to provide scalable and accessible mental health support and health education; however, questions remain about their effectiveness in addressing the unique challenges faced by adolescents and young adults.

**Objective:**

This study aimed to synthesize evidence from randomized controlled trials (RCTs) on the effectiveness of AI chatbots in alleviating mental distress and promoting health behaviors among adolescents and young adults.

**Methods:**

Eight databases (PubMed, PsycINFO, Cochrane Library, CINAHL, Embase, Web of Science, Scopus, and IEEE Xplore) were searched for RCTs published in English between January 1, 2014, and January 26, 2025. Eligible studies assessed the effects of AI chatbots on mental distress and health behavior outcomes among adolescents and young adults (15‐39 years). Extracted data were synthesized narratively or meta-analyzed as appropriate; subgroup and meta-regression analyses were performed to explore moderators of chatbot effectiveness. Risk of bias was evaluated using the revised Cochrane risk-of-bias 2 (RoB 2) tool for randomized trials. Evidence quality was evaluated using the Grading of Recommendations Assessment, Development and Evaluation (GRADE) approach.

**Results:**

Out of 2495 records retrieved, 31 RCTs were included, comprising 29,637 participants; 26 studies were eligible for meta-analysis. Overall, AI chatbots demonstrated small-to-moderate effects in mitigating mental distress (standard mean difference [SMD] −0.35, 95% CI −0.46 to −0.24; *P*<.001) and promoting health behaviors (SMD 0.11, 95% CI 0.03 to 0.19; *P*=.006) in adolescents and young adults. Significant improvements were observed for depressive (SMD −0.43, 95% CI −0.62 to −0.23; *P*<.001), anxiety (SMD −0.37, 95% CI −0.58 to −0.17; *P*<.001), stress (SMD −0.41, 95% CI −0.50 to −0.31; *P*<.001), and psychosomatic symptoms (SMD −0.48, 95% CI −0.82 to −0.14; *P*=.006); negative affect (SMD −0.27, 95% CI −0.53 to −0.01; *P*=.04); and self-ambivalence and appearance distress (SMD −0.25, 95% CI −0.34 to −0.17; *P*=.01). While AI chatbots contributed to modest enhancements in life satisfaction and well-being, their impacts on positive affect and self-efficacy were limited. The effectiveness of AI chatbots varied depending on target samples, control conditions, and design features such as dialog system methods, deployment formats, and the use of reminders. User engagement emerged as a critical factor for success, with repetitive content and technical issues noted as primary barriers to adherence.

**Conclusions:**

This systematic review and meta-analysis highlights the potential of AI chatbots to address mental health challenges and promote health behaviors among adolescents and young adults. Retrieval-based dialog systems demonstrated consistent and reliable effects, while generative systems showed promise, but their overall effectiveness was inconclusive. Future research should prioritize developing safety protocols and evaluation frameworks for generative systems and validating their long-term impacts on mental health and behavior change in adolescents and young adults.

## Introduction

Adolescents and young adults experience high rates of mental distress, with substance use and mood-related and anxiety disorders being among the most prevalent issues [1]. Significant mental distress triggered by the challenges encountered during this transitional stage in life, such as financial instability, interpersonal relationships, and career development [2], has been implicated in adolescents and young adults’ decreased quality of life and increased suicide risk [3]. Adolescents and young adults also exhibit elevated rates of health-risky behaviors, such as poor dietary choices, inadequate sleep, and physical inactivity [3]. These behaviors are intricately linked with biological and psychosocial factors, including neurological changes, adverse childhood experiences, and peer pressure, which in turn exacerbate the incidence of chronic disease and mental distress among adolescents and young adults [4]. Despite these alarming trends, adolescents and young adults are less likely to seek health support, particularly for sensitive topics such as sexual and physical abuse, sexually transmitted infections and HIV, contraception methods, and substance use [5]. The majority of adolescents and young adult clinical patients reported unmet supportive care needs, with psychological needs being the most frequently cited, followed by needs of physical and daily living [6,7]. Moreover, traditional pediatric and adult interventions are predominantly disease-centric and often fail to address the nuanced, age-specific needs of adolescents and young adults [8]. Unlike children, whose parents typically make health care decisions on their behalf, or mature adults, who are expected to independently manage their appointments and treatments, adolescents and young adults occupy a transitional phase that shares characteristics with both groups but fully aligns with neither [9]. They have limited experience navigating health care systems or seeking external support, while simultaneously grappling with issues of identity, independence, and major life milestones [9]. These challenges highlight significant gaps in current promotive efforts targeting adolescents and young adults, which often struggle to provide effective, age-appropriate care due to workforce shortages and time constraints, underscoring the urgent need for tailored, flexible interventions that can address the complex and diverse health needs of this population [10].

Chatbots are innovative digital tools that simulate conversations with users through a dialog interface, generating responses based on stored patterns [11]. Emerging evidence suggests that chatbots can effectively mitigate symptoms of mental health problems and encourage positive health behaviors [12,13]. For instance, studies have highlighted the efficacy of chatbot interventions in delivering cognitive-behavioral therapy, mindfulness-based practices, and motivational interviewing techniques for people with psychological distress and drug addiction [14,15]. Moreover, chatbots have also been shown to improve user adherence and satisfaction with treatment, which could be essential factors in achieving sustained long-term health outcomes [16,17]. Adolescents and young adults are particularly well-positioned to benefit from chatbots, given their favorable attitudes and openness to innovative health care solutions [18]. This population often experiences increased vulnerability related to identity formation, academic pressures, and relationship dynamics, while simultaneously possessing strong self-directed learning abilities and a preference for autonomy, making them more receptive to digital health solutions compared to children and older adults [19]. Autonomous chatbots hold a unique advantage by being perceived not only as easily accessible and nonjudgmental [20], but also as capable of fostering a sense of peer support, which is a critical source of empowerment that provides invaluable information and psychological solace to adolescents and young adults [10].

Existing reviews on the effectiveness of chatbots in health care have primarily focused on general populations, with limited focus on adolescents and young adults [12,13]. A recent randomized controlled trial (RCT) found that adolescents and young adult users often perceived the chatbot content as irrelevant or too generic, largely due to insufficient tailoring to personal needs [21]. Given the unique developmental, social, and technological contexts that characterize this demographic, it is necessary to systematically evaluate the evidence regarding chatbot interventions targeting adolescents and young adults. Moreover, the diversity in chatbot designs and targeted health outcomes requires a comprehensive synthesis to uncover limitations and highlight areas for future research within this population. Present studies often conflate chatbots with other types of conversational agents, such as voice-based virtual agents, embodied avatars, and social robots [22,23], overlooking the unique advantages of chatbots, particularly their ability to encourage adolescents and young adults to discuss sensitive topics anonymously without fear of judgment. This aspect is often less pronounced in interactions with avatars, robots, or conversations embedded in virtual reality, where social cues may inhibit open communication for those experiencing anxiety or discomfort in social situations [24]. The text-based nature of chatbots not only facilitates rapid information exchange but also allows users to read and review content repeatedly with unlimited, round-the-clock access. This feature enables users to process and reflect on information at their own pace and take positive actions, as it removes the pressure of maintaining a continuous dialog or responding in real time [10]. Furthermore, chatbots stand out for their accessibility and cost-effectiveness, as they can be deployed on commonly used platforms such as smartphones and tablets. This eliminates the need for expensive equipment or immersive environments, significantly enhancing their reach and usability and making them widely available to users across diverse socioeconomic backgrounds and settings [25].

Generative artificial intelligence (AI) has brought chatbots like ChatGPT (OpenAI Inc) and Llama (Meta Inc) to the forefront of digital health innovation. These advanced systems, powered by natural language processing (NLP) and large language models, offer enhanced capabilities for processing complex information, enabling more human-like and adaptive responses to self-care needs [26]. Such flexibility better positions chatbots as promising tools, particularly beneficial for adolescents and young adults who may not proactively seek support from health care professionals or prefer to self-manage their health conditions. At present, there is no established gold standard for engineers to assess the development of chatbots and the quality of information they provide. There is also a lack of systematic evidence regarding their effectiveness for adolescents and young adults across various dialog systems (ie, rule-based, retrieval-based, or generative) and design features (eg, modalities, reminders, and frequency of sessions). These knowledge gaps must be addressed to effectively inform and guide future advancements in the field of chatbot development for health care applications for adolescents and young adults. This systematic review and meta-analysis aims to synthesize the evidence from randomized controlled trials (RCTs) to evaluate the effectiveness of AI chatbots in alleviating mental distress and promoting health-related behaviors among adolescents and young adults. Additionally, this study summarizes key design features of chatbots and examines how these characteristics may moderate intervention outcomes through subgroup analyses and meta-regression. User engagement and experiences with chatbot interactions are also explored and synthesized narratively. By addressing these objectives, the review seeks to provide valuable insights for the development and integration of innovative chatbot-based health care solutions, thereby supporting the enhancement of well-being among adolescents and young adults worldwide. The review questions are as follows:

1. What is the effectiveness of chatbots in alleviating mental distress and promoting health behaviors among adolescents and young adults?
2. What are the key design features of chatbots, and how do these features impact health outcomes in adolescents and young adults?
3. How do adolescents and young adults engage with chatbots, and what are their perceptions and experiences during these interactions?

## Methods

### Protocol Registration and Study Design

The review protocol was prospectively registered in PROSPERO (International Prospective Register of Systematic Reviews), CRD42024603472, and adhered to the PRISMA (Preferred Reporting Items for Systematic Reviews and Meta-Analyses) 2020 (Checklist 1).

### Data Sources and Search Strategy

We conducted a systematic search across 8 databases (PubMed, PsycINFO, Cochrane Library, CINAHL, Embase, Web of Science, Scopus, and IEEE Xplore) using a wide array of search terms (Table S1 in Multimedia Appendix 1). Both subject headings (eg, Mesh and Emtree) and free-text keywords related to the core concepts, along with their synonyms and variants, were included. Additionally, the reference lists of previous reviews [12,27] and the included original studies were manually examined to identify any further eligible studies. The search covered all data from January 1, 2014 to January 26, 2025. This timeframe was selected because the chatbot powered by NLP and machine learning beyond simple rule-based systems began to have significant development and application in health care. This period also coincides with the widespread adoption of internet-connected mobile devices among adolescents and young adults, a group uniquely shaped by and deeply embedded in this digital landscape, ensuring that the evidence included is both technologically relevant and contextually appropriate to their experiences and behaviors. We fine-tuned our search strategy based on previous systematic reviews [12,27] to locate sources related to chatbots for alleviating mental distress or promoting health-related behaviors. The search was limited to English-language publications. After removing duplicates, 2 reviewers screened all titles and abstracts for eligibility independently. Subsequently, the full-text review was also performed by 2 reviewers, with any disagreements resolved through consultation with a third reviewer.

### Eligibility Criteria

We developed our eligibility criteria based on the population, intervention, comparison, outcome, study design (PICOS) framework (Table 1):

1. Population: adolescents and young adults, typically characterized as individuals aged between 15 and 39 years [28], in both clinical and nonclinical samples. Given varying definitions of adolescents and young adults by age and to ensure comprehensive inclusion of related studies, we included original research articles if over 50% of participants fell within the 15‐39 years age range, the average age of participants was within this range, or the study explicitly identified its population as “adolescents and young adults.”
2. Intervention: 2-way interactive chatbots designed primarily to alleviate mental distress or promote health behaviors. These chatbots should operate autonomously without human assistance and serve as the primary component of interventions irrespective of dialog initiatives, interaction modalities, platforms, and settings, but should not be embedded as secondary elements within other technologies, such as virtual reality, robots, or virtual avatars. They may have minor supplementary elements (eg, educational materials) or a simple graphical representation (eg, an icon or avatar), but their primary mode of interaction is through written dialog. Studies focused solely on the development or rationale of chatbot technology, without any empirical evaluation of user-chatbot interaction, were excluded.
3. Comparator: any control groups that did not involve chatbot technology, such as active controls (eg, treatment as usual), information controls (eg, e-book), and passive controls (eg, waitlist, assessment-only).
4. Outcome: eligible primary outcomes included mental health outcomes specified in the *Diagnostic and Statistical Manual of Mental Disorders, Fifth Edition (DSM-5)* [29], as well as health behaviors, defined as actions taken by individuals that affect health or mortality, such as substance use, physical activity, and dietary habits [30]. Metrics related to user engagement with chatbots (eg, retention rates and frequency of interactions) and user experience (eg, satisfaction, acceptability, and usability) were also concluded when reported alongside primary outcomes.
5. Study design: RCTs. Studies were excluded if they were conference abstracts, preprints without peer review, or if the full text was unavailable. Publications that did not present original research findings, including editorials, letters, comments, trial registrations, and study protocols, were also excluded.

**Table 1.** Eligibility criteria (PICOS^a^ framework).

Category	Inclusion criteria	Exclusion criteria
Population	Studies were included if they were about adolescents and young adults, which could be shown by: Over 50% of participants were within 15‐39 years The average age was within 15‐39 years The study explicitly identified its population as “adolescents and young adults.”	Studies that did not report any information about age groups
Intervention	2-way interactive chatbots: With the aim of alleviating mental distress or promoting health behaviors Operating autonomously without human assistance Serving as the primary component of the intervention Primary interaction is through written dialog	Chatbots embedded as secondary elements in other technologies (eg, VR^b^, robots, and virtual avatars) Studies focused solely on development or rationale without empirical evaluation of user interaction
Comparator	Active controls (eg, treatment as usual) Information controls (eg, e-books) Passive controls (eg, wait-list, assessment-only)	Control groups that involved another chatbot technology
Outcome	Primary outcomes: Mental health outcomes specified in the *DSM-5*^c^ [29] Health behaviors (eg, substance use, physical activity, and dietary habits) [30] Secondary outcomes: User engagement (eg,retention rates, frequency of interactions) User experience (eg,satisfaction, acceptability, and usability)	Studies that reported only on secondary metrics without any primary outcomes
Study design	RCTs^d^	Conference abstracts Preprints without peer review Unavailable full text Nonoriginal research (eg, editorials, letters, trial registrations, and study protocols)

a PICOS: population, intervention, comparison, outcome, study design.

b VR: virtual reality.

c *DSM-5: Diagnostic and Statistical Manual of Mental Disorders, Fifth Edition*.

d RCT: randomized controlled trial.

### Data Extraction

We developed a comprehensive data extraction form on Microsoft Excel. The following data were extracted from all included studies: publication details (title, author, and year), study details (study design, region, and recruitment setting), participant characteristics (sample type, sample size, and demographics), chatbot intervention characteristics (name, duration, therapeutic approach, session, and safety measures), and chatbot design features (deployment, delivery platform, dialog system methods, AI technique, and interaction mode). For quantitative analysis, we extracted outcomes and their measures related to targeted conditions, including mental distress (eg, depressive, anxiety, and psychosomatic symptoms), health-related behaviors (eg, physical activity, dietary habits, and substance use). We also extracted and narratively synthesized data related to user engagement (eg, frequency of interactions, number of engaged sessions, and active days) and experience (eg, open-ended feedback, satisfaction, and perceived usability) with chatbots. The data extraction was processed by one reviewer, and then cross-checked by a second reviewer. Any disagreements between reviewers have been resolved through consensus with the involvement of a third reviewer.

### Statistical Analysis

A comprehensive narrative synthesis was conducted to systematically summarize study characteristics, chatbot design features, user engagement metrics, and qualitative findings regarding user experience. This approach involved extracting and thematically analyzing relevant data from included studies to identify patterns, barriers, and facilitators of effective chatbot implementation. To assess the effectiveness of chatbot interventions, we conducted a meta-analysis on RCTs wherein participants were randomly assigned to an experimental group receiving a target chatbot intervention or to a control group. We conducted meta-analyses for overall mental distress and specific symptoms reported by at least 3 trials, including depression, anxiety, positive affect, negative affect, stress, and well-being. Given the focus of included studies spanned a wide range of health-related behaviors, we estimated pooled effect sizes for an overall behavioral health outcome, including sleep-related safety behaviors, stress management, mindfulness, cigarette abstinence, and pain coping. Additionally, general outcomes related to psychological and physical health, such as life satisfaction and self-efficacy, were analyzed as well.

The analyses were conducted using the Review Manager (RevMan; The Cochrane Collaboration) 5.4 [31] and Stata MP 18 (StataCorp LLC) [32]. The standardized mean difference (SMD) with a 95% CI was used to compute the effect size of the continuous statistics as different measurement tools were used for the same outcomes across trials. To combine outcomes reported in continuous and categorical formats, odds ratios were transformed into SMD [33]. Heterogeneity among studies was assessed using the *I^²^* statistic and the Cochran Q statistic. The random effect model was used to account for moderate to high heterogeneity across studies. We calculated SMD using postintervention outcome data that provided means and SDs. When both intention-to-treat and completer analyses were reported, the former was prioritized for analysis. For studies with multiarm designs that included multiple experimental or control groups, we combined the means and SDs from the different arms to create a single pair-wise comparison, as suggested by the Cochrane guidelines for integrating multiple groups from a single study [34]. If a study did not report sufficient data (mean, SD, SE, 95% CI, and sample size) to calculate SMD, we contacted corresponding authors for missing data; studies lacking necessary data were excluded from meta-analysis. For sensitivity analysis, we used a “leave-one-out” method to identify influential studies and assess the robustness of estimates.

We conducted a series of subgroup analyses on the primary outcomes to explore potential moderators. Informed by prior research [12], we examined three study characteristics (ie, control group types, intervention duration, and target sample), as well as four chatbot features (ie, dialog system methods, reminders, interaction mode, and deployment formats) as potential moderators of intervention effects. Specifically, we explored three types of control group (ie, active, information, and passive controls), considering that differences in the nature of participant engagement could influence observed effect sizes; intervention duration was examined as it may impact the sustainability of chatbot effects; the target sample (ie, clinical, subclinical, and nonclinical) was included to account for baseline differences in health status that could moderate intervention outcomes [12]. In addition, 3 primary dialog system methods for input processing and response generation were examined: rule-based, retrieval-based, and generative models [35]. Rule-based chatbots operate on a predefined set of rules, producing predictable responses that are inherently limited in scope. Retrieval-based chatbots select responses from a predefined database of possible answers, enabling some level of contextual understanding while remaining constrained by the availability of their resources. Generative chatbots learn patterns from large datasets and create new, dynamic content, offering greater flexibility to handle diverse and complex conversations [35]. Further, we classified chatbots as those with reminders or those without. Chatbot reminders can serve various functions, including login prompts, system greetings, and mood tracking notifications. For interaction modes, we differentiated between chatbots delivering text-only interactions and those incorporating multimedia materials, such as videos or images. Finally, for deployment, we categorized chatbots as either standalone apps or web-based tools, with the latter being integrated into instant messengers or accessed via websites. Additionally, meta-regression analyses were conducted for continuous variables (ie, gender) when there were at least 10 observations available [34]. Funnel plots and Egger test were used to explore publication bias for meta-analyses that involved more than 10 studies [34]. *P*<.05 was set as statistically significant.

### Quality and Risk of Bias

The Cochrane risk of bias tool (ROB 2) was used to assess the risk of bias in the included RCTs. This assessment tool evaluates 5 domains of potential bias: randomization process, deviations from the intended interventions, missing outcome data, measurement of the outcome, and selection of the reported result. For each domain, a trial can be categorized as having a low risk, some concerns, or a high risk of bias. For the overall risk-of-bias judgment, a trial was deemed to have a low risk of bias only if all domains were rated as low risk. Conversely, any trial was judged to have a high risk of bias if it scored high in any domain. We used GRADEpro GDT software (Evidence Prime, Inc) to evaluate the quality of evidence from meta-analyses, which could be reduced based on 5 key factors: risk of bias, inconsistency, indirectness, imprecision, and publication bias.

## Results

### Search Results

**Figure 1.**
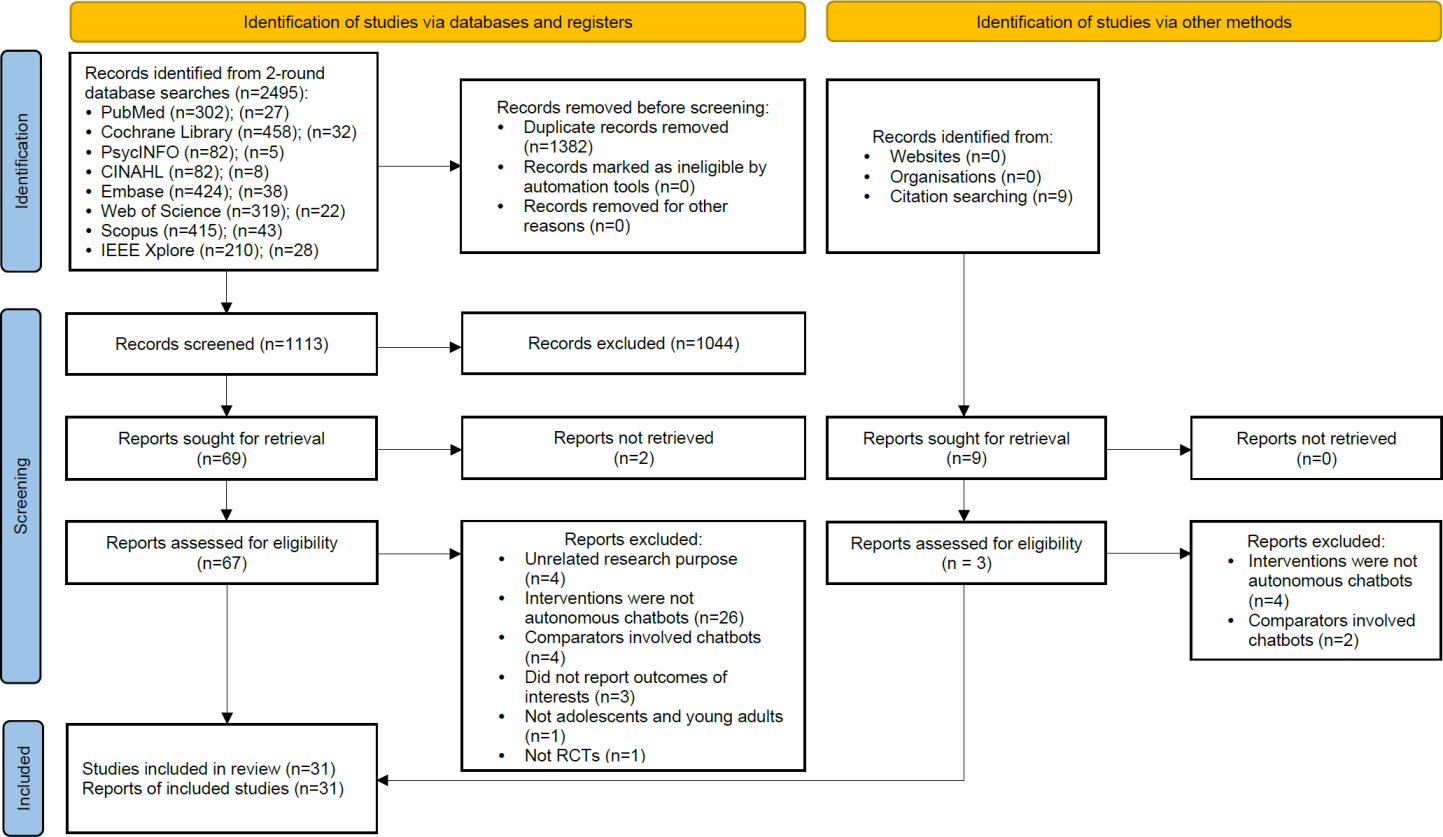
Preferred Reporting Items for Systematic Reviews and Meta-Analyses flow chart. RCT: randomized controlled trial.

Searches of 8 databases identified 2495 unique citations (Figure 1). After removing duplicates, we excluded 1113 records based on titles and abstract screening, resulting in 69 records for full-text review. We additionally included 3 eligible trials identified through reference lists of previous reviews and original studies. A total of 31 studies [14-17,21,25,36-60] met the inclusion criteria and were included in the systematic review for narrative synthesis. Among the 31 studies, 5 randomized trials [14,15,36-38] did not report sufficient data for calculating the pooled effect size; thus, 26 randomized trials were included for meta-analysis [16,17,21,25,39-60].

### Results of Systematic Review

A total of 29,637 participants from 18 countries and regions were involved in 31 studies [14-17,21,25,36-60], recruited from clinical settings (n=4), community (n=10), online (n=10), and mixed settings (n=7). The majority (n=19) had sample sizes under 200 adolescents and young adults. Most were single-site studies, with 10 [15-17,38,39,43,47,52,57,60] conducted in the United States, 5 in China [14,21,45,51,58], and only one [25] multisite study conducted in Switzerland, Germany, and Austria. Among the 31 studies, 12 involved nonclinical populations [15,38,40-42,50,52-56,58], 11 included participants with health problems via self-report or screening (eg, anxiety, depression, or substance use) [17,21,25,36,37,43-46,49,51], and 8 studies involved clinical samples with diagnosed mental or physical health issues [14,16,39,47,48,57,59,60]. Eighteen studies explicitly demonstrated their research focus on adolescents and young adults [15,17,21,36,39-46,49,50,52,53,56,57], one of which focused on young cancer survivors [39], and 4 studies exclusively supported women with specific circumstances, such as intimate partner violence, pregnancy, and childbirth [36,43,56,60]. Intervention duration varied considerably, from several minutes to 4 months, with 15 studies conducting additional follow-up surveys from 2 weeks to 6 months [14,16,21,37-39,43-45,47,49-51,57,58]. Table S2 in Multimedia Appendix 1 presents the characteristics of studies included in this review.

We extracted data on the characteristics of the chatbot intervention and their technical design features (Table S3 in Multimedia Appendix 1). These chatbots were most commonly designed to improve depressive and anxiety symptoms, which were assessed in 20 [15,17,21,25,36,39,41-43,45-49,51,53,57-60] and 19 studies [15,17,21,25,39,41-43,46-49,51,53,56-60], respectively, followed by 7 studies targeting stress management [25,37,41,47,48,50,55]. Specifically, several studies delivered psychotherapy or behavior support for people who experienced substance use and addiction (n=4) [14,16,42,50], self-ambivalence and appearance distress (n=3) [43,44,54], attention-deficit or hyperactivity disorder (ADHD) (n=2) [48,59], sleep disorder (n=2) [51,58], relationship and social activity problems (n=2) [36,38], and eating disorder (n=1) [43]. Cognitive behavioral therapy was the most common therapeutic approach (n=21) [15,17,21,25,41-49,51-55,57,58,60], followed by mindfulness-based therapy (n=9) [14,15,37,40-42,45,48,59], motivational interviewing (MI) (n=5) [15,38,42,50,53], stress coping (n=4) [37,39,47,59], acceptance and commitment therapy (n=3) [15,37,54], interpersonal psychotherapy (n=3) [15,57,60], dialectical behavior therapy (n=3) [42,57,60], positive psychology (n=2) [39,55], and emotion-focused therapy (n=2) [15,53]. In addition to the core treatment, other notable design features included empathic responses, customization, mood tracking, reflection, accountability, goal-setting, mascot or static avatars, gamified interaction, and problem-solving. Seven studies were tailored to address key challenges unique to adolescents and young adults, such as academic work management, life transitions, relationships [40-42], body image concerns [43,45], and self-esteem issues [45,46], which were particularly salient during this developmental stage.

Regarding the design characteristics of chatbots, instant messenger platforms (ie, Facebook [Meta Platforms], WeChat [Tencent Holdings Limited]) and standalone smartphone apps emerged as the most popular platforms for delivering chatbot services, featured in 15 [14-17,21,36,37,39,43-45,49,53,54,58] and 13 studies [25,40-42,46-48,50,51,55,57,59,60], respectively. The remaining 3 studies deployed the chatbots on websites [38,52,56]. Most of the chatbots provided periodical pop-up notifications to remind users to interact with chatbots (n=22). 21 studies integrated auditory or visual content based on text-based generation [14,17,21,25,36-45,47,48,50,52,54,55,57]. Eighteen studies incorporated safety measures in chatbots, such as access to human professionals, a crisis hotline, suicidal ideation monitoring, and referral to local resources [14,15,17,21,25,36,42-44,46-49,53,57-60]. The majority of chatbots (n=18) used a rule-based approach to interact with users [17,25,37-39,41-44,48,50,51,54,55,57-60], while 10 studies used a retrieval-based system [14-16,21,40,45,47,49,53,56]. Only 3 studies explored generative approaches for chatbot development, using Bidirectional Encoder Representation from Transformers (BERT) and GPT to create real-time responses [46,52,58], and one study used GPT-3.5 to refine the chatbot following its pilot testing phase [16]. In terms of AI techniques, NLP was used in most studies (n=12) to analyze user intent and context, facilitating the selection of appropriate responses [14,15,17,21,40,45,48,52,53,56,57,60]. Additionally, some reports integrated other methodologies, including machine learning (n=7) [14-16,21,49,57,60], natural language understanding (n=5) [16,21,40,47,49], and deep learning (n=3) [14,45,52], to enhance the chatbots’ learning capacity and contextual comprehension.

Usage data and user engagement with chatbots were tracked in 23 studies through various metrics, including the frequency of interactions or exchanged messages (n=11) [15,16,25,40,41,43,45,49,53,57,60], the number of engaged sessions or completion rates (n=9) [25,39,41,44,45,47,50,51,56], the length of conversations (n=7) [39,41,43,45,48,49,56], the number of active days (n=6) [16,40,43,48,55,57], the number of check-ins (n=3) [17,48,57], and the time period for peak use (n=1) [45]. More than half of the studies (n=17) reported higher than 20% attrition in the intervention group [14,21,25,36,37,39-41,43,44,47,50,51,53,54,58,60]. Two studies analyzed the change in performance of user engagement over a time period [21,40]. Additionally, 24 studies explored user experiences, using metrics such as satisfaction (n=8) [14,15,17,21,45,48,52,60], helpfulness (n=5) [14,39,43,46,50], working alliance (n=5) [21,25,45,49,60], and acceptability (n=4) [44,45,49,57]. Open-ended user feedback was documented in 14 studies [15-17,21,25,39,41,45,48,49,53,55,56,58], providing valuable insights into both the strengths and limitations of chatbot interactions. On the positive side, chatbots were frequently praised as effective tools for promoting understanding and awareness of health topics through structured exercises and detailed explanations (n=6) [15,17,25,48,56,58]. Users valued chatbots for their empathy, emotional support, and ability to foster a sense of being heard (n=6) [15,17,21,45,48,58]. Personalization and ease of access were commonly highlighted (n=4) [17,21,41,45] with chatbots regarded as a convenient alternative to traditional therapy [39]. Features such as reminders, weekly summaries, and visually engaging elements like emojis, avatars, and interactive interfaces enhanced the user experience, contributing to adherence and helping users stay on track with their health goals (n=3) [41,48,55]. However, notable challenges were also identified, with repetitive and rigid interactions emerging as a major concern (n=10) [15,17,21,25,41,45,48,55,56,58]. Users expressed frustration over the inability of chatbots to handle open-ended or unexpected responses (n=6) [15-17,41,49,53], and some conversations were criticized for being overly general or lacking depth and clarity (n=5) [17,21,55,56,58]. Technical issues, such as glitches, looping conversations, and slow operations, were frequently reported (n=7) [14,17,37,41,45,56,58], disrupting the interaction flow and significantly diminishing overall usability.

Of the 31 studies, only one study reported mediators between chatbot interventions and outcomes, in which visceral anxiety, catastrophic thinking, and fear of food were observed to be significant mediators between chatbot use and gastrointestinal symptom severity (*P*<.001) and quality of life (*P*<.001) [47]. For moderators, one study revealed significant interaction effects of group by ethnicity and by writing behaviors for social activity, stress, and life satisfaction [38]. Two studies noted that people with more severe baseline physical and mental health symptoms experienced more pronounced benefits of chatbots [44,47]. Four studies probed the moderating role of user engagement. Specifically, the frequency or the number of times of interaction with the chatbot was positively correlated with the reduction in ADHD symptoms (*P*=.03) [48] and loneliness (*P*<.006) [49]. The dosage, measured as engaged sessions, was correlated with improvement in anxiety (*P*=.06) [38], and depression (*P*=.08), quality of life (*P*=.07) [47]. Another study revealed that the reported commitment to change behavior significantly increased with time (*P*<.001), suggesting higher commitment toward the end of the intervention than in the middle or at the start [25].

### Results of Meta-Analysis

#### Overall Mental Distress

**Figure 2.**
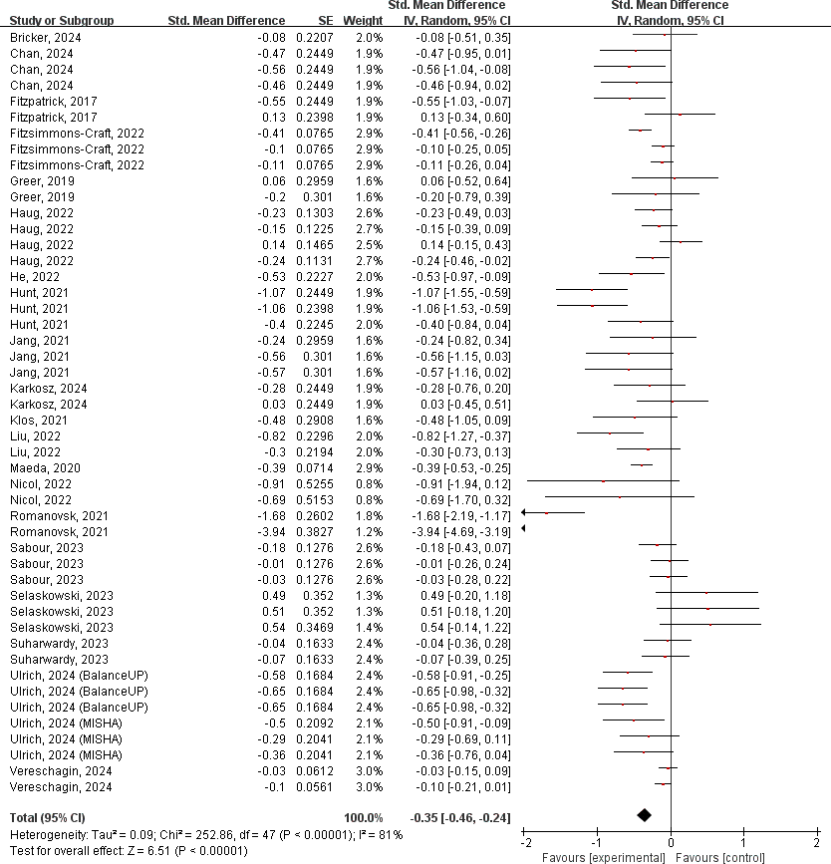
Forest plot for the effects of chatbots on overall mental distress. [16,17,21,25,39,41-43,45-51,53,56-60]

A total of 21 studies, comprising 2813 participants in the experimental groups and 3116 in the control groups, were included in the meta-analysis for the overall mental distress. Among these, indicators for anxiety (n=18) [17,21,25,39,41-43,46-49,51,53,56-60] and depression (n=17) [17,21,25,39,41-43,45-49,51,57-60] were most commonly examined, and the remaining assessments included somatic symptoms (n=3) [25,41,47], sleep disorders (n=2) [51,58], ADHD (n=2) [48,59], substance use disorders (n=2) [16,50], and eating disorders (n=1) [43]. Compared to control conditions, participants interacting with chatbots exhibited significantly greater reductions in the overall mental distress, with an effect size of SMD −0.35 (95% CI −0.46 to −0.24; *P*<.001) (Figure 2). The “leave-one-out” sensitivity analysis demonstrated the robustness of the findings, with estimated effect sizes ranging from −0.30 to −0.36 (Figure S11 in Multimedia Appendix 1). The results of the funnel plot and Egger test revealed potential publication bias (*P*=.01), while no additional studies were imputed with the Trim-and-Fill approach and the adjusted effect size (SMD −0.372, 95% CI −0.529 to −0.216) was identical to the observed value, suggesting a negligible impact on the conclusions. The subgroup analyses revealed 4 significant moderators. Studies that targeted subclinical and clinical samples produced larger effect sizes than those for nonclinical populations (*P*=.003). Chatbots deployed as standalone apps were significantly more effective than those delivered via instant messenger or websites (*P*=.03). Among different chatbot architectures, generative chatbots demonstrated the largest effect size, followed by retrieval-based and rule-based systems (*P*=.007). Interestingly, studies comparing chatbots to active control did not show significant group differences, and their pooled effect was significantly lower than those comparing chatbots to information and passive controls (*P*=.02). The detailed results of subgroup analysis are presented in Table S4 in Multimedia Appendix 1.

#### Depression

The pooled effect size for the 17 postintervention comparisons between chatbots and various control conditions on depression was (SMD −0.43, 95% CI: −0.62 to −0.23; *P*<.001), with high heterogeneity (*P*<.001; *I^2^*=81%) (Figure S1 in Multimedia Appendix 1). The sensitivity analysis demonstrated the robustness of the findings, with estimated effect sizes ranging from −0.34 to −0.47 (Figure S11 in Multimedia Appendix 1). The results of the funnel plot and Egger test revealed potential publication bias (*P*=.02), while no additional studies were imputed with the Trim-and-Fill approach and the adjusted effect size (SMD −0.44, 95% CI −0.66 to −0.21) was identical to the observed value, suggesting a negligible impact on the conclusions. Subgroup analyses revealed a significant difference between dialog system methods (*P*=.03). Specifically, retrieval-based chatbots demonstrated the strongest and most reliable effect, followed by rule-based chatbots with a smaller but significant effect (*P*<.001). Generative chatbots, while showing a potentially large effect, exhibited a wide CI and failed to reach statistical significance (Table S4 in Multimedia Appendix 1).

#### Anxiety

A total of 18 studies were included for the effects on anxiety [17,21,25,39,41-43,46-49,51,53,56-60]. Compared to the control groups, participants interacting with chatbots exhibited a significantly greater reduction in anxiety, with an effect size of SMD −0.37 (95% CI −0.58 to −0.17; *P*<.001) ( Figure S2 in Multimedia Appendix 1). The heterogeneity was considerably high across included trials (*P*<.001; *I^2^*=87%). The sensitivity analysis revealed a stable pooled effect size ranging from −0.35 to −0.41 and remaining statistically significant when an influential study was excluded [46] (Figure S11 in Multimedia Appendix 1). There is no significant publication bias as supported by the funnel plot and Egger test (*P*=.18). The subgroup analyses highlighted significant differences in chatbot effectiveness between deployment formats (*P*=.05). Specifically, standalone chatbots produced higher between-group effects on anxiety compared to those delivered via instant messenger or website (Table S4 in Multimedia Appendix 1).

#### Positive Affect

There is no statistically significant effect of chatbot interventions observed on positive affect compared to controls (SMD 0.03, 95% CI: −0.15 to 0.21; *P*=.73), with substantial heterogeneity across 11 studies (*P*=.002; *I²*=63%) (Figure S3 in Multimedia Appendix 1). The pooled effect sizes remained relatively stable with confidence intervals consistently crossing the null value after sequentially omitting each study (Figure S11 in Multimedia Appendix 1). The funnel plot showed a symmetrical pattern with data points scattered evenly around the pooled effect size, suggesting the absence of marked small-study effects, which was further confirmed by the Egger test (*P*=.55).

#### Negative Affect

A small but statistically significant decrease in negative affect among participants who used chatbots compared to controls (SMD −0.27, 95% CI=−0.53 to −0.01; *P*=.04) was observed among 11 studies (Figure S4 in Multimedia Appendix 1). All estimated effect sizes yielded from sensitivity analysis consistently fell within the 95% CI, ranging from −0.26 to −0.31 (Figure S11 in Multimedia Appendix 1). The heterogeneity significantly decreased from an *I*^2^ value of 83% (*P*<.001) to 0% (*P*=.84) when we excluded the study by Romanovskyi et al [46], though the overall effect remained significant. The funnel plot was visually symmetrical, and the Egger test for small-study effects did not detect significant publication bias (*P*=.39).

#### Stress

Participants engaging with chatbots demonstrated a significantly greater reduction in stress compared to various control conditions, with a moderate effect size (SMD −0.41, 95% CI: −0.50 to −0.31; *P*<.001) (Figure S5 in Multimedia Appendix 1). No heterogeneity (*I^2^*=0%; *P*=.54) was observed across 6 included studies, indicating that the effects of chatbots on stress were consistent and generalizable across studies with differing characteristics. The sensitivity analysis further confirmed the robustness of the findings, with estimated effect sizes ranging from −0.40 to −0.56 (Figure S11 in Multimedia Appendix 1). Specifically, when we excluded the study by Haug et al [50], a slightly larger effect size estimate (SMD −0.56, 95% CI −0.76 to −0.36) was observed. This deviation may be attributed to the inappropriate use of a single-item measure for stress symptoms and a considerably larger sample size compared to other trials. Nevertheless, the overall effect remained statistically significant even when the influential study was excluded.

#### Psychosomatic Symptoms

Five studies assessed psychosomatic symptoms influenced by chatbot interventions, resulting in a significantly larger reduction in various symptoms compared to control groups (SMD −0.48, 95% CI −0.82 to −0.14; *P*=.006) (Figure 6 in Multimedia Appendix 1). The sensitivity analysis indicated the robustness of the findings, with estimated effect sizes ranging from −0.36 to −0.49 (Figure S11 in Multimedia Appendix 1). The heterogeneity among included studies was considerable (*P*=.002; *I²*=76%), but significantly decreased (*P*=.20; *I²*=35%) after we excluded the study by Sabour et al [58] while the overall effect remained the same direction and significance. Subgroup analyses revealed three significant moderators. Specifically, studies that targeted clinical samples showed a greater decrease in psychosomatic symptoms than those focusing on subclinical and nonclinical samples (*P*=.008). Chatbots deployed as standalone apps yielded significantly greater effects than web-based platforms (*P*=.002). Additionally, retrieval-based systems showed the largest effects, outperforming both generative and rule-based chatbots (*P*=.001) (Table S4 in Multimedia Appendix 1). However, these results should be interpreted with caution due to the limited number of studies available for each subgroup.

#### Self-Ambivalence and Appearance Distress

Four distinct measures targeted negative self-relevant thoughts and body image were included for evaluating the influence of various interventions on self-ambivalence and appearance distress in this analysis. A significant positive effect favoring chatbots was observed compared to passive control groups (SMD -0.25, 95% CI −0.34 to −0.17; *P*<.001), with moderate heterogeneity across studies (*P*=.19; *I²*=38%) (Figure S7 in Multimedia Appendix 1). The pooled estimates remained statistically significant, with the overall effect size ranging from −0.20 to −0.31 and within comparable confidence intervals (Figure S11 in Multimedia Appendix 1).

#### Life Satisfaction and Well-Being

Ten relevant outcomes from 7 separate trials were meta-analyzed for the overall life satisfaction and well-being. A significantly greater improvement for participants in the chatbot groups was observed than those in controls (SMD 0.12, 95% CI 0.03-0.21; *P*=.01), with moderate heterogeneity detected across 7 trials (*P*=.06; *I²*=44%) (Figure S8 in Multimedia Appendix 1). The sensitivity analysis suggested the robustness of the findings, with the overall effect sizes ranging from 0.07 to 0.13 ( Figure S11 in Multimedia Appendix 1). However, when we excluded two influential studies [25,42], the 95% CI crossed the null value, while the direction maintained the same. The absence of publication bias was evidenced by the funnel plot and Egger test (*P*=.76). Subgroup analyses revealed a significant difference in effects between dialog systems (*P*=.04) (Table S4 in Multimedia Appendix 1). Moreover, meta-regression analysis revealed statistical effects of gender (*P*=.02) on the pooled effect size (Figure S12 in Multimedia Appendix 1).

#### Self-Efficacy

Six trials were included in the meta-analysis to evaluate the pooled effect of chatbot interventions on self-efficacy outcomes, resulting in a positive trend effect favoring the experimental group but no statistically significant difference obtained (SMD 0.14, 95% CI −0.14 to 0.41; *P*=.33) (Figure S9 in Multimedia Appendix 1). Considerably high heterogeneity was observed across the included studies (*P*<.01; *I²*=86%), which may be attributed to differences in specific measurement targets, encompassing general self-efficacy, self-efficacy in addressing body image concerns, and confidence in self-management for health and well-being. The results of the sensitivity analysis showed that the overall effect remained stable, with SMD estimates ranging from 0.10 to 0.26, and the pooled effect remaining statistically nonsignificant when individual studies were excluded (Figure S11 in Multimedia Appendix 1).

#### Health Behavior Change

**Figure 3.**
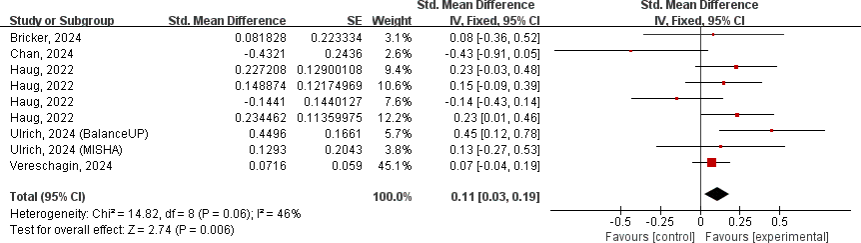
Forest plot for the effects of chatbots on health behavior change. [16,25,41,42,50,51]

Nine health behavior outcomes from 6 separate trials were included for the meta-analysis, revealing a statistically significant effect in favor of chatbot interventions (SMD 0.11, 95% CI 0.03-0.19; *P*=.006) (Figure 3). Moderate heterogeneity among studies was observed among studies (*P*=.06; *I²*=46%), potentially attributed to the wide spectrum of health behaviors we targeted. Sensitive analyses demonstrated the robustness of this result, with estimates ranging from 0.09 to 0.14 (Figure S12 in Multimedia Appendix 1). Notably, the omission of 2 specific outcomes [50,51] resulted in a slight increase in the combined effect size and significantly decreased the heterogeneity. The symmetric funnel plot and Egger test (*P*=.43) indicated a low likelihood of publication bias. Studies designed with active controls produced less between-group effects than those compared to a passive control group (*P*=.02). Additionally, chatbots that sent check-in reminders produced more positive effects on changing behaviors than those that did not (*P*=.02) ( Table S4 in Multimedia Appendix 1).

### Quality and Risk of Bias

The interrater reliability, as measured by Cohen kappa, ranged from 0.471 to 0.523 across 5 domains of the Cochrane ROB 2 tool, indicating moderate agreement between the raters. For any discrepancies identified between raters, discussions were held to achieve consensus; if consensus could not be reached, a third reviewer was consulted to make the final decision. The overall risk of bias was rated as high for 25 studies (Figure S13 in Multimedia Appendix 1). The majority of studies (26/31) demonstrated appropriate randomization procedures and were rated as low risk in the domain of randomization process. However, 5 studies raised concerns due to insufficient reporting on the random allocation approach or observed imbalances in baseline characteristics between groups. For the domain of deviation from the intended interventions, no studies exhibited significant deviations from the intended interventions, though neither participants nor those delivering the interventions could be blinded due to the nature of the intervention. 19 studies adhered to the ITT principle. However, 8 studies were judged to raise some concerns in this domain due to the absence of appropriate analyses to estimate the effect of assignment to the intervention. Additionally, 7 studies were rated as high risk because a substantial proportion of participants were excluded from the analyses, which could have significantly impacted the validity of the results. 12 studies were judged to have a low risk in the domain of missing outcome data, while 14 were rated as high risk due to imbalanced drop-out rates between groups and lack of evidence that appropriate methods were used to address the potential bias introduced by high attrition. The primary reason for the notable source of bias arising from the measurement of the outcome was the reliance on self-reported outcomes as the preferred method in most trials, where 16 studies were rated as high risk because self-reported measures are inherently prone to biases, and the strong level of belief in the beneficial effects of the intervention could influence outcome assessments. In the selection of the reported result domain, 12 studies raised some concerns due to the unavailability of their protocols or trial registrations, or minor discrepancies between the planned and reported outcome measurements. Furthermore, 2 studies were judged to have a high risk as their reported results were likely selected from multiple eligible measures or analyses, raising concerns about selective reporting. The quality of evidence, evaluated using the GRADE approach, was rated as very low to low, possibly due to the overall high risk of bias or substantial heterogeneity across the majority of studies (Table S5 in Multimedia Appendix 1).

## Discussion

### Principal Findings

In this systematic review and meta-analysis, we synthesized evidence on the effectiveness of chatbots for adolescents and young adults and found overall significant positive effects in alleviating mental distress and promoting health behavior change. The most pronounced effects were observed in studies that compared chatbot interventions to information controls, used standalone mobile apps for deployment, used generative or retrieval-based chatbots, or targeted individuals in subclinical and clinical groups. Additionally, chatbots with reminders that encourage users to engage in interactions have been more effective in promoting behavior change. Moreover, user engagement was a significant moderator influencing chatbot effectiveness, while repetitiveness and inflexibility of content emerged as the most common barriers to retain chatbot adherence. Despite the proposed advantages of chatbots as accessible, cost-effective treatment alternatives, none of the studies included in this review conducted cost-effectiveness analyses or focused on low-resource settings.

Across the included studies, chatbots consistently demonstrated small-to-moderate effects in reducing symptoms of depression, anxiety, negative affect, stress, and psychosomatic problems among adolescents and young adults. These findings reinforce prior evidence, underscoring the promise of chatbots as scalable and accessible tools to address specific mental health challenges in this population [12]. Notably, retrieval-based chatbots demonstrated a consistent moderate effect in reducing depressive and psychosomatic symptoms, suggesting that the structured and evidence-based design may offer a more reliable and effective approach to delivering mental health support. In contrast, the comparatively modest effects observed with rule-based chatbots may stem from their inherent limitations in flexibility and reliance on predefined scripts. While rule-based systems can be effective in specific scenarios, their rigid architecture often restricts their ability to adapt to the diverse and dynamic needs of individuals with mental health problems. Generative chatbots, despite showing the strongest effects for overall mental distress, did not demonstrate consistent effects for specific mental health problems, which may be attributed to the limited available evidence. This uncertainty highlights the need for further research to better understand the potential and the limitations of generative chatbots applied in this context. Additionally, our analysis indicated that chatbots were more effective for psychosomatic symptoms in clinical populations compared to nonclinical groups, which aligns with the notable trend across studies that individuals with more severe baseline symptoms tended to derive greater benefits from interventions [44,47]. Moreover, the larger effect size observed for standalone chatbots in alleviating anxiety, compared to web-based ones, indicates that the deployment format may play a crucial role in influencing the effectiveness of chatbots. This may be attributed to the personalized and engaging design of the independent system, allowing for a more focused therapeutic engagement with less interruption, as opposed to chatbots integrated into instant messenger apps or websites that may cause more distractions. In addition, our review is among the first to provide valuable evidence supporting the effectiveness of chatbots in reducing self-ambivalence and appearance distress. While the effect size was modest, this finding is particularly significant for adolescents and young adults, who frequently grapple with issues related to identity, self-esteem, and body image. This highlights the potential of chatbots to address sensitive and deeply personal concerns that individuals may find difficult or shameful to discuss with human professionals. The ability of chatbots to offer a nonjudgmental and accessible platform for support is crucial in this context. However, it is important to note that this synthesized result was derived from four different measures, requiring the need for further research to explore subgroup analyses to provide deeper insights into the specific contexts and conditions under which chatbots are most effective.

A significant but small effect was observed for life satisfaction and well-being, while no statistically significant improvement was noted for positive affect and self-efficacy. These findings align with the result of a previous review [61], which reported limited impacts of conversational agents on fostering positive psychological well-being. This phenomenon may reflect a ceiling effect in certain populations or could be attributed to the primary focus of most therapeutic strategies, which tend to prioritize addressing mental health problems over promoting well-being, resilience, and recovery. This underscores the need for future chatbot designs that incorporate elements based on positive psychology skills, such as acknowledgment of positive events, personal strengths, and gratitude exercises. Moreover, such positive states may require longer-term or more intensive therapeutic sessions to yield measurable improvements. However, insufficient follow-up data for these outcomes can be accessed for validating our assumptions. Furthermore, our findings revealed that studies with a higher proportion of women reported greater improvements in overall well-being. This draws new attention to the possibility that the effectiveness of chatbots may be influenced by gender-related factors, such as differences in communication styles or help-seeking behaviors, with women potentially being more inclined to seek support for mental health issues or to engage in emotional disclosure that may align more closely with the empathetic design of many chatbots [62]. However, it is notable that no study in our review explicitly examined gender differences in user engagement or interaction patterns with chatbots. Two studies [38,52] used Linguistic Inquiry and Word Count (LIWC) to analyze participants’ response transcripts. While indicating a potential relationship between word use frequency and mental well-being, these studies did not identify gender-based differences in expression characteristics. Further research is warranted to explore whether women exhibit stronger adherence to chatbots, or different interaction styles (ie, use of reflective language), and whether these factors serve as mechanisms for boosting therapeutic outcomes.

The effectiveness of chatbots in health behavior changes, though significant, remains relatively small, which aligns with a previous review [13]. Several factors may account for this observation. First, the limited statistical power resulting from the small number of trials (n=5) included may have constrained the ability to detect larger effects. The use of chatbots to encourage physical activities and healthy lifestyles within adolescents and young adults is markedly underreported, remaining a vast scope for further research to evaluate their impact on promoting sustained behavior change. Second, the reliance on self-reported measures introduces inherent biases and inaccuracies, which may compromise the validity of the observed findings. To address this issue, incorporating objective data collection methods, such as wearable devices or biological markers, could enhance the precision and reliability of outcome measurements and provide more robust evidence for behavior change. Third, differences in the theoretical underpinnings used across studies to drive behavioral change could have elicited diverse responses to chatbot interventions. However, due to the small number of original studies included, we are unable to further disentangle these nuanced effects on specific types of health behaviors. Moreover, our analysis revealed that studies using active controls reported smaller effects for chatbots compared to those using passive controls. This suggests that while chatbots may offer unique advantages, their incremental value may be less pronounced when benchmarked against well-established interventions. It is imperative for forthcoming studies to determine whether the chatbot interventions yield greater benefits when integrated as complementary tools rather than being standalone. In addition, regular check-in reminders from chatbots may serve as effective cues to action, reinforcing user engagement and adherence to desired behaviors. Further research is warranted to explore the extent to which the frequency and timing of reminders impact their efficacy.

The diversity in chatbot evaluation methods suggests a critical gap and calls for exploratory research to develop professionally validated instruments for assessing chatbot accuracy, safety, and user experience. The notable attrition rates observed in both groups, coupled with unsatisfactory completion of chatbot sessions, underscore the pressing need to optimize future research design to enhance user engagement and facilitate a more positive experience. To this end, it is imperative to involve adolescents and young adult participants in the chatbot design process, such as surveys, interviews, and user testing, ensuring that the intervention features align with their preferences, expectations, and behavioral patterns [63]. Additionally, optimizing the chatbot’s performance and designing a clear, user-friendly conversational interface are crucial to ensuring a satisfying user experience that promotes sustained engagement. Moreover, generative AI systems present significant opportunities in this regard, with the potential to achieve more flexibility, deeper contextual understanding, and superior response quality, which have demonstrated remarkable user engagement globally [64]. Notably, generative AI chatbots can respond adaptively to unexpected user inputs, even those not previously encountered, and avoid repetitive responses to varied queries, fostering more human-like dialogs that enhance users’ sense of being understood and empathized with. Despite these advancements, the application of chatbots in the domains of psychological and physical health remains cautious. Most therapeutic chatbots currently rely on rule-based or retrieval-based designs. This limitation is primarily due to concerns about the insecurity, potential biases, and “hallucination” of AI-generated content when addressing sensitive issues, which could lead to unintended negative consequences [65]. The “black box” nature of deep learning algorithms makes it impossible to predict conversational trajectories in advance [66]. Retrieval-augmented generation (RAG) offers a promising solution by connecting generative models with real-time information retrieval from external knowledge bases. This approach facilitates secure incorporation of up-to-date information and sensitive data while reducing the likelihood of hallucination and improving the accuracy through context grounding [67]. Graph-based RAG (GraphRAG) demonstrates significant potential for extracting holistic insights from lengthy documents by structuring RAG data into graphs. This enhances the capabilities of large language models to produce evidence-based medical responses, thereby increasing safety and reliability when managing private medical data [68]. Given the unique risks faced by adolescents and young adults, such as disclosure of self-harm intent to chatbots, or the reinforcement of harmful thought patterns by algorithms, it is crucial that research efforts should prioritize the establishment of clear safety protocols and robust evaluation frameworks to ensure their ethical and responsible deployment [69].

### Limitations

While our findings break new ground in exploring the influence of chatbot dynamics on holistic psychosocial well-being, specifically within adolescents and young adult populations, the conclusions are somewhat constrained by several limitations. First, the inclusion of studies with populations that were not exclusively adolescents and young adults but had a mean age within an eligible age range, though necessary to ensure comprehensive coverage of relevant evidence, may have introduced potential variability in contextual factors that may compromise the findings. Second, although the incorporation of diverse participant demographics enhances the ecological validity of the results, the lack of strict clinical thresholds for mental distress at baseline in some studies may dilute the observed intervention effects for clinically significant cohorts. Third, while examining a broad array of outcomes provides valuable insights into the potential of chatbots in health care, the variation in measurement instruments across studies for the same outcomes, as well as the combination of different health behaviors into a single aggregated outcome, may introduce substantial heterogeneity and obscure important distinctions between specific behaviors. Furthermore, due to the limited number of studies with follow-up data on the same outcomes and the wide variability in follow-up durations, it was not feasible to conduct a meta-analysis assessing sustained impacts. Crucially, the majority of included studies were assessed as having a high risk of bias, which may result in misestimation of effect sizes. Consequently, the certainty of evidence for most outcomes was rated as very low to low, substantially restricting both the generalizability and reliability of the observed effects. Moreover, while the adjusted effect sizes for overall mental distress and depressive outcomes appear robust to publication bias, the potential for unpublished negative or inconclusive studies suggests that the true effect of AI chatbots may be smaller than reported. Therefore, the conclusions drawn from this review should be interpreted with considerable caution. Finally, despite the rapid proliferation of generative AI, this review underscores a critical gap in empirical research evaluating their specific impacts among adolescents and young adult populations, which also hindered our ability to provide evidence on the effects of the specific mechanisms of generative models on therapeutic outcomes. The clinical effectiveness of generative AI chatbots in mental and behavioral health remains unknown. Future studies are expected to implement large-scale, long-term interventions with rigorous designs to fully understand the benefits and advantages of chatbots integrated with generative systems.

### Conclusions

This study provides evidence supporting the overall effectiveness of chatbots in alleviating mental distress and promoting positive health behaviors among adolescents and young adults. The effectiveness of chatbots varied across different target samples and control conditions, and three key design features were identified as significant moderators of chatbot efficacy: dialog system methods, deployment format, and the use of reminders. Among the dialog systems, retrieval chatbots demonstrated the most consistent and reliable effects, while generative AI chatbots showed potential but exhibited variability in their effectiveness. Given the growing use of generative AI, it is crucial to establish robust safety protocols and evaluation frameworks before their implementation in real-world settings. Future research should focus on validating the long-term effects and consistency of generative AI chatbots while exploring their broader applications in mental health and behavioral interventions for adolescents and young adults.

## Supplementary material

10.2196/79850 Multimedia Appendix 1 Summary of findings.

10.2196/79850 Checklist 1 PRISMA checklist.

## Abbreviations

ADHD: attention-deficit/hyperactivity disorder
AI: artificial intelligence
BERT: Bidirectional Encoder Representation from Transformer
*DSM-5*: *Diagnostic and Statistical Manual of Mental Disorders, Fifth Edition*
GRADE: Grading of Recommendations Assessment, Development and Evaluation
NLP: natural language processing
PICOS: population, intervention, comparison, outcome, study design
PRISMA: Preferred Reporting Items for Systematic Reviews and Meta-Analyses
PROSPERO: International Prospective Register of Systematic Reviews
RAG: retrieval-augmented generation
RCT: randomized controlled trial
SMD: standardized mean difference
