# Supplementary material for: The Effectiveness of AI Chatbots in Alleviating Mental Distress and Promoting Health Behaviors Among Adolescents and Young Adults: Systematic Review and Meta-Analysis
Source: J Med Internet Res. 2025 Nov 26;27:e79850. doi: 10.2196/79850 (PMC12661615; doi:10.2196/79850)

Summary of Findings

Table S1. Search Strategies 2

Table S2. Summary of characteristics of included studies 11

Table S3. Summary of chatbot design features 21

Table S4. Results of subgroup analysis 36

Table S5. GRADEpro GDT Summary of findings 41

Figure S1. Forest plot for the effects of chatbots on depression 43

Figure S2. Forest plot for the effects of chatbots on anxiety 43

Figure S3. Forest plot for the effects of chatbots on positive affect 43

Figure S4. Forest plot for the effects of chatbots on negative affect 44

Figure S5. Forest plot for the effects of chatbots on stress 44

Figure S6. Forest plot for the effects of chatbots on psychosomatic symptoms 44

Figure S7. Forest plot for the effects of chatbots on self-ambivalence and appearance distress . 44

Figure S8. Forest plot for the effects of chatbots on life satisfaction and well-being 45

Figure S9. Forest plot for the effects of chatbots on self-efficacy 45

Figure S10. Forest plot for the effects of chatbots on health behavior change 45

Figure S11. Leave-one-out sensitivity analysis plots for the effects of chatbots 46

Figure S12. Bubble diagram of the significant result of meta-regression 51

Figure S13. Risk of bias summary 52

Table S1. Search Strategies

PubMed

#	Searches	Results
**1**	"Mental Disorders"[Mesh]	1,513,465
**2**	"Mental Health"[Mesh]	69,577
**3**	"mental disorder*"[Title/Abstract] OR "mental illness*"[Title/Abstract] OR "mental health"[Title/Abstract] OR "psychotic disorder*"[Title/Abstract] OR psycholog*[Title/Abstract] OR anxiety[Title/Abstract] OR "obsessive compulsive disorder*"[Title/Abstract] OR panic[Title/Abstract] OR phobia*[Title/Abstract] OR "phobic disorder*"[Title/Abstract] OR agoraphobia[Title/Abstract] OR "impulse control disorder*"[Title/Abstract] OR "intermittent explosive disorder*"[Title/Abstract] OR "eating disorder*"[Title/Abstract] OR anorexia[Title/Abstract] OR bulimia[Title/Abstract] OR "mood disorder*"[Title/Abstract] OR depress*[Title/Abstract] OR bipolar[Title/Abstract] OR schizophrenia[Title/Abstract] OR "affective disorder*"[Title/Abstract] OR psychosis*[Title/Abstract] OR autism[Title/Abstract] OR "attention deficit hyperactivity disorder*"[Title/Abstract] OR "sleep disorder*"[Title/Abstract] OR Insomnia[Title/Abstract] OR distress[Title/Abstract] OR addiction[Title/Abstract] OR "substance use disorder*"[Title/Abstract] OR "alcohol use disorder*"[Title/Abstract] OR "drug use disorder*"[Title/Abstract] OR trauma*[Title/Abstract] OR "post-traumatic stress disorder*"[Title/Abstract] OR PTSD[Title/Abstract]	2,184,554
**4**	#1 OR #2 OR #3	3,018,819
**5**	"Health Behavior"[Mesh]	374,260
**6**	"Health Risk Behaviors"[Mesh]	1,045
**7**	"health behavior*"[Title/Abstract] OR "health related behavior*"[Title/Abstract] OR "health risk behavior*"[Title/Abstract] OR "health promotion"[Title/Abstract] OR self-care[Title/Abstract] OR self-management[Title/Abstract] OR self-examination*[Title/Abstract] OR "Breast self-examination*"[Title/Abstract] OR "cervical screening"[Title/Abstract] OR "colorectal screening"[Title/Abstract] OR "genetic counsel*"[Title/Abstract] OR "genetic test*"[Title/Abstract] OR "sleep hygiene"[Title/Abstract] OR "sleep habit*"[Title/Abstract] OR "physical activit*"[Title/Abstract] OR exercise[Title/Abstract] OR smoking[Title/Abstract] OR "cigarette use"[Title/Abstract] OR "tobacco use"[Title/Abstract] OR "substance use"[Title/Abstract] OR "alcohol use"[Title/Abstract] OR drinking[Title/Abstract] OR "drug abuse"[Title/Abstract] OR "treatment adherence"[Title/Abstract] OR "patient compliance*"[Title/Abstract] OR "patient acceptance*"[Title/Abstract] OR "healthcare utilization"[Title/Abstract] OR "healthcare acceptabilit*"[Title/Abstract] OR "healthcare seeking behavior*"[Title/Abstract] OR "drug adherence"[Title/Abstract] OR "patient participation"[Title/Abstract] OR "patient involvement"[Title/Abstract] OR "patient engagement"[Title/Abstract] OR "patient satisfaction"[Title/Abstract] OR "treatment refusal*"[Title/Abstract] OR "vaccin* behavior*"[Title/Abstract] OR "vaccin* refusal*"[Title/Abstract] OR "vaccin* hesitanc*"[Title/Abstract] OR "vaccin* delay*"[Title/Abstract]	1,220,448
**8**	#5 OR #6 OR #7	1,469,920
**9**	#4 OR #8	4,166,054
**10**	chatbot*[Title/Abstract] OR "chat bot*"[Title/Abstract] OR chatterbot*[Title/Abstract] OR "chatter bot*"[Title/Abstract] OR "AI chatbot*"[Title/Abstract] OR "Artificial Intelligence chatbot*"[Title/Abstract] OR "social bot*"[Title/Abstract] OR "smart bot*"[Title/Abstract] OR smartbot*[Title/Abstract] OR "dialogue system*"[Title/Abstract] OR	11,921

	"conversational agent*"[Title/Abstract] OR "conversational bot*"[Title/Abstract] OR "conversational system*"[Title/Abstract] OR "conversational interface*"[Title/Abstract] OR "virtual coach*"[Title/Abstract] OR "virtual agent*"[Title/Abstract] OR "embodied agent*"[Title/Abstract] OR avatar*[Title/Abstract] OR "virtual character*"[Title/Abstract] OR "animated character*"[Title/Abstract] OR "virtual human*"[Title/Abstract] OR "virtual assistant*"[Title/Abstract] OR "counseling agent*"[Title/Abstract] OR ChatGPT[Title/Abstract] OR Chat-GPT[Title/Abstract] OR "Generative artificial intelligence"[Title/Abstract] OR gen-AI[Title/Abstract] OR genAI[Title/Abstract] OR "generative AI"[Title/Abstract] OR "large language model"[Title/Abstract] OR LLM[Title/Abstract] OR "generative pretrain* transformer"[Title/Abstract]	
**11**	#9 AND #10	2,461
**12**	"Randomized Controlled Trial"[Publication Type]	625,293
**13**	"RCT"[Title/Abstract] OR "randomized trial*"[Title/Abstract] OR "randomised trial*"[Title/Abstract] OR "controlled trial*"[Title/Abstract] OR "randomized controlled study"[Title/Abstract] OR "randomised controlled study"[Title/Abstract] OR "randomized controlled trail*"[Title/Abstract] OR "randomised controlled trial*"[Title/Abstract]	474,096
**14**	#12 OR #13	919,092
**15**	#11 AND #14	317
**16**	#15 AND ("2014/01/01"[Date - MeSH] : "3000"[Date - MeSH])	302

Cochrane Library

#	**Searches**	**Results**
**1**	MeSH descriptor: [Mental Disorders] explode all trees	108,743
**2**	MeSH descriptor: [Mental Health] explode all trees	3,369
**3**	(mental NEXT disorder* OR mental NEXT illness* OR "mental health" OR psychotic NEXT disorder* OR psycholog* OR anxiety OR obsessive NEXT compulsive NEXT disorder* OR panic OR phobia* OR phobic NEXT disorder* OR agoraphobia OR impulse NEXT control NEXT disorder* OR intermittent NEXT explosive NEXT disorder* OR eating NEXT disorder* OR anorexia OR bulimia OR mood NEXT disorder* OR depress* OR bipolar OR schizophrenia OR affective NEXT disorder* OR psychosis* OR autism OR attention NEXT deficit NEXT hyperactivity NEXT disorder* OR sleep NEXT disorder* OR Insomnia OR distress OR addiction OR substance NEXT use NEXT disorder* OR alcohol NEXT use NEXT disorder* OR drug NEXT use NEXT disorder* OR trauma* OR post-traumatic NEXT stress NEXT disorder* OR PTSD):ti,ab,kw	360,977
**4**	#1 OR #2 OR #3	386,509
**5**	MeSH descriptor: [Health Behavior] explode all trees	48,470
**6**	MeSH descriptor: [Health Risk Behaviors] explode all trees	67
**7**	(health NEXT behavior* OR health NEXT related NEXT behavior* OR health NEXT risk NEXT behavior* OR "health promotion" OR "self care" OR "self management" OR self NEXT examination* OR Breast NEXT self NEXT examination* OR "cervical screening" OR "colorectal screening" OR genetic NEXT counsel* OR genetic NEXT test* OR "sleep hygiene" OR sleep NEXT habit* OR physical NEXT activit* OR exercise OR smoking OR "cigarette use" OR "tobacco use" OR "substance use" OR "alcohol use" OR drinking OR "drug abuse" OR "treatment adherence" OR patient NEXT compliance* OR patient NEXT acceptance* OR "healthcare utilization" OR healthcare NEXT acceptabilit* OR healthcare NEXT seeking NEXT behavior* OR "drug adherence" OR "patient participation" OR "patient involvement" OR "patient engagement" OR "patient satisfaction" OR treatment NEXT refusal* OR	312,551

	vaccin* NEXT behavior* OR vaccin* NEXT refusal* OR vaccin* NEXT hesitanc* OR vaccin* NEXT delay*):ti,ab,kw	
**8**	#5 OR #6 OR #7	316,656
**9**	#4 OR #8	609,570
**10**	(chatbot* OR chat NEXT bot* OR chatterbot* OR chatter NEXT bot* OR AI NEXT chatbot* OR Artificial NEXT Intelligence NEXT chatbot* OR social NEXT bot* OR smart NEXT bot* OR smartbot* OR dialogue NEXT system* OR conversational NEXT agent* OR conversational NEXT bot* OR conversational NEXT system* OR conversational NEXT interface* OR virtual NEXT coach* OR virtual NEXT agent* OR embodied NEXT agent* OR avatar* OR virtual NEXT character* OR animated NEXT character* OR virtual NEXT human* OR virtual NEXT assistant* OR counseling NEXT agent* OR ChatGPT OR "Chat GPT" OR "Generative artificial intelligence" OR "gen AI" OR genAI OR "generative AI" OR "large language model" OR LLM OR generative NEXT pretrain* NEXT transformer):ti,ab,kw	1,252
**11**	#9 AND #10	821
**12**	MeSH descriptor: [Randomized Controlled Trial] explode all trees	37
**13**	(RCT OR randomized NEXT trial* OR randomised NEXT trial* OR controlled NEXT trial* OR "randomized controlled study" OR "randomised controlled study" OR randomized NEXT controlled NEXT trail* OR randomised NEXT controlled NEXT trial*):ti,ab,kw	837,713
**14**	#12 OR #13	837,713
**15**	#11 AND #14 with Publication Year from 2014 to 2024, in Trials (Word variations have been searched)	458

PsycINFO

#	**Searches**	**Results**
**1**	MAINSUBJECT.EXACT.EXPLODE("Mental Disorders")	1,145,216
**2**	MAINSUBJECT.EXACT.EXPLODE("Mental Health")	104,238
**3**	tiab("mental disorder*" OR "mental illness*" OR "mental health" OR "psychotic disorder*" OR psycholog* OR anxiety OR "obsessive compulsive disorder*" OR panic OR phobia* OR "phobic disorder*" OR agoraphobia OR "impulse control disorder*" OR "intermittent explosive disorder*" OR "eating disorder*" OR anorexia OR bulimia OR "mood disorder*" OR depress* OR bipolar OR schizophrenia OR "affective disorder*" OR psychosis* OR autism OR "attention deficit hyperactivity disorder*" OR "sleep disorder*" OR Insomnia OR distress OR addiction OR "substance use disorder*" OR "alcohol use disorder*" OR "drug use disorder*" OR trauma* OR "post-traumatic stress disorder*" OR PTSD)	1,529,332
**4**	#1 OR #2 OR #3	1,996,168
**5**	MAINSUBJECT.EXACT.EXPLODE("Health Behavior")	46,235
**6**	MAINSUBJECT.EXACT.EXPLODE("Health Risk Behavior")	3,736
**7**	tiab("health behavior*" OR "health related behavior*" OR "health risk behavior*" OR "health promotion" OR self-care OR self-management OR self-examination* OR "Breast self-examination*" OR "cervical screening" OR "colorectal screening" OR "genetic counsel*" OR "genetic test*" OR "sleep hygiene" OR "sleep habit*" OR "physical activit*" OR exercise OR smoking OR "cigarette use" OR "tobacco use" OR "substance use" OR "alcohol use" OR drinking OR "drug abuse" OR "treatment adherence" OR "patient compliance*" OR "patient acceptance*" OR "healthcare utilization" OR "healthcare acceptabilit*" OR "healthcare seeking behavior*" OR "drug adherence" OR "patient participation" OR "patient involvement" OR "patient engagement" OR "patient satisfaction" OR "treatment refusal*" OR "vaccin* behavior*" OR "vaccin* refusal*" OR "vaccin* hesitanc*" OR "vaccin* delay*")	336,008
**8**	#5 OR #6 OR #7	358,870

**9**	#4 OR #8	2,182,563
**10**	MAINSUBJECT.EXACT.EXPLODE("Chatbots")	633
**11**	MAINSUBJECT.EXACT.EXPLODE("Large Language Models")	199
**12**	MAINSUBJECT.EXACT.EXPLODE("Conversational Agents")	933
**13**	MAINSUBJECT.EXACT.EXPLODE("Generative Artificial Intelligence")	128
**14**	tiab(chatbot* OR "chat bot*" OR chatterbot* OR "chatter bot*" OR "AI chatbot*" OR "Artificial Intelligence chatbot*" OR "social bot*" OR "smart bot*" OR smartbot* OR "dialogue system*" OR "conversational agent*" OR "conversational bot*" OR "conversational system*" OR "conversational interface*" OR "virtual coach*" OR "virtual agent*" OR "embodied agent*" OR avatar* OR "virtual character*" OR "animated character*" OR "virtual human*" OR "virtual assistant*" OR "counseling agent*" OR ChatGPT OR Chat-GPT OR "Generative artificial intelligence" OR gen-AI OR genAI OR "generative AI" OR "large language model" OR LLM OR "generative pretrain* transformer")	5,031
**15**	#10 OR #11 OR #12 OR #13 OR #14	5,193
**16**	#9 AND #15	1207
**17**	MAINSUBJECT.EXACT.EXPLODE("Randomized Controlled Trials")	1,687
**18**	tiab("RCT" OR "randomized trial*" OR "randomised trial*" OR "controlled trial*" OR "randomized controlled study" OR "randomised controlled study" OR "randomized controlled trail*" OR "randomised controlled trial*")	68,879
**19**	#17 OR #18	69,401
**20**	#16 AND #19	86
**21**	#20 AND pd(20140101-20241026)	82

CINAHL

#	**Searches**	**Results**
**1**	(MH "Mental Disorders+")	675,237
**2**	(MM "Persons with Mental Disorders")	56
**3**	(MM "Mental Health")	35,323
**4**	AB ("mental disorder*" OR "mental illness*" OR "mental health" OR "psychotic disorder*" OR psycholog* OR anxiety OR "obsessive compulsive disorder*" OR panic OR phobia* OR "phobic disorder*" OR agoraphobia OR "impulse control disorder*" OR "intermittent explosive disorder*" OR "eating disorder*" OR anorexia OR bulimia OR "mood disorder*" OR depress* OR bipolar OR schizophrenia OR "affective disorder*" OR psychosis* OR autism OR "attention deficit hyperactivity disorder*" OR "sleep disorder*" OR Insomnia OR distress OR addiction OR "substance use disorder*" OR "alcohol use disorder*" OR "drug use disorder*" OR trauma* OR "post-traumatic stress disorder*" OR PTSD)	602,083
**5**	#1 OR #2 OR #3 OR #4	1,048,727
**6**	(MH "Health Behavior+")	148,168
**7**	(MH "Health Behavior (Iowa NOC)+")	23
**8**	(MH "Health Behavior Component (Saba CCC)+")	37
**9**	(MH "Health Seeking Behaviors (NANDA)+")	22
**10**	(MH "Domain IV: Health-Related Behaviors Domain (Omaha)+")	10
**11**	AB ("health behavior*" OR "health related behavior*" OR "health risk behavior*" OR "health promotion" OR self-care OR self-management OR self-examination* OR "Breast self-examination*" OR "cervical screening" OR "colorectal screening" OR "genetic counsel*" OR "genetic test*" OR "sleep hygiene" OR "sleep habit*" OR "physical activit*" OR exercise OR smoking OR "cigarette use" OR "tobacco use" OR "substance use" OR "alcohol use" OR drinking OR "drug abuse" OR "treatment adherence" OR "patient compliance*" OR "patient acceptance*" OR "healthcare utilization" OR "healthcare acceptabilit*" OR "healthcare seeking behavior*" OR "drug adherence" OR	395,603

	"patient participation" OR "patient involvement" OR "patient engagement" OR "patient satisfaction" OR "treatment refusal*" OR "vaccin* behavior*" OR "vaccin* refusal*" OR "vaccin* hesitanc*" OR "vaccin* delay*")	
**12**	#6 OR #7 OR #8 OR #9 OR #10 OR #11	497,783
**13**	#5 OR #12	1,388,458
**14**	AB (chatbot* OR "chat bot*" OR chatterbot* OR "chatter bot*" OR "AI chatbot*" OR "Artificial Intelligence chatbot*" OR "social bot*" OR "smart bot*" OR smartbot* OR "dialogue system*" OR "conversational agent*" OR "conversational bot*" OR "conversational system*" OR "conversational interface*" OR "virtual coach*" OR "virtual agent*" OR "embodied agent*" OR avatar* OR "virtual character*" OR "animated character*" OR "virtual human*" OR "virtual assistant*" OR "counseling agent*" OR ChatGPT OR Chat-GPT OR "Generative artificial intelligence" OR gen-AI OR genAI OR "generative AI" OR "large language model" OR LLM OR "generative pretrain* transformer")	1,936
**15**	#13 AND #14	618
**16**	(MH "Randomized Controlled Trials+")	148,010
**17**	AB ("RCT" OR "randomized trial*" OR "randomised trial*" OR "controlled trial*" OR "randomized controlled study" OR "randomised controlled study" OR "randomized controlled trail*" OR "randomised controlled trial*")	142,126
**18**	#16 OR #17	249,421
**19**	#15 AND #18	89
**20**	#19 Limiters - Publication Date: 20140101-20241231	82

Embase

#	**Searches**	**Results**
**1**	'mental disease'/exp OR 'abnormal mental state' OR 'disease, mental' OR 'diseased mental state' OR 'disorder, mental' OR 'disordered mental state' OR 'disturbed mental state' OR 'illness, mental' OR 'insanity' OR 'mental abnormality' OR 'mental change' OR 'mental confusion' OR 'mental defect' OR 'mental disease' OR 'mental disorder' OR 'mental disorders' OR 'mental disorders diagnosed in childhood' OR 'mental disturbance' OR 'mental health condition' OR 'mental health disease' OR 'mental health disorder' OR 'mental health issue' OR 'mental health problem' OR 'mental illness' OR 'mental insufficiency' OR 'mental symptom' OR 'mentally ill' OR 'neurodevelopmental disorder' OR 'neurodevelopmental disorders' OR 'neuropsychiatric disease' OR 'neuropsychiatric diseases' OR 'neuropsychiatric disorder' OR 'neuropsychiatric disorders' OR 'psychiatric disease' OR 'psychiatric disorder' OR 'psychiatric illness' OR 'psychiatric symptom' OR 'psychic disease' OR 'psychic disorder' OR 'psychic disturbance' OR 'psychologic disorder' OR 'psychologic disturbance' OR 'psychological disorder' OR 'psychological disturbance' OR 'psychopathology'	3,113,202
**2**	'mental health'/exp OR 'condition, mental' OR 'health, mental' OR 'mental care' OR 'mental condition' OR 'mental factor' OR 'mental health' OR 'mental help' OR 'mental service' OR 'mental state' OR 'mental status' OR 'mental status schedule' OR 'psychic health'	772,994
**3**	'mental disorder*':ti,ab,kw OR 'mental illness*':ti,ab,kw OR 'mental health':ti,ab,kw OR 'psychotic disorder*':ti,ab,kw OR psycholog*:ti,ab,kw OR anxiety:ti,ab,kw OR 'obsessive compulsive disorder*':ti,ab,kw OR panic:ti,ab,kw OR phobia*:ti,ab,kw OR 'phobic disorder*':ti,ab,kw OR agoraphobia:ti,ab,kw OR 'impulse control disorder*':ti,ab,kw OR 'intermittent explosive disorder*':ti,ab,kw OR 'eating disorder*':ti,ab,kw OR anorexia:ti,ab,kw OR bulimia:ti,ab,kw OR 'mood disorder*':ti,ab,kw OR depress*:ti,ab,kw OR bipolar:ti,ab,kw OR schizophrenia:ti,ab,kw OR 'affective disorder*':ti,ab,kw OR psychosis*:ti,ab,kw OR autism:ti,ab,kw OR 'attention	2,911,412

	deficit hyperactivity disorder*':ti,ab,kw OR 'sleep disorder*':ti,ab,kw OR insomnia:ti,ab,kw OR distress:ti,ab,kw OR addiction:ti,ab,kw OR 'substance use disorder*':ti,ab,kw OR 'alcohol use disorder*':ti,ab,kw OR 'drug use disorder*':ti,ab,kw OR trauma*:ti,ab,kw OR 'post-traumatic stress disorder*':ti,ab,kw OR ptsd:ti,ab,kw	
**4**	#1 OR #2 OR #3	4,752,673
**5**	'health behavior'/exp OR 'behavior, health' OR 'behaviour, health' OR 'health behavior' OR 'health behaviour' OR 'health promoting behavior' OR 'health promoting behaviour' OR 'health related behavior' OR 'health related behaviour'	553,868
**6**	'high risk behavior'/exp OR 'health risk behavior' OR 'health risk behaviors' OR 'health risk behaviour' OR 'health risk behaviours' OR 'high risk behavior' OR 'high risk behaviour' OR 'risk taking' OR 'risk taking behavior' OR 'risk taking behaviour' OR 'risk-taking' OR 'risky behavior' OR 'risky behaviour'	42,315
**7**	'health behavior*':ti,ab,kw OR 'health related behavior*':ti,ab,kw OR 'health risk behavior*':ti,ab,kw OR 'health promotion':ti,ab,kw OR 'self care':ti,ab,kw OR 'self management':ti,ab,kw OR 'self examination*':ti,ab,kw OR 'breast self-examination*':ti,ab,kw OR 'cervical screening':ti,ab,kw OR 'colorectal screening':ti,ab,kw OR 'genetic counsel*':ti,ab,kw OR 'genetic test*':ti,ab,kw OR 'sleep hygiene':ti,ab,kw OR 'sleep habit*':ti,ab,kw OR 'physical activit*':ti,ab,kw OR exercise:ti,ab,kw OR smoking:ti,ab,kw OR 'cigarette use':ti,ab,kw OR 'tobacco use':ti,ab,kw OR 'substance use':ti,ab,kw OR 'alcohol use':ti,ab,kw OR drinking:ti,ab,kw OR 'drug abuse':ti,ab,kw OR 'treatment adherence':ti,ab,kw OR 'patient compliance*':ti,ab,kw OR 'patient acceptance*':ti,ab,kw OR 'healthcare utilization':ti,ab,kw OR 'healthcare acceptabilit*':ti,ab,kw OR 'healthcare seeking behavior*':ti,ab,kw OR 'drug adherence':ti,ab,kw OR 'patient participation':ti,ab,kw OR 'patient involvement':ti,ab,kw OR 'patient engagement':ti,ab,kw OR 'patient satisfaction':ti,ab,kw OR 'treatment refusal*':ti,ab,kw OR 'vaccin* behavior*':ti,ab,kw OR 'vaccin* refusal*':ti,ab,kw OR 'vaccin* hesitanc*':ti,ab,kw OR 'vaccin* delay*':ti,ab,kw	1,700,495
**8**	#5 OR #6 OR #7	2,063,950
**9**	'chatbot'/exp OR 'automated conversational agent' OR 'chatbot' OR 'conversational agent' OR 'embodied conversational agent' OR 'online assistant' OR 'smart conversational agent'	5,617
**10**	'chatgpt'/exp OR 'chat-gpt' OR 'chatgpt'	5,178
**11**	'large language model'/exp OR 'large language model'	5,674
**12**	'generative artificial intelligence'/exp OR 'creative ai' OR 'creative artificial intelligence' OR 'gen-ai' OR 'genai' OR 'generative a.i.' OR 'generative ai' OR 'generative artificial intelligence'	5,326
**13**	chatbot*:ti,ab,kw OR 'chat bot*':ti,ab,kw OR chatterbot*:ti,ab,kw OR 'chatter bot*':ti,ab,kw OR 'ai chatbot*':ti,ab,kw OR 'artificial intelligence chatbot*':ti,ab,kw OR 'social bot*':ti,ab,kw OR 'smart bot*':ti,ab,kw OR smartbot*:ti,ab,kw OR 'dialogue system*':ti,ab,kw OR 'conversational agent*':ti,ab,kw OR 'conversational bot*':ti,ab,kw OR 'conversational system*':ti,ab,kw OR 'conversational interface*':ti,ab,kw OR 'virtual coach*':ti,ab,kw OR 'virtual agent*':ti,ab,kw OR 'embodied agent*':ti,ab,kw OR avatar*:ti,ab,kw OR 'virtual character*':ti,ab,kw OR 'animated character*':ti,ab,kw OR 'virtual human*':ti,ab,kw OR 'virtual assistant*':ti,ab,kw OR 'counseling agent*':ti,ab,kw OR chatgpt:ti,ab,kw OR 'chat gpt':ti,ab,kw OR 'generative artificial intelligence':ti,ab,kw OR 'gen ai':ti,ab,kw OR genai:ti,ab,kw OR 'generative ai':ti,ab,kw OR 'large language model':ti,ab,kw OR llm:ti,ab,kw OR 'generative pretrain* transformer':ti,ab,kw	13,023
**14**	#9 OR #10 OR #11 OR #12 OR #13	14,970
**15**	'randomized controlled trial'/exp OR 'controlled trial, randomized' OR 'randomised controlled study' OR 'randomised controlled trial' OR 'randomized controlled study' OR 'randomized controlled trial' OR 'trial, randomized controlled'	1,157,722

**16**	rct:ti,ab,kw OR 'randomized trial*':ti,ab,kw OR 'randomised trial*':ti,ab,kw OR 'controlled trial*':ti,ab,kw	634,360
**17**	#15 OR #16	1,351,481
**18**	#4 OR #8	6,321,277
**19**	#14 AND #18	3,590
**20**	#17 AND #19	444
**21**	#20 AND [2014-2024]/py	424

Web of Science

#	Searches	Results
**1**	TS=("mental disorder*" OR "mental illness*" OR "mental health" OR "psychotic disorder*" OR psycholog* OR anxiety OR "obsessive compulsive disorder*" OR panic OR phobia* OR "phobic disorder*" OR agoraphobia OR "impulse control disorder*" OR "intermittent explosive disorder*" OR "eating disorder*" OR anorexia OR bulimia OR "mood disorder*" OR depress* OR bipolar OR schizophrenia OR "affective disorder*" OR psychosis* OR autism OR "attention deficit hyperactivity disorder*" OR "sleep disorder*" OR Insomnia OR distress OR addiction OR "substance use disorder*" OR "alcohol use disorder*" OR "drug use disorder*" OR trauma* OR "post-traumatic stress disorder*" OR PTSD)	3,060,219
**2**	TS=("health behavior*" OR "health related behavior*" OR "health risk behavior*" OR "health promotion" OR self-care OR self-management OR self-examination* OR "Breast self-examination*" OR "cervical screening" OR "colorectal screening" OR "genetic counsel*" OR "genetic test*" OR "sleep hygiene" OR "sleep habit*" OR "physical activit*" OR exercise OR smoking OR "cigarette use" OR "tobacco use" OR "substance use" OR "alcohol use" OR drinking OR "drug abuse" OR "treatment adherence" OR "patient compliance*" OR "patient acceptance*" OR "healthcare utilization" OR "healthcare acceptabilit*" OR "healthcare seeking behavior*" OR "drug adherence" OR "patient participation" OR "patient involvement" OR "patient engagement" OR "patient satisfaction" OR "treatment refusal*" OR "vaccin* behavior*" OR "vaccin* refusal*" OR "vaccin* hesitanc*" OR "vaccin* delay*")	1,822,492
**3**	#1 OR #2	4,585,021
**4**	TS=(chatbot* OR "chat bot*" OR chatterbot* OR "chatter bot*" OR "AI chatbot*" OR "Artificial Intelligence chatbot*" OR "social bot*" OR "smart bot*" OR smartbot* OR "dialogue system*" OR "conversational agent*" OR "conversational bot*" OR "conversational system*" OR "conversational interface*" OR "virtual coach*" OR "virtual agent*" OR "embodied agent*" OR avatar* OR "virtual character*" OR "animated character*" OR "virtual human*" OR "virtual assistant*" OR "counseling agent*" OR ChatGPT OR Chat-GPT OR "Generative artificial intelligence" OR gen-AI OR genAI OR "generative AI" OR "large language model" OR LLM OR "generative pretrain* transformer")	45,613
**5**	#3 AND #4	5,400
**6**	TS=("RCT" OR "randomized trial*" OR "randomised trial*" OR "controlled trial*" OR "randomized controlled study" OR "randomised controlled study" OR "randomized controlled trail*" OR "randomised controlled trial*")	717,346
**7**	#5 AND #6	334
**8**	#7 Timespan: 2014-01-01 to 2024-10-26	319

Scopus

#	Searches	Results
**1**	TITLE-ABS-KEY ("mental disorder*" OR "mental illness*" OR "mental health" OR "psychotic disorder*" OR psycholog* OR anxiety OR "obsessive compulsive disorder*" OR panic OR phobia* OR "phobic disorder*" OR agoraphobia OR "impulse control disorder*" OR "intermittent explosive disorder*" OR "eating disorder*" OR anorexia OR bulimia OR "mood disorder*" OR depress* OR bipolar OR schizophrenia OR "affective disorder*" OR psychosis* OR autism OR "attention deficit hyperactivity disorder*" OR "sleep disorder*" OR insomnia OR distress OR addiction OR "substance use disorder*" OR "alcohol use disorder*" OR "drug use disorder*" OR trauma* OR "post-traumatic stress disorder*" OR ptsd)	5,170,111
**2**	TITLE-ABS-KEY ("health behavior*" OR "health related behavior*" OR "health risk behavior*" OR "health promotion" OR self-care OR self-management OR self-examination* OR "Breast self-examination*" OR "cervical screening" OR "colorectal screening" OR "genetic counsel*" OR "genetic test*" OR "sleep hygiene" OR "sleep habit*" OR "physical activit*" OR exercise OR smoking OR "cigarette use" OR "tobacco use" OR "substance use" OR "alcohol use" OR drinking OR "drug abuse" OR "treatment adherence" OR "patient compliance*" OR "patient acceptance*" OR "healthcare utilization" OR "healthcare acceptabilit*" OR "healthcare seeking behavior*" OR "drug adherence" OR "patient participation" OR "patient involvement" OR "patient engagement" OR "patient satisfaction" OR "treatment refusal*" OR "vaccin* behavior*" OR "vaccin* refusal*" OR "vaccin* hesitanc*" OR "vaccin* delay*")	2,789,522
**3**	#1 OR #2	7,319,211
**4**	TITLE-ABS-KEY (chatbot* OR "chat bot*" OR chatterbot* OR "chatter bot*" OR "AI chatbot*" OR "Artificial Intelligence chatbot*" OR "social bot*" OR "smart bot*" OR smartbot* OR "dialogue system*" OR "conversational agent*" OR "conversational bot*" OR "conversational system*" OR "conversational interface*" OR "virtual coach*" OR "virtual agent*" OR "embodied agent*" OR avatar* OR "virtual character*" OR "animated character*" OR "virtual human*" OR "virtual assistant*" OR "counseling agent*" OR chatgpt OR chat-gpt OR "Generative artificial intelligence" OR gen-ai OR genai OR "generative AI" OR "large language model" OR llm OR "generative pretrain* transformer")	84,784
**5**	#3 AND #4	8,717
**6**	TITLE-ABS-KEY ("RCT" OR "randomized trial*" OR "randomised trial*" OR "controlled trial*" OR "randomized controlled study" OR "randomised controlled study" OR "randomized controlled trail*" OR "randomised controlled trial*")	1,169,714
**7**	#5 AND #6	439
**8**	#7 AND PUBYEAR > 2013 AND PUBYEAR < 2025	415

IEEE Xplore

#	Searches	Results
**1**	("All Metadata":"mental disorders" OR "All Metadata":"mental health" OR "All Metadata":psycho* OR "All Metadata":anxiety OR "All Metadata":panic OR "All Metadata":phobia OR "All Metadata":"mood disorders" OR "All Metadata":depress* OR "All Metadata":distress OR "All Metadata":addiction OR "All Metadata":trauma* OR "All Metadata":PTSD)	77,055
**2**	("All Metadata":"health behaviors" OR "All Metadata":"health related behaviors" OR "All Metadata":"health risk behaviors" OR "All Metadata":"health promotion" OR "All Metadata":self-care OR "All	62,800

	Metadata":self-management OR "All Metadata":self-examination OR "All Metadata":"physical activit*" OR "All Metadata":exercise OR "All Metadata":smoking OR "All Metadata":"substance use" OR "All Metadata":drinking OR "All Metadata":sleep)	
**3**	#1 OR #2	135941
**4**	("All Metadata":chatbot* OR "All Metadata":chatterbot* OR "All Metadata":"AI chatbot" OR "All Metadata":"dialogue system*" OR "All Metadata":"conversational agent*" OR "All Metadata":"Generative artificial intelligence" OR "All Metadata":"large language model")	10500
**5**	#3 AND #4	1032
**6**	("All Metadata":"RCT" OR "All Metadata":randomized controlled trial)	857
**7**	#5 AND #6	237
**8**	#7 Year: 2014-2024	210

Table S2. Summary of characteristics of included studies

Author, Year	Region	Population	Sample type	Recruitment setting	Sample size	Age range/ Mean (SD)	Sex (female)	Intervention	Control	Intervention duration	Follow-up	Primary Outcomes (Measures)	Mediator	Moderator	Findings
Bricker, 2024[16]	USA	Daily smoker	Clinical	Online	404	36.00 (10.40)	70.0%	QuitBot (n=190)	SmokefreeTXT (group 1, n=144); Wait-list (group 2, n=54)	42 days	at 3 months	30-day PPA, 7-day PPA	NR	NR	The quit rate in the QuitBot group were significantly higher than the delayed group (*p*=.002).
Chan, 2024[51]	Hong Kong, China	College students and staffs screened with significant insomnia	Subclinical	Community	129	34.09 (12.05)	76.0%	dCBTi-chatbot in Sleep Sensei (n=26)	dCBTi without any coaching (group 1, n=21); digital sleep hygiene and self-monitoring (group 2, n=30)	6 weeks	at 4 weeks	Psychological well-being (SWLS), insomnia severity (ISI), sleep-related safety behaviors (SRBQ), sleep condition (SCI), daytime sleepiness (ESS), depression (PHQ-9), anxiety (GAD-7), fatigue (FAS), endorsement of dysfunctional thoughts and beliefs (DBAS-16)	NR	NR	Significant differences between dCBTi-chatbot group and dSH group in DBAS-16 score (*p*=.02) and sleep condition indicator (*p*=.04).
Lee, 2023[14]	Taiwan, China	Outpatients diagnosed with methamphetamine use disorder	Clinical	Clinical	99	37.00 (10.40)	18.2%	Coach Xiaonan (n=50)	Standard treatment (n=49)	2 months	at 6 months	Monthly urine test, the readiness to change (URICA)	NR	NR	The experimental group had fewer MA-positive urine samples than the control group (*p*=.003);<br>No significant differences in scores for readiness to change between two groups;<br>The proportion of MA-positive urine samples was negatively correlated with readiness to change (*p*=.001).
De Filippo, 2023[36]	South Africa	Young women who potentially experienced IPV	Subclinical	Online	19643	21.2	100.0%	Chatbot for attention treatment (group 1, n=3930) gamified chatbot (group 2, n=5891), narrative-	Assessment-only (n=3930)	3 months	No follow-up	Gender attitudes (aGRS), IPV exposure (aWHO), Identification of unhealthy relationship behaviors (aVAS), depressive symptoms (PHQ-2)	NR	NR	All three chatbots improved beliefs in power equity in relationships compared to the pure control;<br>Women in the Gamified arm reported lower rates of IPV exposure (55%) than those in

								based chatbot (group 3, n=5893)							the pure control group (62%); Both gamified chatbot and narrative-based chatbot increased users' ability to correctly identify unhealthy relationship behaviors compared to the Attention chatbot group; Gamified chatbot was slightly protective for depressive symptoms, but this was not statistically significant.
Drouin, 2022[52]	USA	College students	Nonclinical	Community	417	19.8 (3.41)	71.2%	Replika (n=133)	F2F (group 1, n=63 dyads); instant messaging (group 2, n=78 dyads)	20 min	No follow-up	Positive and negative affect (PANAS)	NR	NR	No significant group differences in positive emotions; Participants in the F2F condition reported slightly (but significantly) more negative emotions than those who chatted with the chatbot.
Ehrlich, 2024[40]	UK	The first-year undergraduate students	Nonclinical	Community	Trial 1, 177; Trial 2, 250	Trial 1, 20.80 (5.18); Trial 2, 20.23 (4.96)	Trial 1, 62%; Trial 2, 58%	Mind Tutor (trial 1, n=92; trial 2, n=125)	Wait-list (trial 1, n=85; trial 2, n=125)	Trial 1, 6 weeks; Trial 2, 8 weeks	No follow-up	Well-being (SWEMWBS), Satisfaction with life (SWLS), Positive and negative affect (PANAS-SF), Mindfulness (CAMS-R), Self-efficacy (the General Self-Efficacy Scale)	NR	NR	In both trials, there were no significant differences in outcomes between groups.
Fitzpatrick, 2017[17]	USA	College students with depression and anxiety symptoms	Subclinical	Community	70	22.2(2.33)	67.14%	Woebot (n=34)	eBook (n=36)	2 weeks	No follow-up	Depression (PHQ-9), Anxiety (GAD-7), Positive and negative Affect (PANAS)	NR	NR	Decreased depression in chatbot group and significant difference between groups (*p*=.017); No other significant between-group differences in anxiety or affect.

Fitzsimmons-Craft, 2022[43]	USA	Young women screened as high risk for eating disorders (EDs)	Subclinical	Online	700	21.8(3.09)	100.0%	Tessa (n=352)	Wait-list (n=348)	1 month	at 3, 6 months	Weight and shape concerns (WCS), Internalization (SATAQ-4R), eating disorder (EDE-Q), depression (PHQ-8), anxiety (GAD-7)	NR	NR	Greater decrease in WCS in chatbot group than control from baseline to both 3- and 6-month follow-up; Significant reduction in thin-ideal internalization in both groups at 3 months; Greater reduction in global ED psychopathology in chatbot group than control from baseline to 3-month follow-up, and both groups showed significant reductions from baseline to 6-month follow-up; Significant decrease in depression in both groups over both periods; Significant reduction in both groups from baseline to 3-month follow-up; The odds of remining nonclinical at both follow-ups were significantly higher in chatbot group compared to the control (OR(3-month) =2.37[1.37-4.11]; OR(6-month) =2.13[1.26-3.59].
Fulmer, 2018[15]	USA	College students	Nonclinical	Mixed (Online, Community)	75	22.93 (4.17)	70.3%	Tess (group 1, n=24; group 2, n=26)	eBook (n=25)	Tess group 1, 2 weeks; Tess group 2, 4 weeks	No follow-up	Depression (PHQ-9), Anxiety (GAD-7), Positive and negative affect (PANAS)	NR	NR	Significant difference in change of depression between the control group and group 1 (*p*=.027); Significant difference in change of anxiety between the control group and both group 1 (*p*=.045) and 2 (*p*=.021); Significant difference in PANAS changes

															between the control group and Tess group 1 (*p*=.028).
Greer, 2019[39]	USA	Young cancer survivors	Clinical	Mixed (Online, Community)	45	25(2.9)	80.0%	Vivibot (n=25)	Wait-list (n=20)	4 weeks	at 8 weeks	Anxiety and depression symptoms (PROMIS), Positive and Negative emotion (DES)	NR	User engagement	Greater reduction in anxiety in chatbot group than the control over 4 weeks (*p*=.09), a trend-level relationship for group by engaged sessions observed for anxiety (*p*=.06); Slight decreases in depressive symptom in two groups with no significant interaction by time (*p*=.77); Similar magnitude decrease in negative emotion in two groups (*p*=0.97).
Haug, 2022[50]	Switzerland	Adolescents from vocational schools	Nonclinical	Community	1351	17.3 (3.0)	43.4%	Ready4life (n=688)	Assessment-only (n=663)	4 months	at 6 months	At risk-drinking, tobacco/e-cigarette use, cannabis use, problematic Internet use (CIUS-5) in the preceding 30 days; General self-efficacy (the Short Scale for Measuring General Self Efficacy Beliefs); Self-perceived stress (a single-item)	NR	NR	A stronger decrease of addictive behaviors in the chatbot group compared to the control (*p*<0.01); Significant group effects for at-risk drinking (*p*<0.01), tobacco/e-cigarette use (*p*=0.03), problematic internet use in the past 30 days (*p*<0.01), and self-perceived stress (*p*<0.01).
He, 2022[45]	China	College students screened with depressive symptoms	Subclinical	Mixed (Online, Community)	148	18.78 (0.89)	37.2%	XiaoE (n=49)	eBook (n=49)	1 week	at 1 month	Depression (PHQ-9)	NR	NR	Significant differences with XiaoE versus eBook in reducing depression at posttest (*p*=.04) and follow-up (*p*=.049).
Hunt, 2021[47]	USA	Adults previously diagnosed with IBS	Clinical	Online	121	32 (10.2)	75.2%	Zemedy (n=62)	Wait-list(n=59)	8 weeks	at 3-month	Gastrointestinal Symptom (GSRSIBS), the Gastrointestinal cognitions (GI-COG), visceral sensitivity (VSI), fear of food (FFQ), depression, anxiety,	Visceral anxiety, catastrophizing, fear of food	Diagnosis of IBS, dosage	Greater improvement for the chatbot group than the control for GI symptom severity (*p*<.001), quality of life (*p*<.001), catastrophizing (*p*<.001), visceral

												and stress (DASS-21), depression (PHQ-9), Quality of life (IBS-QOL)			anxiety (*p*<.001), fear of food (*p*=.001), depression (*p*=.002), and stress (*p*=.04); Visceral anxiety, catastrophizing, and fear of food were all significantly mediate reduction in GI symptom severity (*p*<.001) and IBS-QOL (*p*<.001); Participants who reported meeting Rome IV criteria for IBS at baseline than those who did not showed more improvement for both GI symptoms (*p*=.04) and HRQL (*p*<.001); Dosage was marginally correlated with improvement in quality of life (*p*=.07) and depression (*p*=.08).
Jang, 2021[48]	Korea	Adults with significant attention problems	Clinical	Clinical	46	25.1(7.5)	56.5%	Todaki (n=23)	Book (n=23)	4 weeks	No follow-up	ADHD (CAARS), Depression (QIDS-SR), Anxiety (SAS), Stress (PSS)	NR	User engagement	Significantly greater reduction in DSM-IV hyperactive-impulsive symptoms (*p*=.04) and DSM-IV ADHD symptoms total *p*=.013) in the chatbot group than control; Positive correlation between the number of times of engaging in psychoeducation session and the reduction in DSM-IV ADHD symptoms total scale score (*p*=.03).
Karkosz, 2024[49]	Poland	Young adults screened with mild depressive	Subclinical	Online	81	25.68 (4.56)	71.6%	Fido (n=40)	Book (n=41)	2 weeks	at 1 month	Depression (CESD-R, PHQ-9), worry tendency (PSWQ), anxiety (STAI), positive and negative affect	NR	User engagement	Significant decrease in depressive symptoms, anxiety, worry tendency, negative affect, and increase in

		or anxiety symptoms										(PANAS), satisfaction with life (SWLS), loneliness (R-UCLA)			satisfaction with life and positive affect in both groups (*p*<.001); Negative relationship between loneliness scores and the frequency of chatbot use (*p*=.006).
Klos, 2021[53]	Argentina	College students	Nonclinical	Community	181	18-33	87.2%	Tess (n=99)	eBook (n=82)	8 weeks	No follow-up	Depression (PHQ-9), anxiety (GAD-7)	NR	NR	Statistically significant decrease in anxiety in chatbot group (*p*=.04).
Lavelle, 2021[54]	Ireland	General population	Nonclinical	Mixed (Online, Community)	223	28.01 (10.29)	78.5%	Defusion chatbot (group 1, n=72); restructuring chatbot (group 2, n=80)	Assessment-only (n=71)	5 days	No follow-up	Negative self-referential thoughts (The Target Thought Measure), psychological inflexibility (AAQ-II), cognitive fusion (CFQ-7), Positive and Negative affect (PANAS)	NR	NR	Significant decreases in positive affect between the restructuring group and the control group (*p*=.037).
Liu, 2022[21]	China	College students screened with depression	Subclinical	Community	83	23.08 (1.76)	55.4%	XiaoNan (n=41)	Book (n=42)	16 weeks	at 4, 8, 12, 16 weeks	Depression (PHQ-9), Anxiety (GAD-7), Positive affect (PANAS), positive and Negative affect (PANAS)	NR	NR	Greater decrease in depression (*p*<.01) and anxiety (*p*=.02) in chatbot group compared to bibliotherapy.
Ly, 2017[55]	Sweden	General population	Nonclinical	Mixed (Online, Community)	28	26.2 (7.2)	53.6%	Shim (n=14)	Wait-list (n=14)	2 weeks	No follow-up	Psychological well-being (FS), Stress (PSS-10), Satisfaction with life (SWLS)	NR	NR	Greater improvement in FS (*p*=.032) and decrease in PSS-10 (*p*=.048) in the intervention group than control in completer analysis.
Maeda, 2020[56]	Japan	Young women hoping to have children now or in the future	Nonclinical	Online	927	28.77 (3.57)	100.0%	Fertility education chatbot (n=309)	A *pdf.* about fertility and pre-conception health (group 1, n=309); pdf about the national pension (group 2, n=309)	1 hour	No follow-up	Fertility Knowledge (CFKS-J); Anxiety (STAI); Intention to change pre-conception behaviors	NR	NR	The chatbot group showed significantly lower anxiety than control group 1 and 2 (*p*<.001); Higher increase of intentions to take folic acid (*p*<.001), to receive HPV vaccination (*p*<.001), to obtain a primary obstetrican or gynaecologist (*p*=.005), to take oral contraceptives (*p*<.001) and to try

															to get pregnant (p=.02) in the intervention group than control group 2; Higher increase of the intention to take folic acid (*p*=.003) but lower for the intention to take oral contraceptives (*p*=.005) in the intervention group than control group 1.
Matheson, 2023[44]	Brazil	Young adults with body image and mood concerns	Subclinical	Online	1715	13-18	52.5%	Topity (n=858)	Assessment-only (n=857)	72 hours	at 2, 6 weeks	Body satisfaction (a single 11-point scale), body esteem (Body Esteem Scale for Adolescents and young adults Brazil), state affect (a single 11-point scale), positive and negative affect (PANAS), self-efficacy in addressing body image concerns (The Body Image Self-Efficacy Scale)	NR	Baseline severity	The chatbot group revealed higher levels of body esteem (*p*<.05) than control group; Those with lower body esteem, positive affect, and body image self-efficacy experienced greater intervention benefits than those with higher levels on these variables (*p*<0.001).
Nicol, 2022[57]	USA	Adolescents diagnosed with depression and anxiety	Clinical	Clinical	18	14.7 (1.7)	88.0%	Woebot (n=10)	Wait-list (n=8)	4 weeks	at 8, 12 weeks	Depression (PHQ-A), anxiety (GAD-7), mental health self-efficacy (MHSES)	NR	NR	The chatbot group showed greater improvement on depression than control group over 4 weeks.
Romanovskyi, 2021[46]	Ukraine	College students with low mood, depression, and anxiety symptoms	Subclinical	Community	82	20.85 (0.97)	47.6%	Elomia (n=42)	Self-help guide (n=40)	4 weeks	No follow-up	Depression (PHQ-9), anxiety (GAD-7), positive and negative affect (PANAS)	NR	NR	The chatbot group showed a significant decrease in anxiety, depression, negative affect and increase in positive affect (*p*<.001).
Sabour, 2023[58]	China	General population	Nonclinical	Online	247	30.9 (7.92)	76.9%	ES-bot (group 1, n=90), CBT-bot (group 2, n=90)	Wait-list (n=121)	3 weeks	at 1 month	Depression (PHQ-9), anxiety (GAD-7), positive and negative affect (PANAS), Insomnia severity (ISI)	NR	NR	Significant group × time interaction effects on depression (*p*=.002), negative affect (*p*=.03), and insomnia (*p*=.026).
Schillings,	Germany	Individuals with medium to	Subclinical	Mixed (Online, Community)	118	33.12 (12.87)	76.5%	ELME (n=59)	TAU (n=59)	3 weeks	at 6 weeks	Stress (PSS-10), mindfulness (The 14-item short	NR	NR	Significant time × group interaction

2024[37]		high stress levels										version of the Freiburg Mindfulness Inventory), interoceptive sensibility (IAS), well-being (the 5-item WHO well-being index), emotion regulation (the German version of the Emotion Regulation questionnaire)			effect for mindfulness and emotion regulation reappraisal sub-facet (*p*<.001).
Selaskowski, 2023[59]	Germany	Outpatients diagnosed with ADHD	Clinical	Mixed (Online, Clinical)	40	29.6 (8.4)	52.9%	Chatbot (n=20)	Conventional psychoeducational app (n=20)	3 weeks	No follow-up	ADHD (IDA-R, ADHS-SB), quality of life (WHOQOL), depression and anxiety (DASS-21), verbal intelligence (MWT-B)	NR	NR	No significant group effect or group × time interaction effect observed in AHDH symptom severity. Chatbot group showed significantly higher stress symptoms (*p*=.02) and lower quality of life (*p*=.047) compared to control group.
Stewart, 2023[38]	USA	People who had concerns about COVID-19	Nonclinical	Online	200	Median: 32 (intervention), 29.5 (control)	48.3%	Expressive interviewing agent (n=100)	Assessment-only (n=100)	at least 15 min	at 2 weeks	COVID-19 mental behavior, COVID-19 awareness, social activity, social discussions, Mental health	NR	Writing behavior; ethnicity	A significant group difference (*p*=.03) and ethnicity × group interaction effect were observed on social activity outcome; Using more "anxiety"-related words and fewer "COVID-19"-related words was correlated with a decrease in reported stress (*p*=.006); Using more "COVID-19"-related words and more positive emotion words was correlated with a more personal and more meaningful experience (*p*<.001). Participants who reported higher life satisfaction before

														the session also used fewer "anxiety" and "sad"-related words in their responses (*p*=.004).
Suharwardy, 2023[60]	USA	Women within 72 hours of their delivery	Clinical	Clinical	192	34	100.0%	Woebot (n=96)	TAU (n=96)	2, 4, 6 weeks	No follow-up	Depression and anxiety (EPDS), depression (PHQ-9), anxiety (GAD-7)	NR	NR	Greater decrease in depression in chatbot group than control group (*p*=.025)
Ulrich (BalanceUP), 2024[25]	Switzerland, Germany, Austria	Adults with frequent headaches	Subclinical	Online	198	38.7 (12.14)	86.9%	BalanceUP (n=110)	Wait-list (n=88)	24-60 days	No follow-up	Mental well-being (PHQ-ADS), depression (PHQ-9), anxiety (GAD-7), Somatic symptoms (PHQ-15), Stress (PSS-10), Headache management self-efficacy (HMSE-G-SF), Intention to change behavior, Absenteesim and presenteeism (the Migraine Disability Assessment), pain coping strategies (the Questionnaire for assessment of pain processing), application of BCT	NR	Time	Significant time ×group interaction effect for mental well-being (*p*<.001), depression (*p*<.001), anxiety (*p*=.007), somatic symptoms (*p*<.001), stress (*p*<.003), headache management self-efficacy (*p*<.001), application of BCT (*p*<.001), absenteeism and presenteeism (*p*=.003), cognitive pain coping (*p*<.001), behavioral pain coping (*p*<.001); the reported commitment to change behavior significantly increased with time (*p*<.001)
Ulrich (MISHA), 2024[41]	Switzerland	College students	Nonclinical	Community	140	26.71 (6.29)	73.6%	MISHA (n=70)	Wait-list (n=70)	54 days	No follow-up	Stress (PSS-10), depression (PHQ-9), anxiety (GAD-7), psychosomatic symptoms (PHQ-15), stress management activities/active coping (a rating scale ranging from 1 to 4), subjective stress expertise and goal achievement (two scales ranging from 1 to 10); self-efficacy expectancy (the General self-efficacy scale)	NR	NR	Significantly greater decrease in stress (*p*<.001), depressive symptoms (*p*=.003), and psychosomatic symptoms (*p*=.003) in chatbot group compared to control group.

Vereschagin, 2024[42]	Canada	College students	Nonclinical	Community	1489	Median: 20	70.3%	Minder (n=743)	wait-list (n=746)	30 days	No follow-up	Anxiety (GAD-7), depression (PHQ-9), Alcohol consumption risk (USAUDIT-C), mental well-being (SWEMWBS), cannabis/opioid/nonmedical stimulant use (self-reported questions)	NR	NR	The intervention group had greater reductions in anxiety (*p*<.001) and depressive symptoms (*p*=.007) than control.

Abbreviations. Acceptance and Action Questionnaire-II, AAQ-II; Adapted Gender Relations Scale, AGRS; Adapted Intimate Partner Violence Attitudes Scale, AVAS; Adapted WHO Multi-country Study Instrument, AWHO; ADHD Self-Assessment Scale, ADHS-SB; Attention-Deficit Hyperactivity Disorder, ADHD; Behavior Change Techniques, BCT; Center for Epidemiologic Studies Depression Scale Revised, CESD-R; Cognitive Fusion Questionnaire-7, CFQ-7; Conner's Adult ADHD Rating Scale, CAARS; Depression Anxiety Stress Scale, DASS; Depression Anxiety Stress Scales-21, DASS-21; Differential Emotions Scale, DES; Dysfunctional Beliefs and Attitudes about Sleep, DBAS-16; Eating Disorder Examination Questionnaire, EDE-Q; Edinburgh Postnatal Depression Scale, EPDS; Epworth Sleepiness Scale, ESS; Fatigue Assessment Scale, FAS; Fear of Food Questionnaire, FFQ; Flourishing Scale, FS; Gastrointestinal Cognitions Questionnaire, GI-COG; Gastrointestinal Symptom Rating Scale-IBS, GSRSIBS; General Anxiety Disorder-7, GAD-7; Headache Management Self-Efficacy, HMSE-G-SF; Insomnia Severity Index, ISI; Integrated Diagnosis of ADHD in Adulthood Revised Version, IDA-R; Interoceptive Accuracy Scale, IAS; Irritable Bowel Syndrome, IBS; Japanese Version of the Cardiff Fertility Knowledge Scale, CFKS-J; Mental Health Self-Efficacy Scale, MHSES; Modified Version of the Differential Emotions Scale, DES; Multiple-Choice Word Test, MWT-B; Not report, NR; Patient Health Questionnaire, PHQ; Patient Health Questionnaire Anxiety and Depression Scale, PHQ-ADS; Patient Health Questionnaire for Adolescents, PHQ-A; Patient-Reported Outcomes Measurement Information System, PROMIS; Penn State Worry Questionnaire, PSWQ; Perceived Stress Scale, PSS; Point Prevalence Abstinence, PPA; Positive and Negative Affect Schedule, PANAS; Positive and Negative Affect Schedule Short-Form, PANAS-SF; Quality of Life, QOL; Quick Inventory of Depressive Symptomatology-Self-Report, QIDS-SR; Revised UCLA Loneliness Scale, R-UCLA; Satisfaction With Life Scale, SWLS; Self-Rating Anxiety Scale, SAS; Short Compulsive Internet Use Scale, CIUS-5; Short Warwick-Edinburgh Mental Wellbeing Scale, SWEMWBS; Sleep Condition Indicator, SCI; Sleep-Related Behaviors Questionnaire, SRBQ; Sociocultural Attitudes Towards Appearance Questionnaire-4-Revised, SATAQ-4R; State-Trait Anxiety Inventory, STAI; University of Rhode Island Change Assessment, URICA; US Alcohol Use Disorders Identification Test–Consumption Scale, USAUDIT-C; Visceral Sensitivity Index, VSI; Weight Concerns Scale, WCS; World Health Organization Quality of Life Questionnaire, WHOQOL; 21-item Interoceptive Accuracy Scale, IAS.

Table S3. Summary of chatbot design features

Author, Year	Chatbot name	Main purpose	Chatbot Session	Chatbot content	Safety Measure	Reminder	Therapeutic approach	Deployment	Delivery Platform	dialogue system methods	AI techniques	Interaction mode	Evaluation approach	User engagement	User experience
Bricker, 2024[16]	QuitBot	Smoking cessation	Users were encouraged to complete 2- or 3-minute focused conversations for 42 days.	4 processes (bond, goal, tasks, perception) were integrated into dialogues over various SC phases, including strategies of expressing empathy, engaging in social dialogue, using metarelational communication (i.e., discuss the relationship), and expressing happiness while interacting with the user.	NR	Yes	Therapeutic alliance	Web-based	Facebook messenger	Retrieval-based	NLU, ML[a]	Textual	Automated evaluation: a scoring system of entropy (pertinence) and perplexity (grammaticalness) scores to rate the quality of answers; Manual evaluation: trained raters hand scored the answer accuracy, repetitiveness, and tone.	The completion rate of survey was 96% and the data retention did not differ between study arms (*p*=.54); Participants interacted with QuitBot was 1.3 times greater as compared to SFT (*p*=.02); Participants used the QuitBot 11 days longer than SFT (*p*=.001).	1) Positive feedback: a strong overall bond; 2) Negative feedback: inability to answer open-ended questions.
Chan, 2024[51]	Sleep Sensei	To deliver different types of coaching for people suffering insomnia	Participants were encouraged to engage in modules of talk system and tools system weekly.	Reflection, goal setting, action planning, positive feedback, problem-solving, summary and quiz.	NR	No	CBT	Standalone	Smartphone app	Rule-based	NR	Textual	User evaluation: a 7-point Likert-scale on Perceived ease of use, Perceived enjoyment, Perceived usefulness and Intention to use (MobileCoach platform)	26.9% (7/26) attrition in intervention, 33.3% (7/21) attrition in control group 1, 10% (3/30) attrition in control group 2; No significant difference between dCBTi-chatbot group and dCBTi-unguided group in completing video sessions; No significant difference in weeks of diaries completed among dCBTi-chatbot, dCBTi-unguided, and dSH group.	NR
Lee, 2023[14]	Coach Xiaonan	To help individuals who struggle	Participants were suggested to engage	Introduction of substance use disorder, MBRP sessions,	Crisis helpline established by	Yes	MBRP	Web-based	LINE messenger	Retrieval-based	NLP, ML, DL	Textual, visual	Expert evaluation: MARS	34% (17/50) attrition in intervention, 54.5% (24/44)	84% reported satisfaction, and 85% reported it as helpful; 2%

		with MA abuse	in a 2-month weekly schedule.	mindfulness skills practice, tips for early recovery, suggestions on life balance, mood tracking, positive feedback, psycho-educative materials.	government									attrition in control; 142.42±60.54 retention days on average in the experimental group and 118.12±73.41 days in the control group; The 6-month completion rates for the experimental and control groups were 66% and 51%; Participants with more severe MA use disorder (*p*=.023), low readiness to change (*p*<.001), and polysubstance use (p=.030) tended to have shorter retention periods.	experienced technical difficulties.
De Filippo, 2023[36]	None (Attention; Gamified; Narrative)	To improve gender attitudes in order to reduce IPV exposure among young women	Participants were instructed to complete 1-2 modules each day in the first 3 consecutive days and engage in module 6 "coping mechanisms" from day 4 to 3 months.	T0: basic information; T1: critical reflection, skills practice, healthy communication and coping mechanisms, self-efficacy building, safety planning, gamification; T2: storytelling	A direct chat link to a trained counsellor	No	Healthy coping	Web-based	WhatsApp	NR	NR	Textual, auditory	NR	62.5% (2456/3930) attrition in control, 90.7% (3563/3929) attrition in T0, 80.9% (4764/5891) attrition in T1, 82.3% (4850/5893) attrition in T2.	NR
Drouin, 2022[52]	Replika	To help form emotional connections and become weak ties in the outer layers of one's	Users were involved in a 20-min conversation.	Free topics as users wanted.	NR	Yes	CBT, emotional intelligence principles	web-based	Online	Generative	DL, NLP	Textual, auditory, visual	User evaluation: a 7-point Likert scale for assessing relationship and	No attrition in three study arms.	1) Perceived degree of similarity: Compared to the Replika group, both the participants in the F2F and the online chat groups viewed themselves

		acquaintance network and available for social support.											conversational dynamics		more similar to their conversational partner, rated their partner more responsive, and reported liking their conversation partner more;<br>2) Self-presentation concerns: Participants reported the highest levels of self-presentation concern in the F2F and online chat groups, whereas those in the Replika group reported the lowest levels of self-presentation concerns;<br>3) Likability: Participants in the Replika condition reported that their conversational partner liked them more than those in the online chat with human; Participants in the F2F chat group reported liking their conversation partner significantly more than those in the online chat.
Ehrlich, 2024[40]	Mind Tutor	To improve student well-being and attainment	Users were directed to complete interventions within topic identified by algorithms or chose another topic they needed over 6 weeks.	Goal-setting exercises, mindfulness exercises, suggestions on specific skills and actions, positive reframing, breathing exercises and focus timers.	NR	Yes	Mindfulness	Standalone	Smartphone app	Retrieval-based	NLU, NLP	Textual, auditory, visual	User evaluation: a 7-point Likert scale for assessing helpfulness	Trial 1: 64.1% (59/92) attrition in intervention and 41.5% (34/85) attrition in control; a consistently low engagement with the app on most days ranging between two to five daily interactions;<br>Trial 2: 64% (80/125)	The ratings for perceived helpfulness to become happier and to achieve better grades in both trial 1 and 2 were below the mid-point of the scale.

														attrition in intervention and 51.2% (64/125) attrition in control; Engagement in the first 14 days was reasonably high with an average of 9.7 interactions a day; Engagement after the first 14 days dropped significantly with an average of 1.8 interactions a day.	
Fitzpatrick, 2017[17]	Woebot	To deliver self-help program for college students self-identify as having symptoms of anxiety and depression	Participants were encouraged to use chatbot every day.	Psychoeducational content adapted from self-help for CBT, empathic responses, tailored and personalized message, goal setting, accountability, motivation and encouragement, reflection.	Long-standing depression, suicidality, or self-harm monitoring; helpline numbers; a crisis text line number; participants were encouraged to call 911 in emergencies.	Yes	CBT	Web-based	Instant messenger apps	Rule-based	NLP	Textual, visual	User evaluation: a 5-point Likert scale for satisfaction and learning	8.8% (3/34) attrition in intervention, 30.6% (11/36) attrition in control; an average of 12.14 (2.23) times of check-ins	1) The best experience: accountability (n=9); empathy, personality (n=7); facilitating learning (n=12);<br>2) The worst experience: process violations (e.g., repetitive conversations, not being able to understand some responses, getting confused when unexpected answers were provided, n=15); technical problems (e.g., technical glitches and looping conversational segments, n=8); problems with content (e.g., emotions, interactions, length, n=8)<br>3) Participants in the Woebot condition reported significantly higher levels of

															satisfaction both overall (*p*<.001) and with content (*p*=.02), and a significantly greater amount of emotional awareness than the information control group (*p*=.021).
Fitzsimmons-Craft, 2022[43]	Tessa	To reduce ED risk factors for women at high risk of ED	Participants were encouraged to take 1 month to complete two conversations a week.	Conversations based on topics including challenging the thin body ideal, media literacy, 4Cs (comparisons, conversations, commercials, and clothing), healthy eating, critical comments, exercise, binge eating, and maintenance, with warm and positive responses.	Crisis hotline triggered based on keywords such as "hurting myself"	Yes	CBT	Web-based	SMS or Facebook Messenger	Rule-based	NR	Textual, visual	Interaction transcripts were reviewed by 7 mental health professionals to identify bugs, chatbot responses that were erroneous or problematic, and unsmooth conversations; User evaluation on helpfulness	41.2% (145/352) attrition in intervention, 33.3% (116/348) attrition in control; An average of 4 total interactions, 11 min per interaction, across an average of 16 days.	More than 65% of participants rated conversations as helpful.
Fulmer, 2018[15]	Tess	To reduce self-identified symptoms of depression and anxiety in college students	Group 1 received daily messages from Tess over a 2-week period, Group 2 received biweekly messages over a 4-week period.	Customized messages, journaling and relaxation strategies, empathic responses, accountability.	Suicidal/homicidal ideation monitoring; national suicide prevention hotline; crisis text line; 911; users were encouraged to end the chat and reach out for professional help.	Yes	CBT, EFT, ACT, MI, mindfulness-based therapy, self-compassion therapy, IPT	Web-based	instant messenger apps (Facebook, Slack, SMS)	Retrieval-based	NLP, ML, emotion algorithms	Textual	User evaluation: a user satisfaction survey	No attrition in chatbot, 4% (1/25) attrition in the control; Group 1 exchanged 283 (147.6) daily messages for 2 weeks; Group 2 exchanged 286 (104.6) biweekly messages.	1) The best experience: accountability (15/50); empathy (6/50); facilitating learning (11/50);<br>2) The worst experience: unnatural conversation (12/50); not being able to understand some responses, getting confused when unexpected answers were provided (11/50); not interactive content (7/50).<br>3) 2/48 reported meaningfulness of interaction with Tess; 86% participants were overall satisfied with Tess and only

															60% with the eBook; More participants (80%) reported they learned something new from Tess than 43% from the eBook;
Greer, 2019[39]	Vivibot	To deliver positive psychology skills for improving psychological well-being in young adults treated for cancer	Users were notified to interact with chatbot daily.	Repeated 3 times of 7 conversational teaching lessons and 7 practice lessons covering 8 positive psychology skills (noticing and acknowledging positive events, savoring positive events, gratitude, positive reappraisal, acts of kindness, mindfulness, personal strengths, and attainable goals), and six daily emotion ratings.	NR	Yes	Stress and coping theory; the Broaden-and-Build theory of positive emotion; positive psychology	Web-based	Facebook messenger	Rule-based	NR	Textual, visual	User evaluation: a brief scale for assessing perceived helpfulness and intention to recommend	36% (9/25) attrition in intervention, 15% (3/20) attrition in control at 4 weeks;<br>Participants in the intervention group spent an average of 73.8 (52) min across an average of 12.1 (7.1) engaged sessions with Vivibot.	1) positive experience: a great alternative path to talk about treatment and life; sharing experience with others and being able to vent; positive psychology content;<br>2) negative experience: less helpfulness<br>3) participants in the intervention group rated Vivibot as helpful (2.03/3, SD 0.72) and were willing to recommend it to friends (6.9/10, SD 2.6).
Haug, 2022[50]	Ready4life	To prevent addiction among apprentices by promoting life skills and reducing risk behaviors	Participants were asked to take average 2-5 min to process 16 weekly dialogues within two optional topics for 8 weeks.	6 dialogue modules including stress, social skills, social media & gaming, tobacco/e-cigarette smoking, cannabis, and alcohol; tailored messages, feedback on current substance use and life skills, quiz, challenge, and competition.	NR	Yes	SCT, MI, Social Norms Approach	Standalone	Smartphone app	Rule-based	NR	Textual, visual	User evaluation on comprehensibility, helpfulness, and personalization	36% (248/688) attrition in intervention, 23.2% (115/663) attrition in control;<br>The average completed dialogues was 2.1 (3.5).	Comprehensible (93.4%), helpful (83.1%), tailored (81.2%)
He, 2022[45]	XiaoE	To provide screening, prevention, and self-assistance for depressive symptoms among	Participants were asked to sequentially complete one module per day in 1 week, and	7 modules corresponding to psychology, cognitive distortions, self-esteem, mindfulness meditation,	NR	Yes	CBT, mindfulness	Web-based	WeChat official accounts platform	Retrieval-based	NLP, DL	Textual, auditory, visual	User evaluation: WAQ; UMUX-LITE; AS	8.16% (4/49) attrition in XiaoE, and 20.4% (10/49) attrition in control at posttest;	1) The best experience: relationship (n=25), emotion (n=12), personalization (n=31), practicability (n=80);

		young adults during the COVID-19 pandemic	record positive events and mood every day.	mental energy, natural connection, self-help, and loneliness, with multiturn dialogue and personalized customization.									Participants interacted with XaioE for 25.54 (26.45) sessions on average per day, with each session lasting an average of 22.46 (79.88) seconds; The frequency of use reached peaks during 8-10am, 12-2pm, and 4-6pm.	2) The worst experience: inflexible/tedious/repetitive/mechanical content (n=120), technology (n=28). 3) Participants in the XiaoE group scored higher on the total WAQ (*p*=.04), bond (*p*=.02), and engagement (*p*=.02) compared with the control group; 4) Better acceptability in the XiaoE group for total AS (*p*=.02), content satisfaction (*p*=.007), emotional awareness (*p*=.03), learning new knowledge (*p*=.02), and relevance to daily life (*p*=.009).
Hunt, 2021[47]	Zemedy	To mitigate the impact of IBS on daily life and improve health-related quality of life	Participants were encouraged to read through the first 5 modules (education, relaxation training, exercise) in the first week, and to practice relaxation exercises daily. The remaining modules should be completed about one per week, with practice and homework	10 modules for psychoeducation and CBT techniques (relaxation training, exercise, cognitive restructuring and decatastrophizing, exposure exercises to reduce avoidance, and behavioral experiments), as well as an additional module for immediate problem solving.	Depressive symptoms and suicidal ideation monitoring, emergency contact, risk assessment, referrals to local resources	Yes	CBT, stress management, exposure therapy	Standalone	smartphone app	Retrieval-based	NLU	Textual, auditory	NR	42% (26/62) attrition in intervention, 25% (15/59) attrition in control at posttest; An average of 3.2(2) modules were completed.	NR

			exercises performed daily to learn and apply the skills.											
Jang, 2021[48]	Todaki	To help people with attention deficit get proper psychoeducation and learn self-help skills to manage their condition	Users were required to check-in and finish daily assessments and programs every day.	Self-assessment, self-help program (behavior change, emotional control, medication, and mindfulness), psychoeducation session, empathic responses, reflection, quiz, daily mood tracking, check-in records.	Referrals to professionals for diagnosis and treatment in severe cases	Yes	CBT, mindfulness	Standalone	Smartphone app	Rule-based	NLP	Textual, visual	User evaluation: a user satisfaction survey	17.4% (4/23) attrition in intervention, 21.7% (5/23) attrition in control; Users checked into the chatbot 20.32 (12.89) times over 4 weeks, using 1h 15min (1h 20min) for 13.53 (8.24) days on average.	1) The best experience: empathic and friendly mascot (n = 7), daily check-up (n = 6), easy explanation (n = 6); 2) The worst experience: unnatural conversation (n = 7), non-intuitive interfaces (n = 6); 3) No significant group difference in acceptability or adverse effects.
Karkosz, 2024[49]	Fido	To provide mental health support to adolescents and young adults struggling with anxiety and depression	Users accessed therapeutic techniques after receiving basic training in cognitive biases.	ABC model of CBT, psychoeducation, gratitude practice exercise, cognitive biases recognition and Socratic questioning.	Suicidal ideation monitoring, emergency hotlines	Yes	CBT	Web-based	Facebook messenger	Retrieval-based	ML, NLU	Textual	User evaluation: WAI-SR, Acceptability E-scale, HAIS, LPS	17.5% (7/40) attrition in intervention, 14.6% (6/41) attrition in control; Similar frequency of use (*p*=.15) and scores on the knowledge test *p*=.99) in two groups; The control group spent more time reading the book than the chatbot group spent on the interaction with Fido (117.57[72.4] vs 79.44[42.96], *p*=.008).	1) Negative correlation between age and the WAI-SR score (*p*=.04); 2) Positive correlation between the WAI-SR score and the subjective sense of being assessed by LPS (*p*<.001), overall acceptability (*p*<.001), and scores for competence (*p*<.001) and behavioral traits (*p*=.01) assessed by HAIS; 3) positive feedback: encouragement to start traditional therapy; 4) negative feedback: failures in recognizing the user's intent.
Klos, 2021[53]	Tess	To examine and reduce depression	The chatbot initiate the conversatio	Psychoeducational content, emotional	Suicidal/homicidal	Yes	CBT, EFT, SFBT, MI	Web-based	Facebook messenger	Retrieval-based	NLP, emotion algorithm	Textual	NR	60.6% (60/99) attrition in intervention,	1) Positive (25/39): the capability to understand people

		and anxiety for university students	n once a day during the initial weeks and every other day in the following weeks.	support, empathetic and customized responses, relaxation strategy.	ideation monitoring; national suicide prevention hotline; crisis text line; 911; users were encouraged to end the chat and reach out for professional help.									48/82 (58.5%) attrition in control; an average of 472 (249.52) exchanged messages.	2) Ambivalent or negative (7/39): inconsistent answers to the questions<br>3) Positive correlation between number of messages exchanged with Tess and positive feedback (*p*=0.02)
Lavelle, 2021[54]	None (defusion; restructuring)	To deliver cognitive defusion or restructuring strategies for treating negative self-referential thoughts	Participants were instructed to engage in conversations and exercises for 10 min each day.	Brief daily conversation, mood tracking, exercises.	NR	Yes	CBT, ACT	Web-based	Facebook messenger	Rule-based	/	Textual, auditory, visual	NR	68.06% (49/72) attrition in defusion group, 73.75% (59/80) attrition in restructuring group, 74.65% (53/71) attrition in control.	NR
Liu, 2022[21]	XiaoNan	To alleviate depression for university students	Participants were asked to use the chatbot for 16 weeks.	CBT treatment, sympathetic feedback, emotion assessment, personal usage and mental status reports, daily check-in.	Emergency assessment and contact	No	CBT	Web-based	WeChat official accounts platform	Retrieval-based	NLU, ML, NLP, emotion algorithm	Textual, auditory	User evaluation (WAI-SR, CSQ-8)	19.51% (8/41) attrition in intervention group, 28.57% (12/42) attrition in control;<br>The adherence rates measured by a 4-point Likert scale for the chatbot and control group were 1.96 (0.70) with a downward trend, and 1.97(0.62) with an upward trend, respectively (*p*=.50).	1)The best experience: easy to access (11/33), empathy/friendly (8/33), interesting (7/33), exploring depression (9/33);<br>2)The worst experience: impersonal (8/33), unnatural (7/33), rigid patterns (7/33), repetitive contents (10/33), too general (8/33)<br>3) The chatbot group revealed better therapeutic alliance than the control (*p*<.01); No significant group difference in satisfaction (*p*=.38).

Ly, 2017[55]	Shim	To help people reflect upon, learn and practice strategies for promoting mental well-being	Participants were encouraged to complete at least 14 reflections over the course of 14 days.	Empathic responses, tailored content, daily check-ins, weekly summaries, reflections, CBT and positive psychology insights and activities.	NR	No	positive psychology, CBT	Standalone	smartphone app	Rule-based	/	Textual, auditory	NR	7.14% (1/14) attrition in intervention group, no attrition in control;<br>The average active days was 8.21 (3.0), opening the app 1.27 times per day.	1) Positive feedback: positive psychology; encouragement to express gratitude; reflection assistance; routine; driving the conversation; like a living character; weekly summaries;<br>2)Negative feedback: lack of clarity of aim and goals, notifications, and depth; repetitiveness; not following up on some certain questions.
Maeda, 2020[56]	None	To improve knowledge, intentions to preconception behavioral change and anxiety for women who are hoping to have children	Participants were instructed to spend about 1 hour learning and chatting.	Counselling and educational conversations focused on factors with a significant effect on fertility and preconception health.	NR	No	Transtheoretical model of change	Web-based	Online	Retrieval-based	NLP	Textual	NR	No attrition in three arms;<br>a total of 574 chatbot sessions were recorded with an average length of 8 min.	1) Positive feedback: increased knowledge and awareness (41.5%), promoting understanding more than just reading (10.2%)<br>2) Negative feedback: superficial content and lack of details (10.9%), technical problems (10.2%), low comprehension of the chatbot (28%), burdensome and unnecessary conversations (5.5%), lack of humanity or empathy (6.2%), slow operation (5.5%), quick display of information (5.1%).
Matheson, 2023[44]	Topity	To address risk and protective factors of body image for adults	Users were encouraged to take 5 to 10 min for each technique	8 micro-intervention techniques clustered into 3 themes including 'family, friends and body image',	Mental health support services	Yes	CBT, positive body image and embodiment theory	Web-based	Facebook messenger	Rule-based	/	Textual, auditory, visual	User evaluation on acceptability	58.62% (503/858) attrition in intervention group, 48.31% (414/857) attrition in	Users rated an overall score of 6.07 out of 7 on acceptability.

			during 72 hours.	'social media and body image', and 'body appreciation and functionality'; gamified interaction.										control at posttest; participants completed 5 techniques on average.	
Nicol, 2022[57]	Woebot	To help the adolescent newly diagnosed with depression and anxiety develop emotion regulation skills in the context of their everyday life for the problem at hand	Users were prompted to check in the chatbot daily and receive education based on their reported mood or desire. √	Mood tracking, goal-oriented, tailored conversations, CBT psychoeducation and tools, daily check-in.	Safety monitoring and alert, crisis hotline number, case review, suicide risk assessment, consultation and suggestions offered by psychiatrists	Yes	CBT, DBT, IPT-A	Standalone	Smartphone app	Rule-based	NLP, ML	Textual, visual	User evaluation (AIM, FIM, SUS)	No attrition in chatbot group, 12.5% (1/8) attrition in control at posttest; Participants' app use averaged a mean of 6 (6.9) days, with 55 (7.14) mood check-ins, and 313.17 (447.30) sent messages.	Acceptable, with 80% reporting they liked using the app; feasible, with 70% thinking it seemed possible to use the app to treat depression; usable, with an average score of 21.4 (1.7) for usability.
Romanovskyi, 2021[46]	Elomia	To reduce the tendency to anxiety, depression, and experiencing negative emotional states	Unlimited access to chatbot at any time of the day to the extent that users needed for 4 weeks.	Cognitive-behavioral psychotherapy, including grounding technique and exercises for calming, falling asleep, breathing, improving self-esteem, and reducing anxiety.	Offering support including first-aid techniques	No	CBT, narrative psychotherapy	Standalone	Smartphone app	Generative	GPT, BERT, emotion algorithm	Textual	NR	NR	Helpful, with over 70% of users returning to using it when suffering increased anxiety, panic attack, self-doubt, loneliness.
Sabour, 2023[58]	Emohaa (CBT-bot; ES-bot)	To provide cognitive and emotional support to reduce symptoms of mental distress including depression, anxiety, negative affect, and insomnia	Participants were instructed to use CBT-bot daily or converse with full bot at least once daily, with each session lasting for 5-10 turns.	1) CBT-bot: automatic thoughts training and over 20 guided expressive writing;<br>2) ES-bot: free topics discussions supported by strategies including self-disclosure, affirmation, and suggestions.	Suicidal risk assessment, emergency contact	No	CBT, Helping Skills Theory, expressive writing	Web-based	WeChat messenger	1) CBT-bot: Rule-based<br>2) ES-bot: Generative	ES-bot: GPT	Textual	User evaluation: a 10-point Likert scale for ease of use, provided content, and interface quality	57.78% (52/90) attrition in CBT-bot, 61.11% (55/90) attrition in ES-bot, and 46.28% (56/121) attrition in chatbot group 1, 2, and control at posttest, respectively.	1) Positive feedback: For CBT-bot, participants were satisfied with functionality (81.16%), exercises (68.12%), interface (62.32%); For ES-bot, participants considered it as an appropriate chatting partner (77.42%), channel for emotional venting

															(64.5%), felt heard (58.1%), expected it to offer emotional companionship and support that can accurately interpret their emotions and provide counseling (21/ 31, 67.74%). 2) Negative feedback: technical issues, unclear instructions, and limited content and choices for CBT-bot; Rigid conversations, unrelated and out-of-context responses for the ES-bot.
Schillings, 2024[37]	ELME	To reduce stress and improve various health-related parameters for people who experienced stress	Sessions were held twice a day, for approximately 10-20 min each, over 3 weeks and with flexible timing.	Psychoeducation including modules of stress, interoception (e.g., Heartbeat perception exercises), mindfulness, and association of them (e.g., breathing exercise); EMA	NR	Yes	MBSR, Stress Management, ACT	Web-based	a link through SMS	Rule-based	/	Textual, auditory	User evaluation (German version of the 18-item Mental Health App Usability Questionnaire)	35.59% (21/59) attrition in chatbot group, 28.81% (17/59) attrition in control at posttest.	The mean score for usability was 2.55 (0.68); 22 participants reported technical problems.
Selaskowski, 2023[59]	None	To reduce ADHD core symptoms in adults	Participants were asked to engage in psychoeducation as much as possible, with approximately 16 hours required to process all the content.	8 psychoeducational modules containing basic information about ADHD, personal resources, mindfulness and attention control, self-organization, stress management, mood regulation and impulsive behavior control, relationships, and quizzes.	Adverse event monitoring	No	Mindfulness, self-organization, stress management	Standalone	Smartphone app	Rule-based	/	Textual	NR	15% (3/20) attrition in both chatbot and control group.	NR
Stewart, 2023[38]	Expressive Interviewing System	to encourage people to explore their thoughts and	Participants were directed to spend at	Each conversation consisted of 4 main writing prompt questions	NR	No	Expressive writing; MI	Web-based	online	Rule-based	/	Textual, visual	NR	12% (12/100) attrition in chatbot group, 37% (37/100)	NR

		feelings surrounding features of COVID-19 to promote positive changes in mental health and health-related behavior	least 15 minutes on their writing task.	about specific issues related to the pandemic, reflection, empathic responses, affirmation.										attrition in control.	
Suharwardy, 2023[60]	Woebot	To assist in mood management for general postpartum population	Participants were encouraged to use chatbot daily with the opportunity to optout of daily check-in.	Psychotherapeutic techniques, mood tracking with patterns identification; daily check-in.	911 for emergency service, suicide crisis hotlines, and physicians	Yes	CBT, IPT, DBT	Standalone	smartphone app	Rule-based	NLP, ML	Textual	User evaluation: WAI-SR, CSQ-8	29.17% (28/96) attrition in chatbot group, 12.5% (12/96) attrition in control;<br>Most patients (74%) reported using the chatbot at least once.	An average of 24.0 (5.7) for CSQ-8 satisfaction score, and 2.97 (1.35) for WAI-SR.
Ulrich (BalanceUP), 2024[25]	BalanceUP	To deliver treatment and improve mental well-being for people experiencing frequent headaches	Users were allowed to work through units within 24-60 days according to their preferences.	7 modules including Psychoeducation, relaxation, balanced lifestyle, coping with fear of headache attack, coping with headaches, headache trigger, and stress management; an outro for appreciation and reflection.	Adverse event monitoring using the Patient Global Impression of Change Scale	Yes	BCT, CBT, self-determination theory	Standalone	Smartphone app	Rule-based	/	Textual, auditory, visual	User evaluation: the German Group Therapy Session Evaluation by Patients, the Session Alliance Inventory - Patient Version, uMARS	29.09% (32/110) attrition in chatbot group, 16.16% (16/88) attrition in control;<br>86.3% of participants completed all 7 modules, taking an average of 6.9 (1.5) days to work through a module. Participants replied in an average of 77.8% (3.73%) conversational turns.	1)positive feedback: the extensive and comprehensive information<br>2)negative feedback: poor flexibility within interactions<br>3) An average score of 3.72(0.65) for engagement, 4.47 (0.47) for Information, 3.56 (0.77) for perceived app quality, and 4.00 (0.62) for perceived impact of the app measured by uMARS; the alliance significantly increased with time (*p*<.001)
Ulrich (MISHA), 2024[41]	MISHA	To empower students to reflect on their daily stressors and effectively manage their	Participants were allowed to schedule sessions every 2 to 4 days,	Goal setting, guided self-reflection, instructions and information about relaxation techniques and	NR	Yes	HAPA, CBT, Mindfulness	Standalone	Smartphone app	Rule-based	/	Textual, auditory	User evaluation (uMARS, WAI-SR)	40.00% (28/70) attrition in chatbot group, 20.00% (14/70) attrition in control;	1)Positive feedback: visualization, emojis, avatars, content, customization,

		stress with new coping techniques	completing a consecutive 12-session coaching program in 24 to 54 days.	mindfulness, psychoeducation and basic information regarding stress and its symptoms, student specific topics such as anxiety, learning techniques or time management, customization.										45% of the completers worked through all 13 sessions, played 86.52 (120.54) min of relaxation audios on average. MISHA sent 400 (205.61) messages and participants answered a mean of 297.54 (169.80) messages, with an average engagement ratio of 74.3%.	reminder, chat format 2)Negative feedback: long conversations, inappropriate answer options, language style, excessive stress and pressure, technical issues 3)suggestions for improvement: customization due to degree of stress, add-ons for recording own messages, changeable voice 4) An average score of 4.23 (0.89) for WAI-SR; for uMARS, the subscale information was scored highest, and individual customization was rated lowest, with only a few participants (2%) showed a high willingness to pay for the app or anticipated high future use.
Verescha gin, 2024[42]	Minder	To provide a self-directed tool for students to improve their mental health and manage substance use	Participants were instructed to use the app as they wanted.	Chatbot activities and services covering topics of University Life, Wellbeing, Relationships, Sadness, Stress, Anxiety, and Substance Use.	Local crisis resource s, emergen cy monitori ng and contact	Yes	CBT, DBT, mindfulness, metacognitive training, motivational interviewing	Standalo ne	Smartphone app	Rule-based	/	Textual, visual	NR	20.46% (152/743) attrition in chatbot group, 17.02% (127/746) attrition in control; 77.1% of users accessed at least 1 chatbot activity.	NR

[a]GPT was used for chatbot refinement after the trial.

Abbreviations. Acceptance and Commitment Therapy, ACT; Acceptability of Intervention Measure, AIM; Acceptability Scale, AS; Bidirectional Encoder Representations from Transformers, BERT; Client Satisfaction Questionnaire-8, CSQ-8; Cognitive Behavioral Therapy, CBT; Deep Learning, DL; Dialectical Behavior Therapy, DBT; Ecological Momentary Assessments, EMA; Emotion-Focused Therapy, EFT; Feasibility of Intervention Measure, FIM; Generative Pre-trained Transformer, GPT; Health Action Process Approach Model, HAPA; Human-Agent Interaction Scale, HAIS; Interpersonal Psychotherapy, IPT; Interpersonal Psychotherapy for Adolescent, IPT-A; Language Pragmaticality Scale, LPS; Machine Learning, ML; Mindfulness-Based Relapse Prevention, MBRP; Mindfulness-Based Stress Reduction, MBSR; Mobile Application Rating Scale, MARS; Motivational Interviewing, MI; Natural Language Processing,

NLP; Natural Language Understanding, NLU; Social Cognitive Theory, SCT; Solution-Focused Brief Therapy, SFBT; SmokefreeTXT, SFT; System Usability Scale, SUS; Usability Metric for User Experience-LITE, UMUX-LITE; User Version of the Mobile App Rating Scale, uMARS; Working Alliance Inventory-Short Revised, WAI-SR; Working Alliance Questionnaire, WAQ.

Table S4. Results of subgroup analysis

	Hedges' g (95% CI)	*P* value	I^2 (%)	n[a]
**Overall mental distress**				
Control group types		*0.019*		
Active control	-0.082 (-0.316 to 0.152)	0.491	53.9%	9
Information control	-0.687 (-1.036 to -0.338)	<0.001	89.2%	14
Passive control	-0.268 (-0.368 to -0.168)	<0.001	71.4%	26
Intervention duration		0.375		
0-4 weeks	-0.349 (-0.536 to -0.161)	<0.001	86.6%	24
5-8 weeks	-0.401 (-0.547 to -0.254)	<0.001	74.4%	18
⩾9 weeks	-0.337 (-0.443 to -0.232)	0.023	62.2%	6
Target sample		*0.003*		
Clinical	-0.261 (-0.497 to -0.025)	0.030	67.7%	16
Subclinical	-0.597 (-0.826 to -0.369)	<0.001	89.1%	18
Nonclinical	-0.169 (-0.260 to -0.078)	<0.001	54.6%	14
Reminders		0.208		
Yes	-0.273 (-0.362 to -0.183)	<0.001	64.1%	34
No	-0.493 (-0.823 to -0.162)	0.003	91.5%	14
Interaction mode		0.864		
Text-only	-0.362 (-0.624 to -0.101)	0.007	89.1%	18
Multimedia	-0.338 (-0.438 to -0.237)	<0.001	69.5%	30
Deployment		*0.027*		
Web-based	-0.225 (-0.329 to -0.122)	<0.001	54.4%	18
Standalone	-0.449 (-0.618 to -0.280)	<0.001	86.5%	30
Dialogue system methods		*0.007*		
Generative	-1.121 (-2.059 to -0.183)	0.019	96.8%	11
Retrieval-based	-0.479 (-0.666 to -0.291)	<0.001	58.2%	35
Rule-based	-0.211 (-0.293 to -0.129)	<0.001	57.8%	5
**Depression**				
Control group types		0.061		
Active control	-0.042 (-0.510 to 0.426)	0.861	65.3%	3
Information control	-0.735 (-1.118 to -0.353)	<0.001	73.1%	6
Passive control	-0.324 (-0.535 to -0.114)	0.003	77.3%	8
Intervention duration		0.268		
0-4 weeks	-0.401 (-0.698 to -0.103)	0.027	80.7%	10
5-8 weeks	-0.413 (-0.724 to -0.102)	0.009	83.2%	6
⩾9 weeks	-0.818 (-1.267 to -0.369)	<0.001	N/A	1
Target sample		0.103		
Clinical	-0.308 (-0.782 to 0.166)	0.203	77.1%	6
Subclinical	-0.606 (-0.936 to -0.276)	<0.001	84.3%	8
Nonclinical	-0.171 (-0.396 to 0.053)	0.135	62.1%	3
Reminders		0.580		
Yes	-0.357 (-0.539 to -0.176)	<0.001	71.6%	12
No	-0.536 (-1.142 to 0.070)	0.083	89.4%	5
Interaction mode		0.725		
Text-only	-0.360 (-0.827 to 0.107)	0.131	86.9%	6
Multimedia	-0.452 (-0.666 to -0.239)	<0.001	77.9%	11
Deployment		0.325		
Web-based	-0.314 (-0.519 to -0.108)	0.003	57.2%	7
Standalone	-0.512 (-0.850 to -0.174)	0.003	87.3%	10
Dialogue system methods		*0.030*		
Generative	-0.958 (-2.342 to 0.426)	0.175	95.4%	2
Retrieval-based	-0.673 (-0.995 to -0.351)	<0.001	49.3%	4
Rule-based	-0.222 (-0.375 to -0.070)	0.004	59.4%	12

**Anxiety**				
Control group types		0.231		
Active control	-0.096 (-0.586 to 0.395)	0.702	68.3%	3
Information control	-0.732 (-1.347 to -0.116)	0.020	93.6%	7
Passive control	-0.207 (-0.348 to -0.066)	0.004	50.0%	8
Intervention duration		0.823		
0-4 weeks	-0.447 (-0.818 to -0.076)	0.018	92.0%	10
5-8 weeks	-0.321 (-0.521 to -0.122)	0.002	59.8%	7
⩾9 weeks	-0.297 (-0.730 to 0.136)	0.179	N/A	1
Target sample		0.290		
Clinical	-0.210 (-0.485 to 0.064)	0.134	35.0%	6
Subclinical	-0.704 (-1.293 to -0.115)	0.019	94.4%	7
Nonclinical	-0.218 (-0.403 to -0.034)	0.020	70.8%	5
Reminders		0.121		
Yes	-0.211 (-0.338 to -0.084)	0.001	39.8%	12
No	-0.720 (-1.352 to -0.089)	0.025	95.0%	6
Interaction mode		0.224		
Text-only	-0.554 (-1.038 to -0.069)	0.025	93.4%	8
Multimedia	-0.241 (-0.382 to -0.099)	0.001	45.4%	10
Deployment		*0.054*		
Web-based	-0.174 (-0.329 to -0.019)	0.028	52.9%	8
Standalone	-0.624 (-1.054 to -0.194)	0.004	92.1%	10
Dialogue system methods		0.101		
Generative	-1.990 (-5.773 to 1.793)	0.303	98.8%	2
Retrieval-based	-0.362 (-0.481 to -0.243)	<0.001	0.0%	5
Rule-based	-0.179 (-0.319 to -0.039)	0.012	52.2%	12
**Positive affect**				
Control group types		0.292		
Active control	-0.181 (-0.417 to 0.055)	0.132	N/A	1
Information control	0.167 (-0.262 to 0.596)	0.446	71.7%	4
Passive control	0.010 (-0.207 to 0.228)	0.925	56.5%	6
Intervention duration		0.584		
0-4 weeks	0.076 (-0.133 to 0.285)	0.476	66.3%	8
5-8 weeks	-0.051 (-0.647 to 0.546)	0.868	75.8%	2
⩾9 weeks	-0.170 (-0.601 to 0.261)	0.439	N/A	1
Target sample		0.174		
Clinical	0.214 (-0.376 to 0.804)	0.476	N/A	1
Subclinical	0.171 (-0.096 to 0.438)	0.209	62.4%	5
Nonclinical	-0.126 (-0.334 to 0.082)	0.235	40.9%	5
Reminders		0.485		
Yes	-0.016 (-0.210 to 0.177)	0.868	55.0%	8
No	0.181 (-0.337 to 0.698)	0.494	82.7%	3
Interaction mode		0.369		
Text-only	0.220 (-0.290 to 0.730)	0.399	80.7%	3
Multimedia	-0.031 (-0.227 to 0.165)	0.758	57.2%	8
Deployment		0.484		
Web-based	-0.013 (-0.169 to 0.143)	0.873	41.8%	8
Standalone	0.228 (-0.427 to 0.883)	0.496	85.9%	3
Dialogue system methods		0.424		
Generative	0.144 (-0.347 to 0.636)	0.565	86.0%	3
Retrieval-based	-0.091 (-0.349 to 0.167)	0.490	30.1%	4
Rule-based	0.105 (-0.059 to 0.269)	0.208	18.6%	5
**Negative affect**				
Control group types		0.507		

Active control	-0.081 (-0.316 to 0.155)	0.501	N/A	1
Information control	-0.664 (-1.615 to 0.287)	0.171	93.6%	4
Passive control	-0.115 (-0.224 to -0.005)	0.040	0.0%	6
Intervention duration		0.072		
0-4 weeks	-0.410 (-0.745 to -0.075)	0.017	87.3%	8
5-8 weeks	0.068 (-0.221 to 0.357)	0.645	0.0%	2
⩾9 weeks	0.084 (-0.346 to 0.515)	0.701	N/A	1
Target sample		0.426		
Clinical	-0.126 (-0.714 to 0.463)	0.676	N/A	1
Subclinical	-0.535 (-1.158 to 0.088)	0.092	92.8%	5
Nonclinical	-0.109 (-0.254 to 0.037)	0.145	0.0%	5
Reminders		0.229		
Yes	-0.096 (-0.199 to 0.007)	0.067	0.0%	8
No	-0.793 (-1.924 to 0.339)	0.170	95.8%	3
Interaction mode		0.126		
Text-only	-0.935 (-2.033 to 0.163)	0.095	95.1%	3
Multimedia	-0.075 (-0.177 to 0.027)	0.152	0.0%	8
Deployment		0.413		
Web-based	-0.132 (-0.231 to -0.034)	0.008	0.0%	8
Standalone	-0.695 (-2.038 to 0.648)	0.310	96.2%	3
Dialogue system methods		0.205		
Generative	-0.833 (-1.801 to 0.134)	0.091	96.0%	3
Retrieval-based	-0.010 (-0.224 to 0.205)	0.930	0.0%	4
Rule-based	-0.136 (-0.255 to -0.018)	0.024	0.0%	5
**Stress**				
Control group types		0.575		
Active control	N/A	N/A	N/A	0
Information control	-0.582 (-1.173 to 0.010)	0.054	N/A	1
Passive control	-0.402 (-0.498 to -0.306)	0.000	0.0%	5
Intervention duration		0.120		
0-4 weeks	-0.679 (-1.150 to -0.208)	0.005	0.0%	2
5-8 weeks	-0.529 (-0.751 to -0.307)	<0.001	0.0%	3
⩾9 weeks	-0.363 (-0.471 to -0.256)	<0.001	N/A	1
Target sample		0.497		
Clinical	-0.620 (-0.979 to -0.260)	0.001	0.0%	2
Subclinical	-0.429 (-0.754 to -0.104)	0.010	N/A	1
Nonclinical	-0.387 (-0.490 to -0.284)	<0.001	20.8%	3
Reminders		0.252		
Yes	-0.400 (-0.495 to -0.304)	<0.001	0.0%	5
No	-0.849 (-1.627 to -0.070)	0.033	N/A	1
Dialogue system methods		0.293		
Generative	N/A	N/A	N/A	0
Retrieval-based	-0.642 (-1.094 to -0.190)	0.005	N/A	1
Rule-based	-0.396 (-0.493 to -0.299)	<0.001	0.0%	5
**Psychosomatic symptoms**				
Control group types		0.935		
Active control	-0.459 (-0.937 to 0.020)	0.060	N/A	1
Information control	N/A	N/A	N/A	0
Passive control	-0.485 (-0.899 to -0.071)	0.022	82.0%	4
Intervention duration		*0.002*		
0-4 weeks	-0.031 (-0.283 to 0.221)	0.811	N/A	1
5-8 weeks	-0.609 (-0.865 to -0.352)	<0.001	35.3%	4
⩾9 weeks	N/A	N/A	N/A	0
Target sample		*0.008*		

Clinical	-1.002 (-1.471 to -0.534)	0.000	N/A	1
Subclinical	-0.587 (-0.858 to -0.316)	<0.001	0.0%	2
Nonclinical	-0.155 (-0.465 to 0.156)	0.329	44.8%	2
Reminders		0.088		
Yes	-0.652 (-0.983 to -0.321)	<0.001	52.2%	3
No	-0.195 (-0.602 to 0.213)	0.350	58.4%	2
Interaction mode		0.088		
Text-only	-0.195 (-0.602 to 0.213)	0.350	58.4%	2
Multimedia	-0.652 (-0.983 to -0.321)	<0.001	52.2%	3
Deployment		*0.002*		
Web-based	-0.031 (-0.283 to 0.221)	0.811	N/A	1
Standalone	-0.609 (-0.865 to -0.352)	<0.001	35.3%	4
Dialogue system methods		*0.001*		
Generative	0.057 (-0.245 to 0.360)	0.710	N/A	1
Retrieval-based	-1.002 (-1.471 to -0.534)	<0.001	N/A	1
Rule-based	-0.384 (-0.636 to -0.132)	0.003	46.4%	4
**Self-ambivalence and appearance distress**				
Target sample		0.654		
Clinical	N/A	N/A	N/A	0
Subclinical	-0.256 (-0.340 to -0.172)	<0.001	56.7%	3
Nonclinical	-0.067 (-0.888 to 0.753)	0.872	N/A	1
**Life satisfaction and well-being**				
Control group types		0.227		
Active control	0.186 (-0.288 to 0.659)	0.442	N/A	1
Information control	0.117 (0.025 to 0.208)	0.230	N/A	1
Passive control	0.130 (0.035 to 0.224)	0.007	47.1%	8
Intervention duration		0.166		
0-4 weeks	-0.125 (-0.478 to 0.229)	0.490	0.0%	3
5-8 weeks	0.134 (0.039 to 0.228)	0.005	54.5%	7
⩾9 weeks	N/A	N/A	N/A	0
Target sample		0.081		
Clinical	N/A	N/A	N/A	0
Subclinical	0.310 (0.075 to 0.545)	0.010	81.1%	3
Nonclinical	0.082 (-0.016 to 0.181)	0.102	0.0%	7
Reminders		0.906		
Yes	0.115 (0.021 to 0.209)	0.017	62.6%	7
No	0.137 (-0.214 to 0.488)	0.445	0.0%	3
Interaction mode		0.310		
Text-only	-0.051 (-0.387 to 0.285)	0.766	48.5%	2
Multimedia	0.130 (0.035 to 0.224)	0.007	47.1%	8
Deployment		0.088		
Web-based	-0.293 (-0.771 to 0.186)	0.230	N/A	1
Standalone	0.132 (0.039 to 0.225)	0.005	39.8%	9
Dialogue system methods		*0.038*		
Generative	N/A	N/A	N/A	0
Retrieval-based	-0.058 (-0.246 to 0.131)	0.549	0.0%	5
Rule-based	0.170 (0.066 to 0.274)	0.001	57.7%	5
**Self-efficacy**				
Intervention duration		0.060		
0-4 weeks	0.211 (0.073 to 0.350)	0.003	0.0%	2
5-8 weeks	0.087 (-0.753 to 0.926)	0.840	92.9%	3
⩾9 weeks	0.000 (-0.107 to 0.107)	1.000	N/A	1
Target sample		0.079		

Clinical	0.698 (-0.305 to 1.700)	0.173	N/A	1
Subclinical	0.484 (-0.106 to 1.073)	0.108	90.6%	2
Nonclinical	-0.164 (-0.519 to 0.190)	0.364	74.6%	3
Deployment		0.778		
Web-based	0.202 (0.062 to 0.342)	0.005	N/A	1
Standalone	0.136 (-0.300 to 0.572)	0.541	87.4%	5
Dialogue system methods		0.072		
Generative	N/A	N/A	N/A	0
Retrieval-based	-0.288 (-0.877 to 0.301)	0.337	75.1%	2
Rule-based	0.320 (0.015 to 0.626)	0.040	87.3%	4
**Health behavior change**				
Control group types		*0.025*		
Active control	-0.259 (-0.587 to 0.070)	0.123	0.0%	2
Information control	N/A	N/A	N/A	0
Passive control	0.129 (0.049 to 0.209)	0.002	41.1%	8
Intervention duration		0.548		
0-4 weeks	N/A	N/A	N/A	0
5-8 weeks	0.089 (-0.011 to 0.189)	0.080	57.5%	5
⩾9 weeks	0.138 (0.015 to 0.261)	0.028	40.5%	4
Target sample		0.893		
Clinical	0.082 (-0.356 to 0.520)	0.714	N/A	1
Subclinical	0.170 (-0.099 to 0.439)	0.216	88.8%	2
Nonclinical	0.104 (0.021 to 0.186)	0.014	11.5%	6
Reminders		*0.024*		
Yes	0.123 (0.045 to 0.202)	0.002	28.3%	8
No	-0.432 (-0.910 to 0.045)	0.076	N/A	1
Interaction mode		0.102		
Text-only	-0.153 (-0.476 to 0.170)	0.353	58.6%	2
Multimedia	0.125 (0.045 to 0.205)	0.002	38.3%	7
Deployment		0.903		
Web-based	0.082 (-0.356 to 0.520)	0.714	N/A	1
Standalone	0.109 (0.031 to 0.188)	0.007	52.7%	8
Dialogue system methods		0.903		
Generative	N/A	N/A	N/A	0
Retrieval-based	0.082 (-0.356 to 0.520)	0.714	N/A	1
Rule-based	0.109 (0.031 to 0.188)	0.007	52.7%	8

[a] Number of comparisons.

Table S5. GRADEpro GDT Summary of findings

Chatbot interventions compared to controls for mental health distress and health behavior change					
**Outcomes**	**№ of participants (studies) Follow-up**	**Certainty of the evidence (GRADE)**	**Relative effect (95% CI)**	**Anticipated absolute effects**	
				**Risk with Control**	**Risk difference with Chatbots**
Overall mental distress	5929 (21 RCTs)	⊕◯◯◯ Very low[a,b,c]	-	-	SMD **0.35 SD lower** (0.46 lower to 0.24 lower)
Depression	3183 (17 RCTs)	⊕◯◯◯ Very low[a,b,c]	-	-	SMD **0.43 SD lower** (0.62 lower to 0.23 lower)
Anxiety	4090 (18 RCTs)	⊕◯◯◯ Very low[a,b]	-	-	SMD **0.37 lower** (0.58 lower to 0.17 lower)
Stress	1752 (6 RCTs)	⊕⊕◯◯ Low[a]	-	-	SMD **0.41 lower** (0.5 lower to 0.31 lower)
Positive affect	1889 (11 RCTs)	⊕◯◯◯ Very low[a,b,d]	-	-	SMD **0.03 higher** (0.15 lower to 0.21 higher)
Negative affect	1889 (11 RCTs)	⊕◯◯◯ Very low[a,b]	-	-	SMD **0.27 lower** (0.53 lower to 0.01 lower)
Life satisfaction and well-being	1634 (7 RCTs)	⊕⊕◯◯ Low[a]	-	-	SMD **0.12 higher** (0.03 higher to 0.21 higher)
Self-ambivalence and appearance distress	1526 (3 RCTs)	⊕⊕◯◯ Low[a]	-	-	SMD **0.25 lower** (0.34 lower to 0.17 lower)
Self-efficacy	2505 (6 RCTs)	⊕◯◯◯ Very low[a,b,d]	-	-	SMD **0.14 higher** (0.14 lower to 0.41 higher)
Psychosomatic symptoms	652 (5 RCTs)	⊕◯◯◯ Very low[a,b]	-	-	SMD **0.48 lower** (0.82 lower to 0.14 lower)
Health behavior change	3228 (6 RCTs)	⊕⊕◯◯ Low[a]	-	-	SMD **0.11 higher** (0.03 higher to 0.19 higher)

Chatbot interventions compared to controls for mental health distress and health behavior change

Patient or population: Adolescents and young adults

Setting: Clinical and nonclinical settings

Intervention: Chatbots

Comparison: Active/information/passive controls

***The risk in the intervention group** (and its 95% confidence interval) is based on the assumed risk in the comparison group and the **relative effect** of the intervention (and its 95% CI).

CI: confidence interval; **SMD:** standardized mean difference

GRADE Working Group grades of evidence

High certainty: we are very confident that the true effect lies close to that of the estimate of the effect.

Moderate certainty: we are moderately confident in the effect estimate: the true effect is likely to be close to the estimate of the effect, but there is a possibility that it is substantially different.

Low certainty: our confidence in the effect estimate is limited: the true effect may be substantially different from the estimate of the effect.

Very low certainty: we have very little confidence in the effect estimate: the true effect is likely to be substantially different from the estimate of effect.

Explanations

a. the overall risk of bias for the majority of studies was rated as high

b. Substantial heterogeneity across studies

c. Potential publication bias

d. 95% CI crosses null effect

Figure S1. Forest plot for the effects of chatbots on depression

	Experimental			Control				Std. Mean Difference
Study or Subgroup	Mean	SD	Total	Mean	SD	Total	Weight	IV, Random, 95% CI
Chan, 2024	7.16	3.86	26	9.75	6.1	51	5.6%	-0.47 [-0.95, 0.01]
Fitzpatrick, 2017	11.14	4.14	34	13.67	4.86	36	5.6%	-0.55 [-1.03, -0.07]
Fitzsimmons-Craft, 2022	11.3	6.14	352	11.92	5.68	348	8.2%	-0.10 [-0.25, 0.04]
Greer, 2019	58.2	8.8	25	57.7	6.1	20	4.8%	0.06 [-0.52, 0.65]
He, 2022	7.58	2.01	45	8.62	1.87	39	6.0%	-0.53 [-0.97, -0.09]
Hunt, 2021	5.78	4.2	36	10.32	4.29	44	5.7%	-1.06 [-1.53, -0.59]
Jang, 2021	10.63	7.18	23	15	8.02	23	4.8%	-0.56 [-1.15, 0.03]
Karkosz, 2024	7.64	4.53	33	9.11	5.59	35	5.6%	-0.28 [-0.76, 0.19]
Liu, 2022	7.92	3.07	41	10.61	3.43	42	5.9%	-0.82 [-1.27, -0.37]
Nicol, 2022	6.8	6.9	10	12.9	5.4	7	2.5%	-0.91 [-1.94, 0.11]
Romanovsk, 2021	5.57	4.06	42	14.33	6.12	40	5.4%	-1.68 [-2.19, -1.17]
Sabour, 2023	15.66	5.75	142	16.65	5.39	105	7.5%	-0.18 [-0.43, 0.08]
Selaskowski, 2023	12.65	4.076	17	10.65	3.061	17	4.1%	0.54 [-0.14, 1.23]
Suharwardy, 2023	3.09	3.02	68	3.23	3.84	84	7.0%	-0.04 [-0.36, 0.28]
Ulrich, 2024 (BalanceUP)	6.77	3.55	78	9.35	5.15	72	6.9%	-0.58 [-0.91, -0.26]
Ulrich, 2024 (MISHA)	5.95	3.45	42	7.86	4.02	56	6.2%	-0.50 [-0.91, -0.09]
Vereschagin, 2024	8.6	6.2	563	8.8	5.8	587	8.4%	-0.03 [-0.15, 0.08]
**Total (95% CI)**			**1577**			**1606**	**100.0%**	**-0.43 [-0.62, -0.23]**

Heterogeneity: Tau² = 0.11; Chi² = 84.66, df = 16 (P < 0.00001); I² = 81%
Test for overall effect: Z = 4.35 (P < 0.0001)

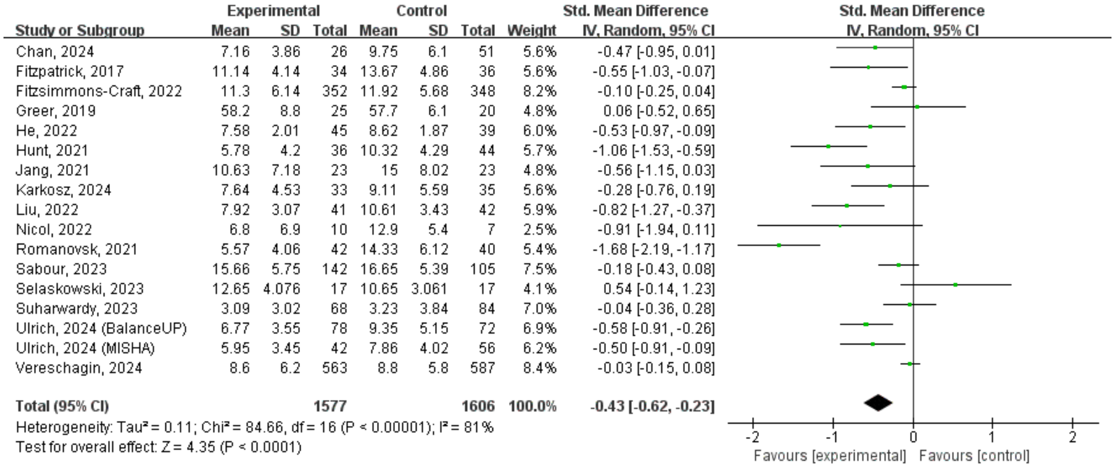

Figure S2. Forest plot for the effects of chatbots on anxiety

	Experimental			Control				Std. Mean Difference
Study or Subgroup	Mean	SD	Total	Mean	SD	Total	Weight	IV, Random, 95% CI
Chan, 2024	6.05	4.56	26	9.23	6.08	51	5.4%	-0.56 [-1.04, -0.08]
Fitzpatrick, 2017	17.35	3.5	34	16.84	4.02	36	5.4%	0.13 [-0.34, 0.60]
Fitzsimmons-Craft, 2022	10.12	6.12	352	10.77	5.71	348	7.4%	-0.11 [-0.26, 0.04]
Greer, 2019	61.9	7.7	25	63.3	5.5	20	4.7%	-0.20 [-0.79, 0.39]
Hunt, 2021	8.67	6.38	36	12.05	9.72	44	5.6%	-0.40 [-0.84, 0.05]
Jang, 2021	34.42	9.51	23	39.52	7.96	23	4.7%	-0.57 [-1.16, 0.02]
Karkosz, 2024	48.82	9.25	33	48.54	8.94	35	5.4%	0.03 [-0.45, 0.51]
Klos, 2021	13.04	7.12	27	16.26	5.79	23	4.8%	-0.48 [-1.05, 0.08]
Liu, 2022	14.23	2.18	41	14.97	2.72	42	5.7%	-0.30 [-0.73, 0.14]
Maeda, 2020	43.2	9.5	309	46.85	9.27	618	7.4%	-0.39 [-0.53, -0.25]
Nicol, 2022	7.4	6.5	10	11.7	4.8	7	2.7%	-0.69 [-1.70, 0.31]
Romanovsk, 2021	4.36	3.23	42	16.5	2.86	40	3.7%	-3.94 [-4.69, -3.18]
Sabour, 2023	14.41	4.7	142	14.45	4.08	105	6.9%	-0.01 [-0.26, 0.24]
Selaskowski, 2023	10.47	2.787	17	9.24	2.107	17	4.1%	0.49 [-0.20, 1.17]
Suharwardy, 2023	3.32	3.85	68	3.63	4.45	84	6.4%	-0.07 [-0.39, 0.25]
Ulrich, 2024 (BalanceUP)	4.72	2.41	78	7.07	4.53	72	6.4%	-0.65 [-0.98, -0.32]
Ulrich, 2024 (MISHA)	5.62	3.22	42	6.59	3.47	56	5.9%	-0.29 [-0.69, 0.12]
Vereschagin, 2024	7.7	5.1	575	8.2	5.1	589	7.5%	-0.10 [-0.21, 0.02]
**Total (95% CI)**			**1880**			**2210**	**100.0%**	**-0.37 [-0.58, -0.17]**

Heterogeneity: Tau² = 0.14; Chi² = 128.85, df = 17 (P < 0.00001); I² = 87%
Test for overall effect: Z = 3.60 (P = 0.0003)

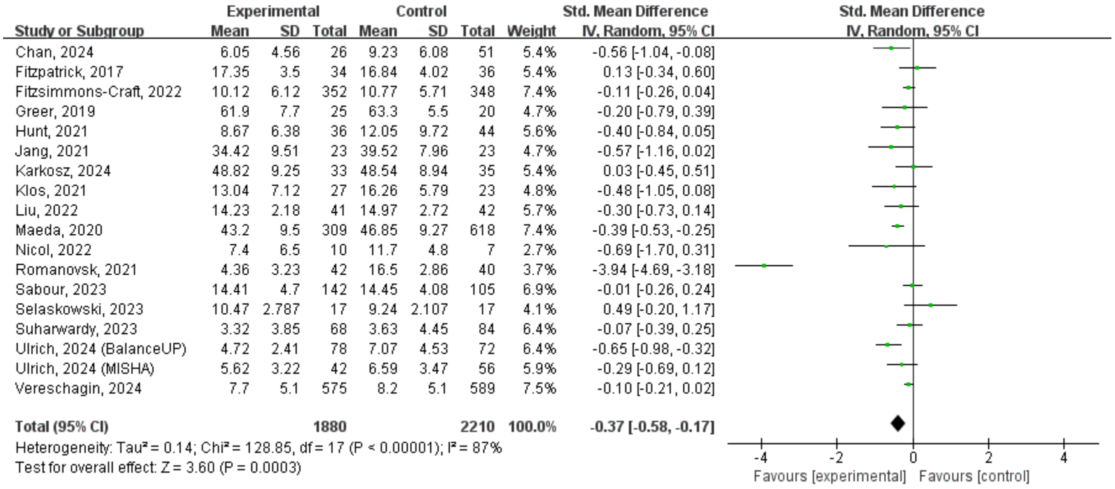

Figure S3. Forest plot for the effects of chatbots on positive affect

	Experimental			Control				Std. Mean Difference
Study or Subgroup	Mean	SD	Total	Mean	SD	Total	Weight	IV, Random, 95% CI
Drouin, 2022	24.79	9.81	132	26.35	7.31	146	13.0%	-0.18 [-0.42, 0.05]
Ehrlich, 2024 (1)	3.19	0.65	33	2.98	0.87	51	8.3%	0.26 [-0.18, 0.70]
Ehrlich, 2024 (2)	3.01	0.66	45	3.23	0.61	61	9.4%	-0.35 [-0.73, 0.04]
Fitzpatrick, 2017	26.88	7.52	34	26.02	8.7	36	7.8%	0.10 [-0.36, 0.57]
Greer, 2019	2.5	1	25	2.3	0.8	20	5.9%	0.21 [-0.38, 0.80]
Karkosz, 2024	20.27	6.29	33	20.66	7.09	35	7.6%	-0.06 [-0.53, 0.42]
Lavelle, 2021	22.02	8.42	20	28	7.98	8	3.5%	-0.70 [-1.54, 0.14]
Liu, 2022	28.29	1.73	41	28.65	2.4	42	8.5%	-0.17 [-0.60, 0.26]
Matheson, 2023	3.72	0.93	355	3.55	0.94	443	15.3%	0.18 [0.04, 0.32]
Romanovsk, 2021	33.64	8.94	42	27.43	6.36	40	8.1%	0.79 [0.34, 1.24]
Sabour, 2023	23.99	6.57	142	24.21	8.05	105	12.6%	-0.03 [-0.28, 0.22]
**Total (95% CI)**			**902**			**987**	**100.0%**	**0.03 [-0.15, 0.21]**

Heterogeneity: Tau² = 0.05; Chi² = 27.25, df = 10 (P = 0.002); I² = 63%
Test for overall effect: Z = 0.34 (P = 0.73)

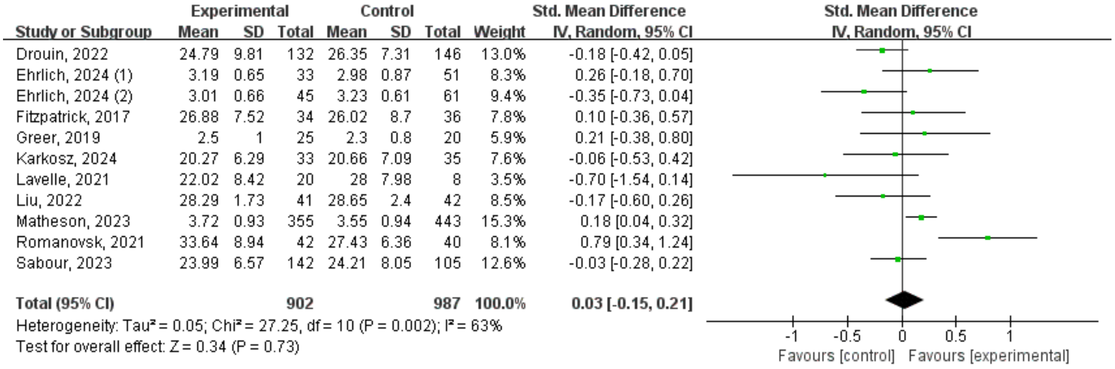

Figure S4. Forest plot for the effects of chatbots on negative affect

Study or Subgroup	Experimental Mean	Experimental SD	Experimental Total	Control Mean	Control SD	Control Total	Weight	Std. Mean Difference IV, Random, 95% CI
Drouin, 2022	12.8	4.45	132	13.12	3.43	146	11.1%	-0.08 [-0.32, 0.15]
Ehrlich, 2024 (1)	2.39	0.97	33	2.37	0.77	51	9.1%	0.02 [-0.41, 0.46]
Ehrlich, 2024 (2)	2.41	0.67	45	2.34	0.68	61	9.6%	0.10 [-0.28, 0.49]
Fitzpatrick, 2017	25.98	7.23	34	27.53	8.52	36	8.7%	-0.19 [-0.66, 0.28]
Greer, 2019	1.5	0.9	25	1.6	0.6	20	7.5%	-0.13 [-0.71, 0.46]
Karkosz, 2024	27	7.26	33	29.77	8.76	35	8.6%	-0.34 [-0.82, 0.14]
Lavelle, 2021	21.33	9.96	20	22.71	10.75	8	5.5%	-0.13 [-0.95, 0.69]
Liu, 2022	27.8	4.93	41	27.27	7.26	42	9.2%	0.08 [-0.35, 0.51]
Matheson, 2023	2.34	1.03	355	2.45	1.01	443	11.8%	-0.11 [-0.25, 0.03]
Romanovsk, 2021	16.88	6.65	42	32.2	6.83	40	7.8%	-2.25 [-2.81, -1.69]
Sabour, 2023	20.94	6.59	142	22.89	7.73	105	11.0%	-0.27 [-0.53, -0.02]
**Total (95% CI)**			**902**			**987**	**100.0%**	**-0.27 [-0.53, -0.01]**

Heterogeneity: Tau² = 0.14; Chi² = 59.82, df = 10 (P < 0.00001); I² = 83%
Test for overall effect: Z = 2.04 (P = 0.04)

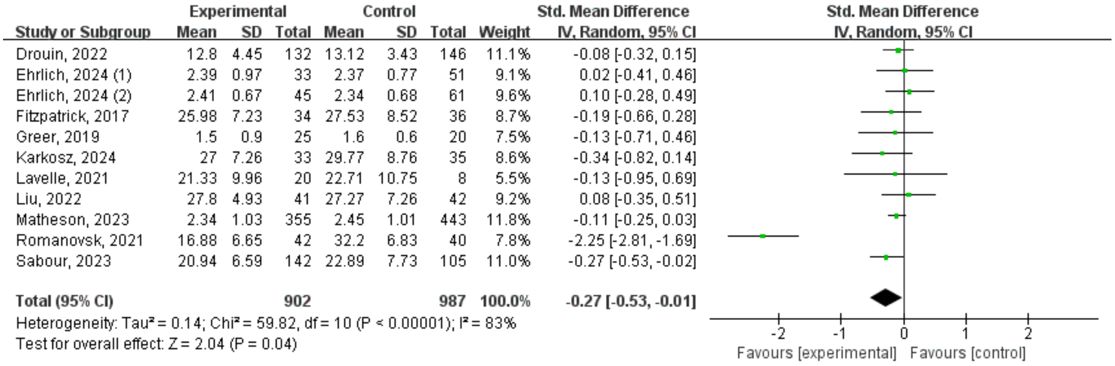

Figure S5. Forest plot for the effects of chatbots on stress

Study or Subgroup	Experimental Mean	Experimental SD	Experimental Total	Control Mean	Control SD	Control Total	Weight	Std. Mean Difference IV, Fixed, 95% CI
Haug, 2022	2.7	1.1	688	3.1	1.1	663	77.7%	-0.36 [-0.47, -0.26]
Hunt, 2021	12.72	8.65	36	18.82	9.99	44	4.4%	-0.64 [-1.09, -0.19]
Jang, 2021	19.16	6.81	23	23.61	8.17	23	2.6%	-0.58 [-1.17, 0.01]
Ly, 2017	12.93	5.2	14	17.14	4.4	14	1.5%	-0.85 [-1.63, -0.07]
Ulrich, 2024 (BalanceUP)	26.9	6.06	78	29.79	7.34	71	8.5%	-0.43 [-0.75, -0.10]
Ulrich, 2024 (MISHA)	24.24	5.93	42	27.61	5.38	56	5.4%	-0.59 [-1.00, -0.19]
**Total (95% CI)**			**881**			**871**	**100.0%**	**-0.41 [-0.50, -0.31]**

Heterogeneity: Chi² = 4.06, df = 5 (P = 0.54); I² = 0%
Test for overall effect: Z = 8.40 (P < 0.00001)

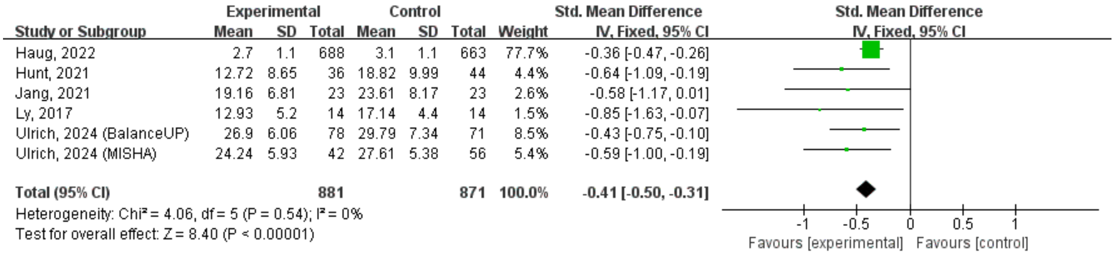

Figure S6. Forest plot for the effects of chatbots on psychosomatic symptoms

Study or Subgroup	Experimental Mean	Experimental SD	Experimental Total	Control Mean	Control SD	Control Total	Weight	Std. Mean Difference IV, Random, 95% CI
Chan, 2024	9.42	4.44	26	11.75	5.3	51	17.6%	-0.46 [-0.94, 0.02]
Hunt, 2021	27.56	10.12	36	38.18	10.79	44	17.8%	-1.00 [-1.47, -0.53]
Sabour, 2023	15.79	5.76	142	15.97	5.92	105	23.5%	-0.03 [-0.28, 0.22]
Ulrich, 2024 (BalanceUP)	8.42	3.66	78	11.42	5.46	72	21.5%	-0.65 [-0.98, -0.32]
Ulrich, 2024 (MISHA)	7.5	3.78	42	9	4.43	56	19.6%	-0.36 [-0.76, 0.05]
**Total (95% CI)**			**324**			**328**	**100.0%**	**-0.48 [-0.82, -0.14]**

Heterogeneity: Tau² = 0.11; Chi² = 16.79, df = 4 (P = 0.002); I² = 76%
Test for overall effect: Z = 2.74 (P = 0.006)

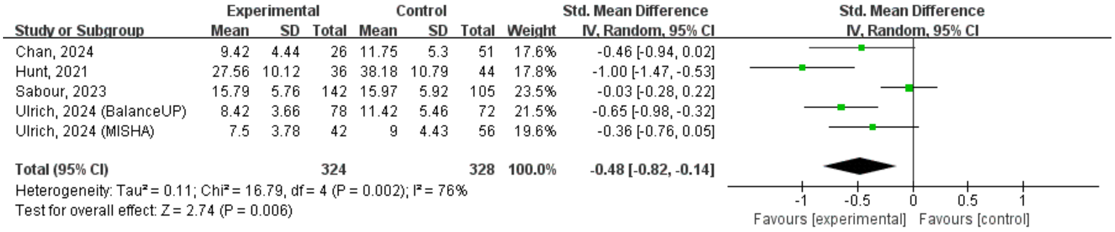

Figure S7. Forest plot for the effects of chatbots on self-ambivalence and appearance distress

Study or Subgroup	Experimental Mean	Experimental SD	Experimental Total	Control Mean	Control SD	Control Total	Weight	Std. Mean Difference IV, Fixed, 95% CI
Fitzsimmons-Craft, 2022	15.48	3.62	352	16.76	3.07	348	31.4%	-0.38 [-0.53, -0.23]
Fitzsimmons-Craft, 2022	61.12	19.08	352	65.57	17.27	348	31.7%	-0.24 [-0.39, -0.10]
Lavelle, 2021	31.15	7.91	20	31.71	8.44	8	1.0%	-0.07 [-0.89, 0.75]
Matheson, 2023	1.54	1.07	355	1.71	1.09	443	35.9%	-0.16 [-0.30, -0.02]
**Total (95% CI)**			**1079**			**1147**	**100.0%**	**-0.25 [-0.34, -0.17]**

Heterogeneity: Chi² = 4.82, df = 3 (P = 0.19); I² = 38%
Test for overall effect: Z = 5.94 (P < 0.00001)

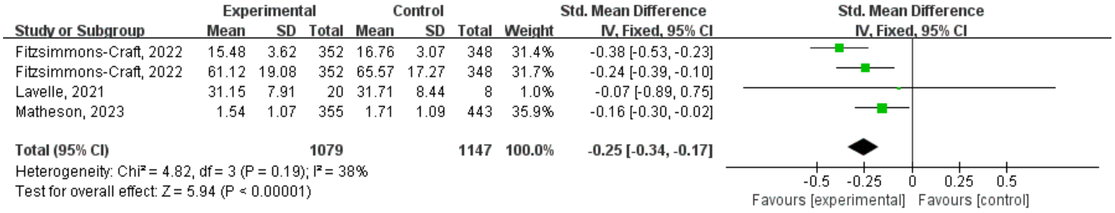

Figure S8. Forest plot for the effects of chatbots on life satisfaction and well-being

Study or Subgroup	Experimental Mean	Experimental SD	Experimental Total	Control Mean	Control SD	Control Total	Weight	Std. Mean Difference IV, Fixed, 95% CI
Chan, 2024	18	6.72	26	16.85	5.82	51	3.7%	0.19 [-0.29, 0.66]
Ehrlich, 2024 (1)	3.31	0.62	33	3.26	0.72	51	4.3%	0.07 [-0.37, 0.51]
Ehrlich, 2024 (1)	4.73	1.11	33	4.56	1.25	51	4.3%	0.14 [-0.30, 0.58]
Ehrlich, 2024 (2)	3.16	0.6	45	3.28	0.7	61	5.6%	-0.18 [-0.57, 0.21]
Ehrlich, 2024 (2)	4.55	1.17	45	4.59	1.04	61	5.6%	-0.04 [-0.42, 0.35]
Karkosz, 2024	18.97	7.03	33	20.91	6.07	35	3.6%	-0.29 [-0.77, 0.19]
Ly, 2017	26.79	6	14	26	4.6	14	1.5%	0.14 [-0.60, 0.89]
Ly, 2017	45.14	6	14	45.07	5.7	14	1.5%	0.01 [-0.73, 0.75]
Ulrich, 2024 (BalanceUP)	36.51	5.48	78	31.58	9.18	72	7.7%	0.66 [0.33, 0.98]
Vereschagin, 2024	22.3	4.5	567	21.8	4.3	587	62.2%	0.11 [-0.00, 0.23]
**Total (95% CI)**			**888**			**997**	**100.0%**	**0.12 [0.03, 0.21]**

Heterogeneity: Chi² = 16.21, df = 9 (P = 0.06); I² = 44%
Test for overall effect: Z = 2.51 (P = 0.01)

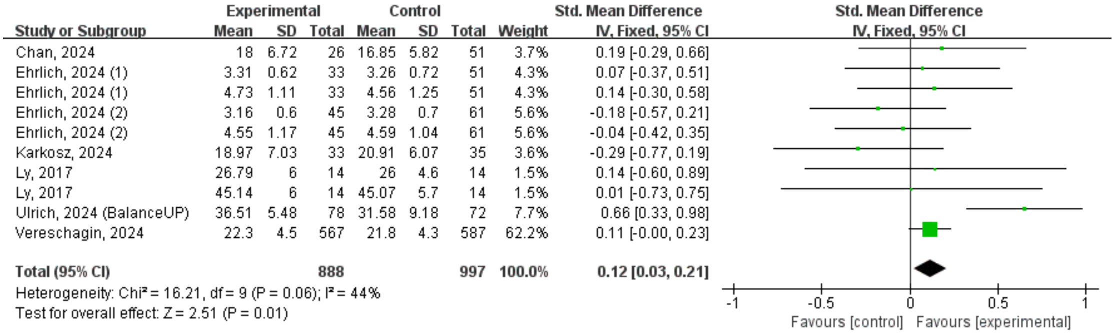

Figure S9. Forest plot for the effects of chatbots on self-efficacy

Study or Subgroup	Experimental Mean	Experimental SD	Experimental Total	Control Mean	Control SD	Control Total	Weight	Std. Mean Difference IV, Random, 95% CI
Ehrlich, 2024 (1)	2.92	0.49	33	2.91	0.49	51	14.9%	0.02 [-0.42, 0.46]
Ehrlich, 2024 (2)	2.85	0.33	45	3.09	0.46	61	16.1%	-0.58 [-0.97, -0.19]
Haug, 2022	3.8	0.8	688	3.8	0.7	663	23.1%	0.00 [-0.11, 0.11]
Matheson, 2023	70.44	21.7	355	65.92	22.87	443	22.5%	0.20 [0.06, 0.34]
Nicol, 2022	38.7	8.4	10	31.7	11	7	5.8%	0.70 [-0.30, 1.70]
Ulrich, 2024 (BalanceUP)	28.58	7.21	78	23.07	6.34	71	17.7%	0.81 [0.47, 1.14]
**Total (95% CI)**			**1209**			**1296**	**100.0%**	**0.14 [-0.14, 0.41]**

Heterogeneity: Tau² = 0.08; Chi² = 35.43, df = 5 (P < 0.00001); I² = 86%
Test for overall effect: Z = 0.98 (P = 0.33)

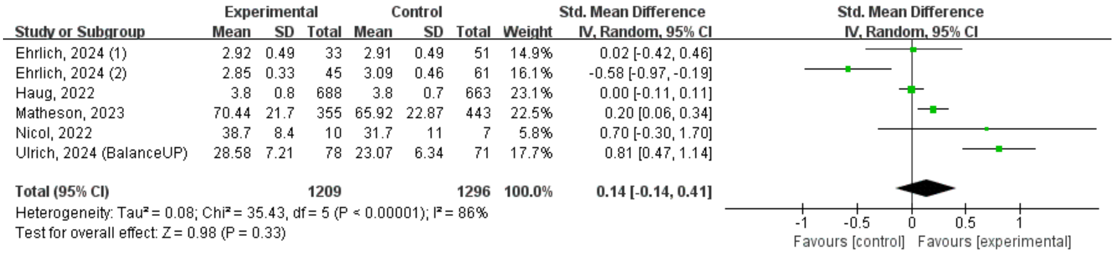

Figure S10. Forest plot for the effects of chatbots on health behavior change

Study or Subgroup	Std. Mean Difference	SE	Weight	Std. Mean Difference IV, Fixed, 95% CI
Bricker, 2024	0.081828	0.223334	3.1%	0.08 [-0.36, 0.52]
Chan, 2024	-0.4321	0.2436	2.6%	-0.43 [-0.91, 0.05]
Haug, 2022	0.227208	0.12900108	9.4%	0.23 [-0.03, 0.48]
Haug, 2022	0.148874	0.12174969	10.6%	0.15 [-0.09, 0.39]
Haug, 2022	-0.1441	0.1440127	7.6%	-0.14 [-0.43, 0.14]
Haug, 2022	0.234462	0.11359975	12.2%	0.23 [0.01, 0.46]
Ulrich, 2024 (BalanceUP)	0.4496	0.1661	5.7%	0.45 [0.12, 0.78]
Ulrich, 2024 (MISHA)	0.1293	0.2043	3.8%	0.13 [-0.27, 0.53]
Vereschagin, 2024	0.0716	0.059	45.1%	0.07 [-0.04, 0.19]
**Total (95% CI)**			**100.0%**	**0.11 [0.03, 0.19]**

Heterogeneity: Chi² = 14.82, df = 8 (P = 0.06); I² = 46%
Test for overall effect: Z = 2.74 (P = 0.006)

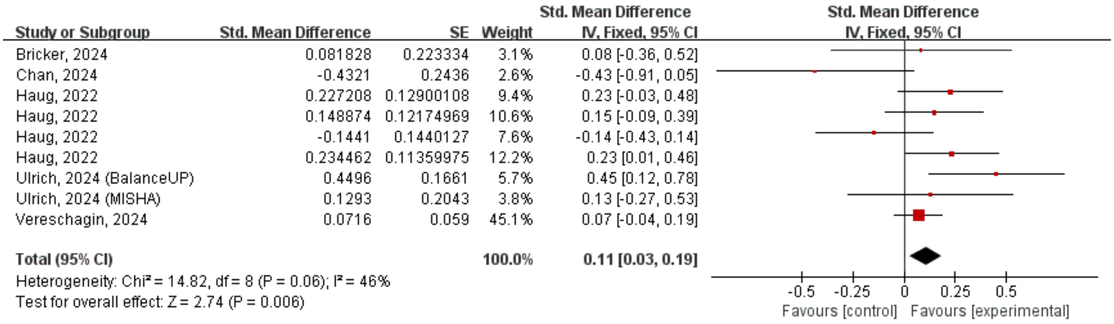

Figure S11. Leave-one-out sensitivity analysis plots for the effects of chatbots

a) Overall mental distress

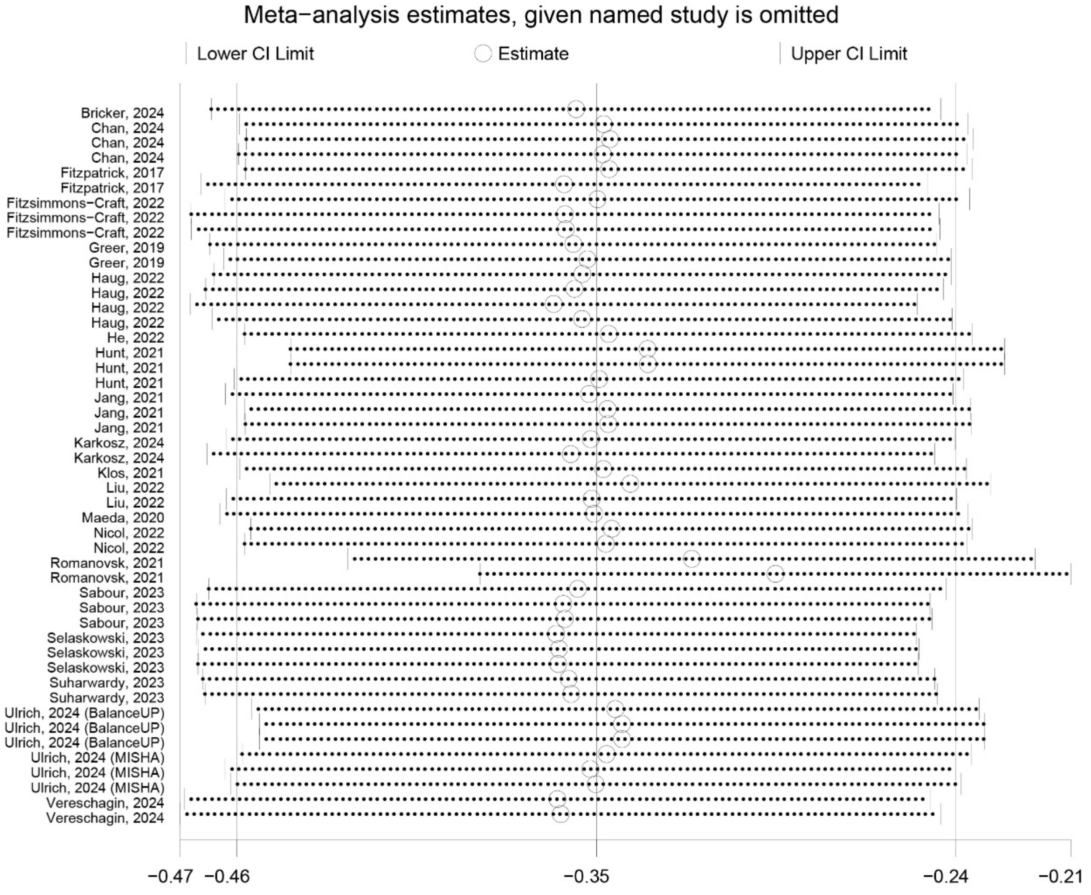

b) Depression

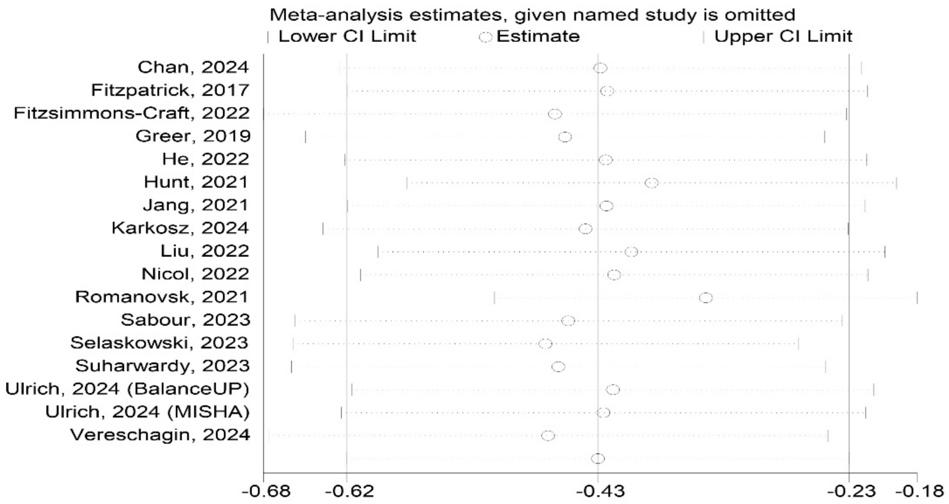

c) Anxiety

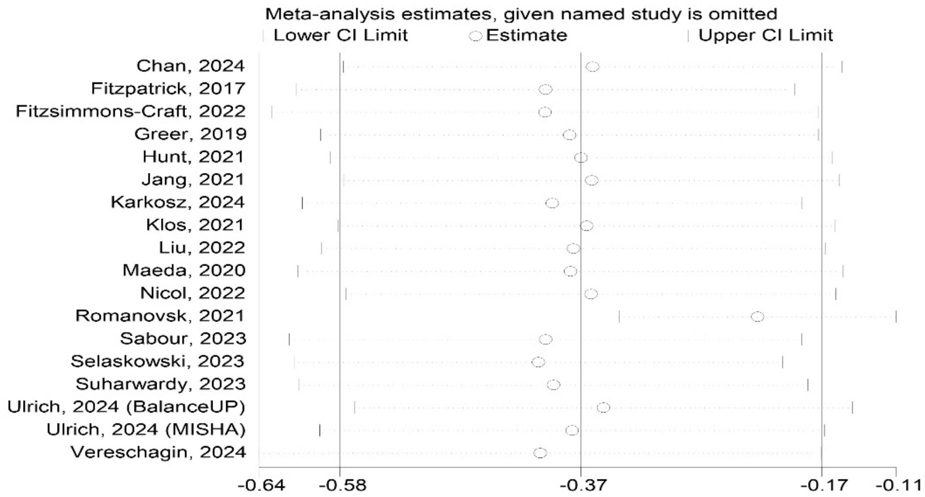

d) Positive affect

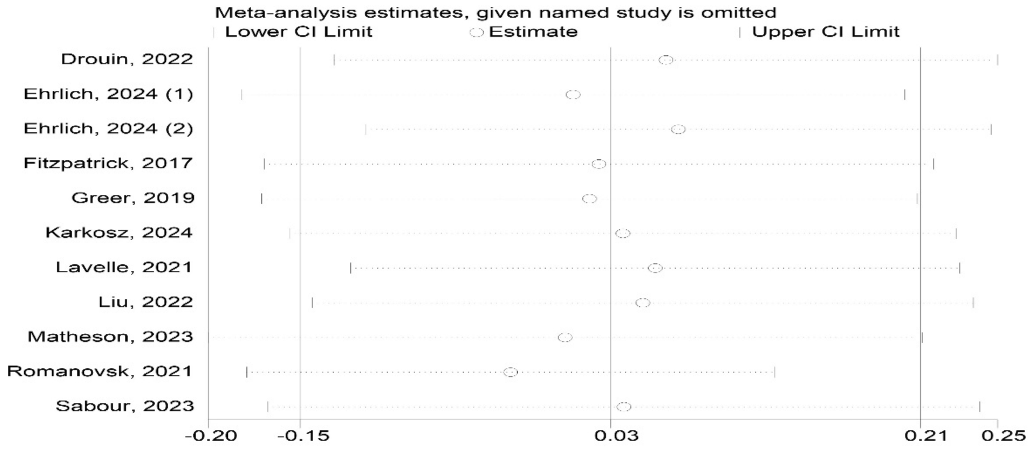

e) Negative affect

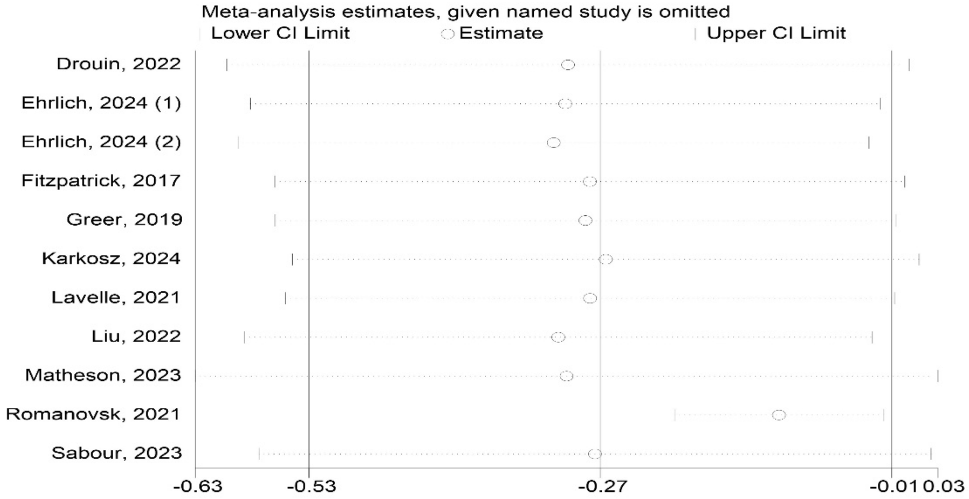

f) Stress

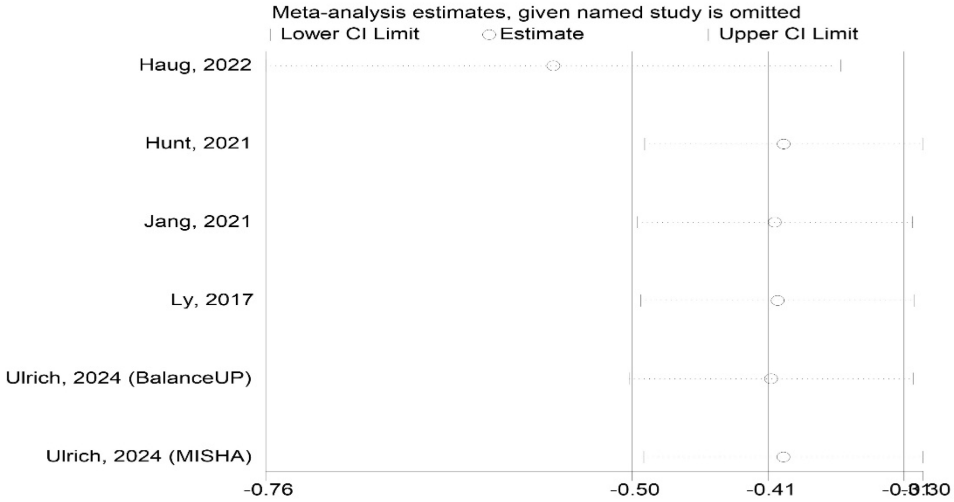

g) Psychosomatic symptoms

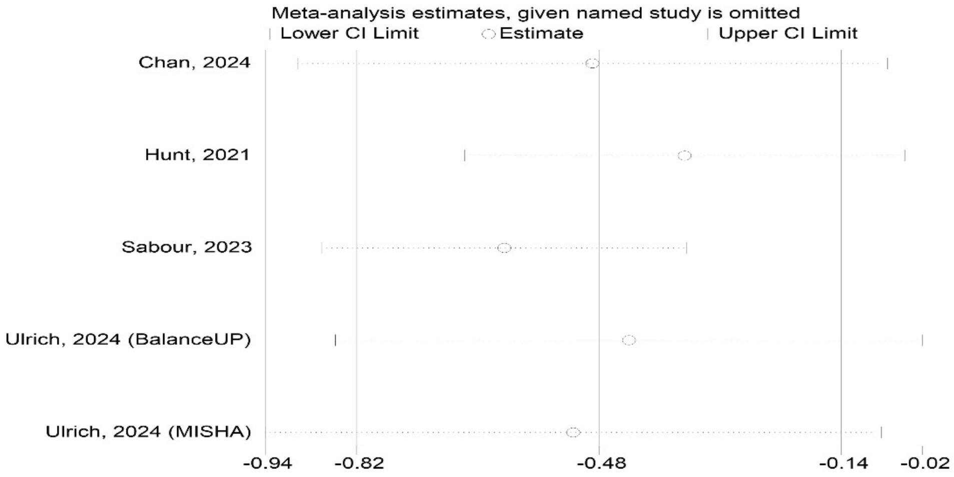

h) Self-ambivalence and appearance distress

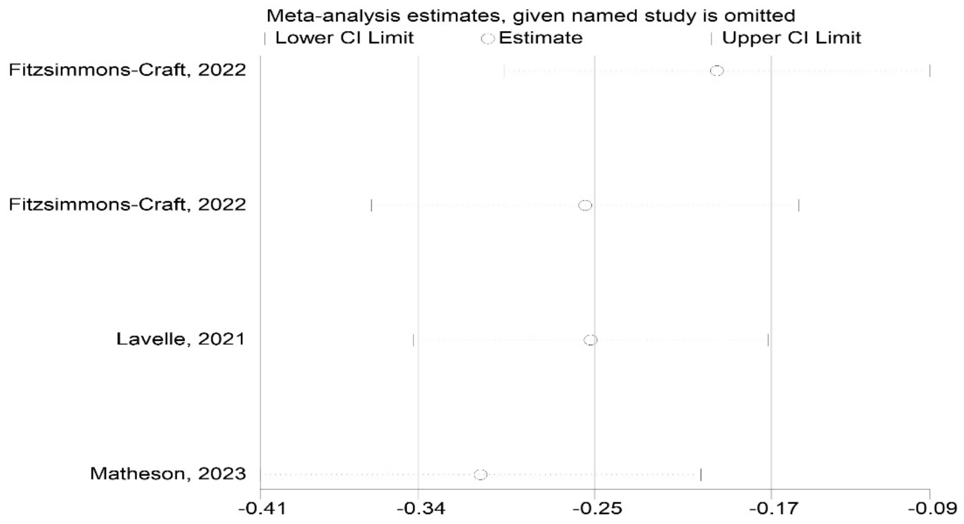

i) Life satisfaction and well-being

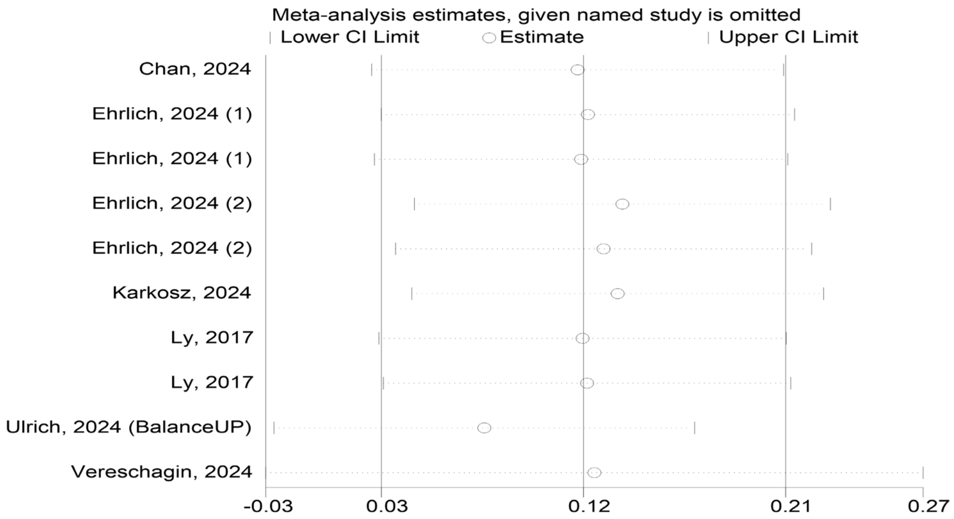

j) Self-efficacy

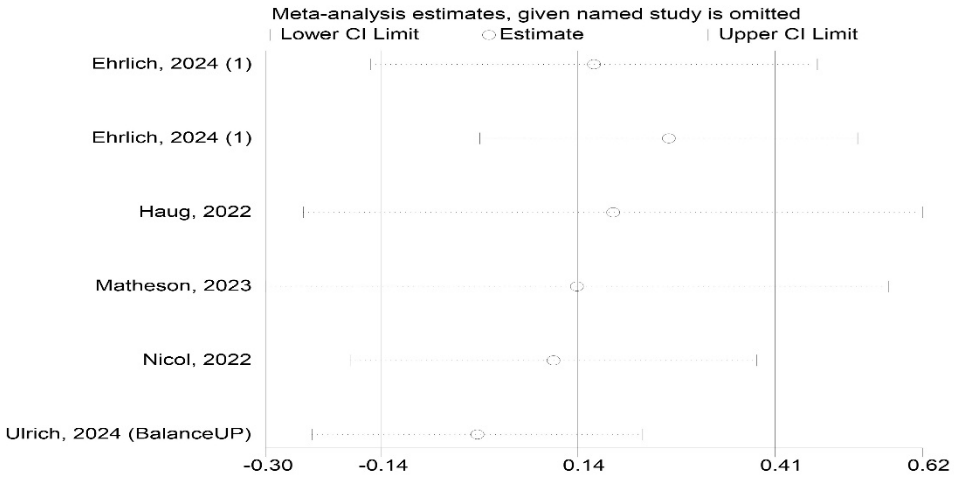

k) Health behavior change

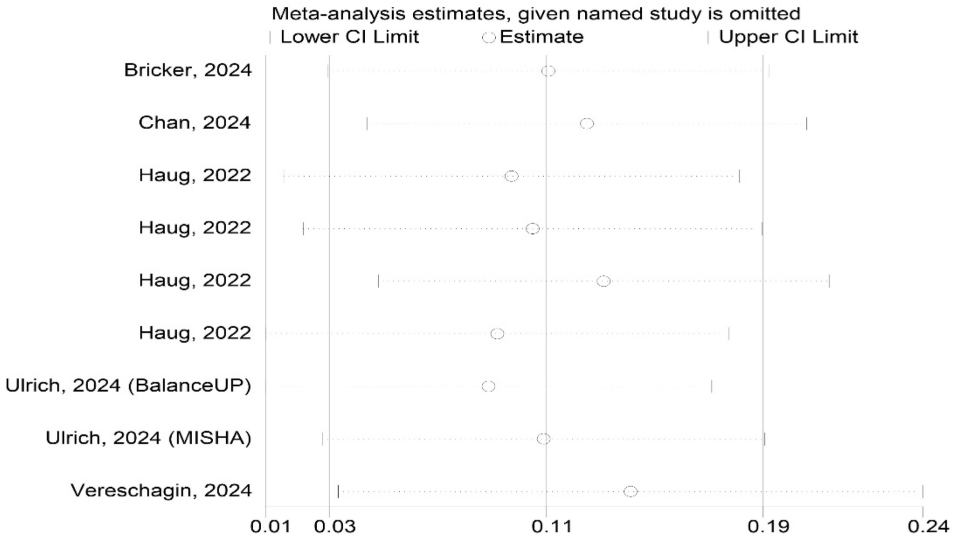

Figure S12. Bubble diagram of the significant result of meta-regression

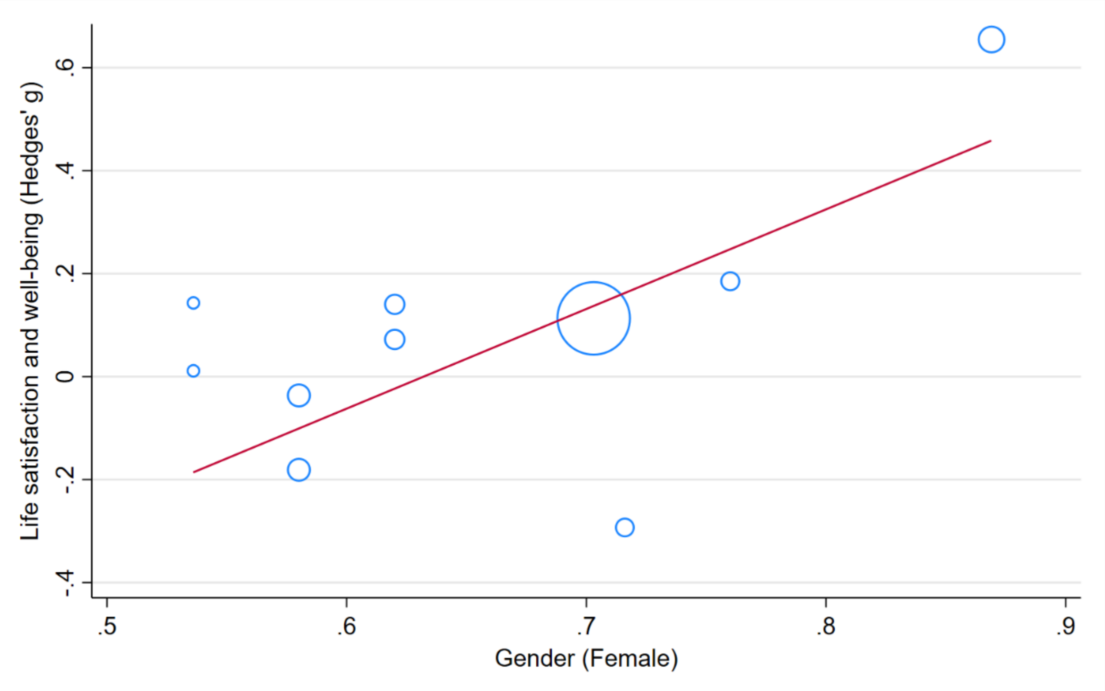

Figure S13. Risk of bias summary

Unique ID	D1	D2	D3	D4	D5	Overall
Bricker, 2024	+	!	+	-	+	-
Chan, 2024	+	+	-	!	+	-
Lee, 2023	+	+	-	+	+	-
De Filippo, 2023	+	-	+	!	-	-
Drouin, 2022	+	!	+	!	!	!
Ehrlich, 2024	!	-	-	-	+	-
Fitzpatrick, 2017	+	+	-	!	!	-
Fitzsimmons-Craft, 2022	+	+	-	-	+	-
Fulmer, 2018	+	!	+	!	+	!
Greer, 2019	+	-	!	-	!	-
Haug, 2022	+	+	!	-	+	-
He, 2022	+	+	-	!	+	-
Hunt, 2021	!	+	+	-	+	-
Jang, 2021	+	+	+	!	!	!
Karkosz, 2024	+	!	!	!	+	!
Klos, 2021	+	-	-	!	-	-
Lavelle, 2021	+	-	-	-	!	-
Liu, 2022	+	+	-	!	!	-
Ly, 2017	+	+	+	-	!	-
Maeda, 2020	+	+	+	!	+	!
Matheson, 2023	+	+	-	-	+	-
Nicol, 2022	+	!	+	-	+	-
Romanovsk, 2021	!	!	-	!	!	-
Sabour, 2023	+	+	-	-	!	-
Schillings, 2024	+	+	!	-	!	-
Selaskowski, 2023	+	!	!	+	!	!
Stewart, 2023	+	-	-	-	+	-
Suharwardy, 2023	!	-	-	!	+	-
Ulrich (BalanceUP), 2024	+	+	+	-	+	-
Ulrich (MISHA), 2024	+	+	+	-	+	-
Vereschagin, 2024	!	!	+	-	!	-

+ Low risk
! Some concerns
- High risk

D1 Randomisation process
D2 Deviations from the intended interventions
D3 Missing outcome data
D4 Measurement of the outcome
D5 Selection of the reported result

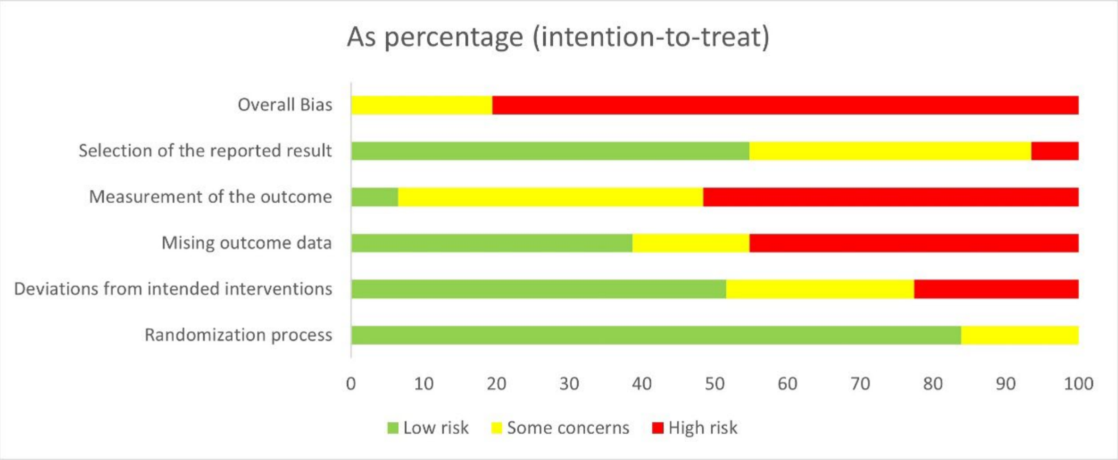

Supplement: Multimedia Appendix 1 [file jmir-v27-e79850-s001.pdf]
